# Reductive Conversion Leads to Detoxification of Salicortin-like Chemical Defenses (Salicortinoids) in Lepidopteran Specialist Herbivores (Notodontidae)

**DOI:** 10.1007/s10886-023-01423-4

**Published:** 2023-05-16

**Authors:** Florian Schnurrer, Christian Paetz

**Affiliations:** Department NMR/Biosynthesis, Max-Planck-Institute for Chemical Ecology, Hans-Knöll-Straße 8, 07745 Jena, Germany

**Keywords:** *Cerura vinula*, *Cerura erminea*, *Clostera anachoreta*, *Furcula furcula*, *Notodonta ziczac*, *Pheosia tremula*, Salicortinoids, Salicaceae, Detoxification, Stable isotope labeling

## Abstract

Lepidopteran specialist herbivores of the Notodontidae family have adapted to thrive on poplar and willow species (Salicaceae). Previous research showed that *Cerura vinula*, a member of the Notodontidae family occurring throughout Europe and Asia, uses a unique mechanism to transform salicortinoids, the host plant’s defense compounds, into quinic acid-salicylate conjugates. However, how the production of this conjugates relates to the detoxification of salicortinoids and how this transformation proceeds mechanistically have remained unknown. To find the mechanisms, we conducted gut homogenate incubation experiments with *C. vinula* and re-examined its metabolism by analyzing the constituents of its frass. To estimate the contribution of spontaneous degradation, we examined the chemical stability of salicortinoids and found that salicortinoids were degraded very quickly by midgut homogenates and that spontaneous degradation plays only a marginal role in the metabolism. We learned how salicortinoids are transformed into salicylate after we discovered reductively transformed derivatives, which were revealed to play key roles in the metabolism. Unless they have undergone the process of reduction, salicortinoids produce toxic catechol. We also studied constituents in the frass of the Notodontidae species *Cerura erminea*, *Clostera anachoreta*, *Furcula furcula*, *Notodonta ziczac*, and *Pheosia tremula*, and found the same metabolites as those described for *C. vinula*. We conclude that the process whereby salicortinoids are reductively transformed represents an important adaption of the Notodontidae to their Salicaceae host species.

**Supplementary Information:**

The online version contains supplementary material available at 10.1007/s10886-023-01423-4.

## Introduction

**Fig. 1:**
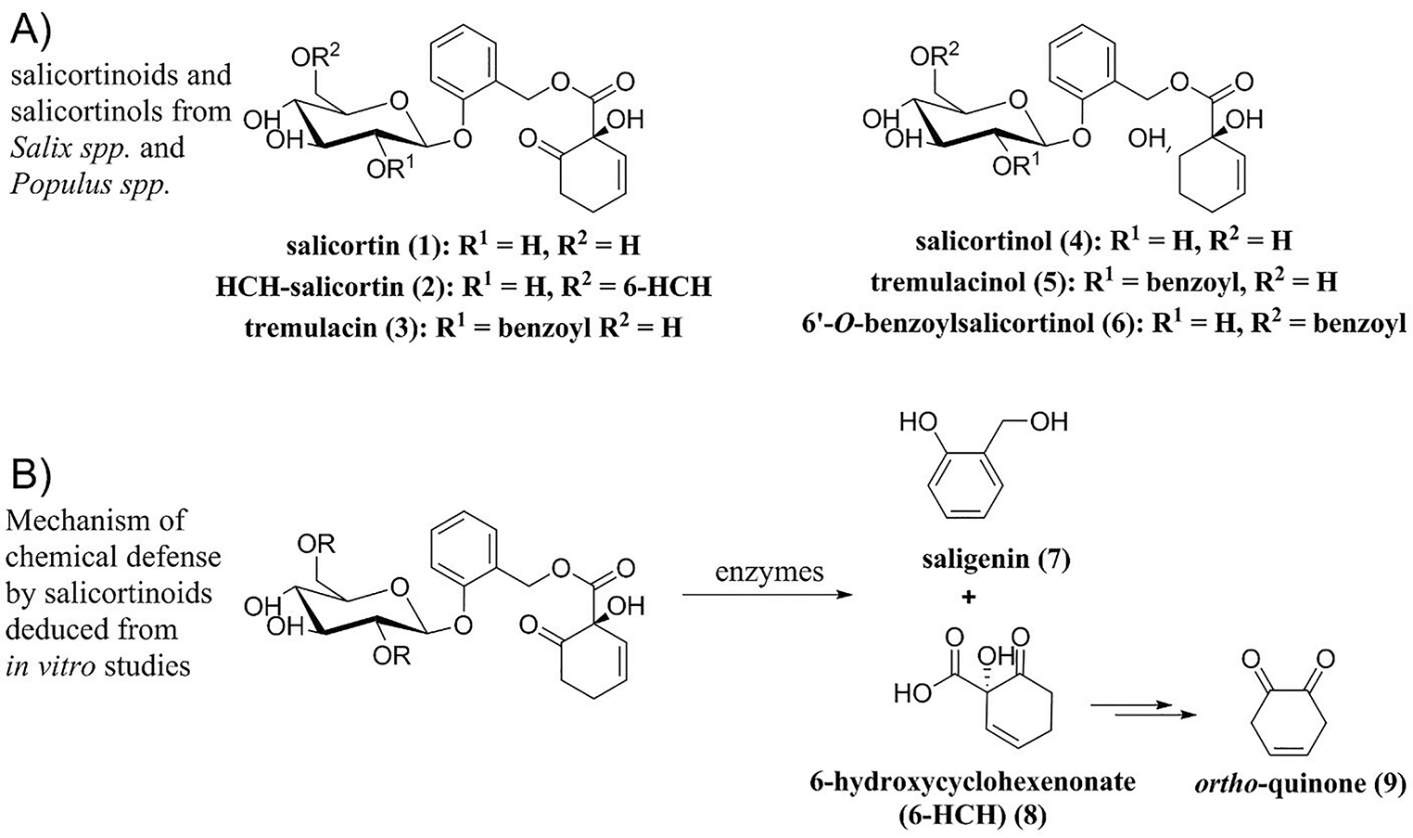
Naturally occurring salicortinoids and salicortinols (**A**). Proposed mechanism for how salicortinoids break down into toxic products (**B**)

Plants of the genera *Salix* and *Populus* are chemically protected against herbivory by salicinoids. By definition, salicinoids contain saligenin (**7**) substituted with a *β*-glucosyl moiety on its phenolic position. This glycosyl rest may contain another substitution in the 2’ or the 6’ position. In many salicinoids, the remaining benzylic position of saligenin (**7**) is esterified with common acids such as benzoic acid, salicylic acid, or the structurally more complex 1-hydroxy-6-oxocyclohex-2-ene-1-carboxylic acid (6-HCH) (Boeckler et al. 2011). Salicinoids containing the 6-HCH-moiety will hereafter be referred to as *salicortinoids* (Fig. 1A). How ingestion by the herbivore transforms the salicortinoids determines how the derived products act against the herbivore. Commonly, de-glucosylation occurs as salicortinoids pass through the insect gut, leading to the excretion of the aglycons (Lindroth 1988; Pentzold et al. 2014). Chemically reactive structures will be further transformed. In vitro studies showed that salicortinoids are not stable under acidic conditions, where they dehydrate and autoxidize to salicylate; under alkaline conditions, in contrast, ester cleavage, decarboxylation, and the subsequent transformation of the salicortinoids into catechol occurred (Julkunen-Tiitto and Meier 1992; Pearl and Darling 1971; Ruuhola et al. 2003). Catechol (**13**) and its oxidized form, *ortho*-quinone (**9**), were therefore considered the principal toxin of plant-defense systems based on salicortinoids (Appel 1993; Haruta et al. 2001), and high levels of salicortinoids were shown to reduce the growth of lepidopteran larvae (Fig. 1B) (Hemming and Lindroth 2000; Osier and Lindroth 2001; Ruuhola et al. 2001).

Recent in vivo studies with the generalist lepidopteran herbivore *Lymantria dispar* demonstrated that metabolic breakdown of salicortinoids led to the accumulation of catechol, which was in turn metabolized to catechol glucoside, catechol glucoside phosphate, and *N*-acetylcystein catechol adducts (Boeckler et al. 2016). However, studies with the lepidopteran specialist herbivore *Cerura vinula* showed that only quinic acid conjugates with salicylic acid and benzoic acid were produced by salicortinoid metabolism. Also, *C. vinula* larvae performed better than *L. dispar* when raised on a salicortinoid-rich diet (Feistel et al. 2017), which suggests its metabolism has been specially adapted. Although a mechanism for the salicortinoid conversion was proposed, no metabolic intermediates explaining the breakdown of salicortinoids were identified (Feistel et al. 2018). To date, no data about the salicortinoid metabolism in other members of the Notodontidae have been published. However, the adaption of Notodontidae to Salicaceae suggests the metabolism of their host plant’s defense compounds may be similar.

Here we report on the detailed degradation mechanism for salicortinoids. In our study, we used uniformly ^13^C-labeled compounds to avoid possible interference with metabolites sequestered by the insect. We also estimated the contribution of passive degradation examining the chemical stability of metabolites. Furthermore, we compared the constituents in the frass of six Notodontidae larvae (*C. vinula, C. erminea*, *C. anachoreta*, *F. furcula*, *N. ziczac*, and *P. tremula*) to determine whether they share a similar salicortinoid metabolism.

## Methods and Materials

### General Methods

Nuclear magnetic resonance (NMR) spectra were recorded either on a Bruker Avance III HD 700 MHz spectrometer, equipped with a cryoplatform and a 1.7 mm TCI microcryoprobe, or on a Bruker Avance III HD 500 MHz NMR spectrometer, equipped with a cryoplatform and a 5 mm TCI cryoprobe (Bruker Biospin GmbH, Rheinstetten, Germany). All NMR spectra were recorded at 298 K with MeOH-d_3_ as a solvent. Chemical shifts were referenced to the residual solvent peaks at δ_H_ 3.31 and δ_C_ 49.15. Data acquisition and processing were accomplished using Bruker TopSpin ver.3.6.1. Standard pulse programs as implemented in Bruker TopSpin ver.3.6.1. were used.

High-performance liquid chromatography coupled to high-resolution electrospray ionization mass spectrometry (HPLC-HR-ESI-MS) analyses were performed on an Agilent Infinity 1260 system, consisting of a combined degasser/quaternary pump G1311B, an autosampler G1367E, a column oven G1316A, and a photodiode array detector G1315D (Agilent Technologies GmbH, Waldbronn, Germany) connected to a Bruker Compact QTOF mass spectrometer (Bruker Daltonics GmbH, Bremen, Germany). Standard parameters for small-molecule analysis were used as implemented in Bruker Compass ver.1.9. Data analysis was accomplished using Compass DataAnalysis ver.4.4. Samples were measured in negative ionization mode using a mass range of *m/z* 100 to *m/z* 700. An Agilent Poroshell 120 EC C-18 column, 2.7 μm, 4.6 × 50 mm, equipped with a Phenomenex SecurityGuard Cartridge C18, 4 × 3 mm (Phenomenex Ltd., Aschaffenburg, Germany), was used for separations. A binary solvent system of H_2_O (solvent A) and acetonitrile (solvent B), both solvents containing 0.1% (v/v) formic acid, was used. The flow rate was set to 500 µl min^− 1^. The linear gradient started with 20% B and increased to 70% B within 13 min. The column was washed for 10 min with 100% B and re-equilibrated at 20% B for 5 min.

Preparative HPLC separations were accomplished using an Agilent 1100 HPLC system, consisting of a degasser G1322A, a binary pump G1312A, an autosampler G1313A, and a photodiode array detector G1315B. The column outlet was connected to an Advantec CHF122SB fraction collector (Advantec Toyo Kaisha Ltd., Tokyo, Japan) triggered by the HPLC via a relay contact board. All preparative HPLC separations were carried out using Macherey-Nagel (MN) columns (Macherey-Nagel GmbH & Co. KG, Düren, Germany). Medium-pressure chromatographic (MPLC) separations were accomplished using a Biotage Isolera One chromatograph (Biotage Sweden AB, Uppsala, Sweden) using linear gradient elution on a 30 g Biotage Sfär C18 Duo column (solvents H_2_O + 0.1% FA and MeOH + 0.1%FA).

[U-^13^C]salicortin, [U-^13^C]HCH-salicortin, and [U-^13^C]tremulacin were obtained from a methanolic extract of ^13^C-labeled material of *P. deltoides x trichocarpa* as described previously (Feistel et al. 2018). Homogenization of plant material and frass samples was carried out with a Bertin Minilys cell disruptor (Bertin Technologies, Montigny-le-Bretonneux, France). To purify [U-^13^C]salicortin, [U-^13^C]HCH-salicortin, and [U-^13^C]tremulacin, a MN π^2^-column (250 × 4.6 mm, 5 µm particle size) was used. To purify 6’-*O*-benzoylsalicortinol, a MN C18 HTec (250 × 10 mm, 5 μm particle size) was used. Detailed information on the purification procedures is given in the Supporting Information (SI). To recover the compounds from the fractions, solvents were evaporated using a Büchi rotary evaporator Rotavapor R-114 (Büchi Labortechnik, Flawil, Switzerland). Methanol (MeOH, LCMS grade) used for extraction and chromatographic separation was purchased from Merck KGaA (Darmstadt, Germany) and used without further purification. Water used for HPLC and HPLC-HR-ESI-MS separations was obtained from a Milli-Q Synthesis A10 purifier (Merck KGaA, Darmstadt, Germany). Acetonitrile (LCMS grade) and formic acid (eluent additive for LC-MS) used for HPLC-HR-ESI-MS analyses were purchased from Merck KGaA (Darmstadt, Germany). HR-X SPE cartridges (500 mg sorbent/6 mL volume and 200 mg sorbent/3 mL volume), folded paper filters (90 mm), and paper disc filters (MN 615 ¼, 125 mm) were purchased from Macherey-Nagel. Syringe filters (0.45 μm, PA) were purchased from Carl Roth GmbH (Karlsruhe, Germany). Salicin, catechol, saligenin, salicylic acid, and benzoic acid were purchased from Merck KGaA (Darmstadt, Germany). For centrifugation, an Eppendorf Centrifuge 5415 R (Eppendorf SE, Hamburg, Germany) was used.

### Plant Material

Hybrid trembling aspen (*Populus tremula x tremuloides*) and *P. deltoides x trichocarpa* were grown outdoors at the greenhouse facilities of the Max Planck Institute for Chemical Ecology in Jena, Germany.

### Insect Larvae and Frass Sampling

Larvae of Notodontidae poplar specialists were either taken from a continuous rearing (*C. vinula*) at the outdoor butterfly facility of the Max Planck Institute for Chemical Ecology in Jena, Germany, or accessed from eggs (*C. erminea*, *C. anachoreta*, *F. furcula*, *N. ziczac*, and *P. tremula*), provided by amateur entomologists. Authentic pictures of the caterpillars used in this study are provided in the Supporting Information (SI Fig. 54). To compare insect metabolism, all species were reared on *P. deltoides x trichocarpa* during their entire life cycles. Frass of at least five individuals per species was collected, dried *in vacuo*, and stored at -20 °C until further use. To examine salicortinoid metabolism, *C. vinula* was reared on hybrid trembling aspen (*Populus tremula x tremuloides*).

Three replicates of frass samples of each species were collected as follows. 50 mg of frass were extracted with MeOH (5 × 2 mL) using a Bertin Minilys cell disruptor equipped with 2 mL Precellys^®^ tubes loaded with ZrO_2_ beads (1.4 mm o.d.). For each extraction, the tube content was shaken at 5500 rpm for 60 s. Afterwards the tube was centrifuged for 10 min at 13,200 rpm/16,100 rcf. The supernatants were pooled and filtered using a MN HR-X SPE cartridge (200 mg/3 mL) to remove strongly lipophilic content and remaining particles. The filtrate was then evaporated using N_2_ gas and subsequently dried for 24 h *in vacuo*. The weight of all samples was determined by means of a balance, and solutions of each sample were prepared at a concentration of 1 mg/mL. The samples were then subjected to HPLC-HR-ESI-MS analysis in negative ionization mode using the following gradient: H_2_O (solvent A) and acetonitrile (solvent B), both solvents containing 0.1% (v/v) formic acid. The flow rate was set to 500 µL min^− 1^, beginning at 5% B and increasing to 95% B within 28 min. Then columns were rinsed for 10 min with 100% B and re-equilibrated to 5% B for 5 min. The metabolites were identified by retention time, main isotope peak, and most prominent adducts as given in the SI (Supporting Information - MS peaklist).

### Isolation of [U-^13^C]salicortin, [U-^13^C]HCH-salicortin and [U-^13^C]tremulacin

An extract of ^13^C-labeled *P. deltoides x trichocarpa* leaf material (2.19 g) was used to isolate salicortinoids for metabolic studies (Feistel et al. 2018). After reconstitution with MeOH, aliquots (146.19 mg ml^− 1^) were subjected to HPLC separation using chromatographic conditions as described previously (Feistel et al. 2018). Subsequently, the isolated compounds were purified for a second time to remove traces of impurities. The obtained yields were as follows: 48.2 mg of [U-^13^C]salicortin, 45.1 mg of [U-^13^C]HCH-salicortin, and 21.5 mg of [U-^13^C]tremulacin. For details of the re-purification and analytical data of the purified ^13^C-labeled compounds, see SI. The calculation of ^13^C-enrichment of the ^13^C-labeled plant metabolites was accomplished as described previously (Taubert et al. 2011). [U-^13^C]salicortin showed 82%, [U-^13^C]HCH-salicortin 77%, and [U-^13^C]tremulacin 79% total ^13^C-enrichment. Spectroscopic data for all compounds and detailed information about the ^13^C-enrichment calculation are shown in SI (SI Fig. 9–Fig. 36, SI Tables 4–9).

### Spontaneous Degradation of Salicortinoids at pH 7.8

To examine the spontaneous decomposition of salicortinoids, a stock solution of the respective compound (1 mg ml^− 1^ in MeOH) was prepared. An aliquot of 10 µL of this stock solution was diluted with 90 µL of phosphate buffered saline (PBS, pH 7.8). The pH value was chosen on the basis of previous publications and results from our own experiments to determine the midgut pH of *C. vinula* larvae (Feistel 2018). Decomposition of the salicortinoids was monitored every 30 min by means of HPLC-HR-ESI-MS analysis as described above. As a control, the first analysis was done immediately after the salicortinoid aliquot was mixed with PBS. The decomposition products were identified by retention time, main isotope peak and most prominent adducts as given in the SI (Supporting Information – MS peaklist).

### Isolation of 6’-*O*-benzoylsalicortinol (6)

Frass of *C. vinula* fed on *P. tremula x tremuloides* (20 g, dry weight) was crushed in a mortar and extracted with MeOH (5 × 100 mL) in an Erlenmeyer flask. After passing the combined extracts through filter paper, the filtrate was passed through a cartridge filled with HR-X sorbent (30 mL) to remove small particles and very lipophilic compounds, e.g. fatty acids and chlorophyll. The eluate was rotary-evaporated to yield the crude extract (2.92 g). This crude extract was reconstituted with MeOH, and 800 mg of HR-X sorbent was added to adsorb the extract completely. To separate the crude extract coarsely, a cartridge (60 mL) filled with HR-X resin (5.0 g) was equilibrated with MeOH (100 mL) and conditioned with H_2_O (100 mL). Afterwards, the HR-X resin loaded with the crude extract was applied on top of the cartridge, and a sintered PP filter disc was inserted to compress the bed. A stepwise gradient elution was applied using a binary solvent system (H_2_O (A)/ MeOH (B), 0% to 100% B in 10% steps). For each step, a volume of 100 mL of the respective solvent mixture was used, and fractions of 50 mL were collected. In total, 23 fractions were obtained, and an aliquot of each was subjected to HPLC-HR-ESI-MS analysis. All fractions were then evaporated to dryness, and their weight was determined (see SI Table 1). Based on results of the analysis, the fraction 90%-I was subjected to separation by MPLC (Biotage Isolera One). The MPLC gradient started with 0% (two cartridge volumes, CVs) of MeOH (solvent B) and increased during the elution of one CV to 10% B. Afterwards, B increased within 42 CVs to 70%. The column was purged with seven CVs of 100% B. Aliquots of the MPLC fractions were subjected to HPLC-HR-ESI-MS analysis. Dry weights of the MPLC fractions are listed in SI Table 2. MPLC fraction seven (F7), containing 6’-*O*-benzoylsalicortinol (**6**), was reconstituted with 300 µL MeOH (47.5 mg mL^− 1^) and separated by semi-preparative HPLC (32% MeOH in H_2_O, 70 min isocratic elution at 3.5 mL min^− 1^ flow). After each run, the column was purged for 10 min with 100% MeOH and equilibrated for 10 min at initial conditions. The fraction containing 6’-*O*-benzoylsalicortinol (rt 27.2 min) was evaporated using N_2_ gas. The structure of the isolated compound was confirmed by HPLC-HR-ESI-MS and NMR spectroscopy (see SI Figs. 1–8; Table 3). The peak for 6’-*O*-benzoylsalicortinol (**6**) appeared at R_t_ = 11.6 min in HPLC-HR-ESI-MS.

### Decomposition of 6’-*O*-benzoylsalicortinol (6) at pH 6 to pH 9

To determine the decomposition products of 6’-*O*-benzoylsalicortinol (**6**) at various pH values, a stock solution of 2 mg mL^− 1^ in acetonitril was prepared. PBS (150 µl) adjusted to pH 6, pH 7, pH 8, and pH 9, respectively, was pipetted into a HPLC vial equipped with a 200 µl insert; 7.5 µL of the 6’-*O*-benzoylsalicortinol (**6**) stock solution was mixed in using several pipette strokes. The experiments were carried out at room temperature (25 °C). Decomposition of the compounds was measured every 2 h by HPLC-HR-ESI-MS using the same method as described above. The decomposition products were identified by retention time, main isotope peak and most prominent adducts as given in the SI (Supporting Information – MS peaklist).

### *Cerura vinula* Gut Homogenate Incubation Experiments

*Cerura vinula* larvae (5th instar), raised on *P. deltoides x trichocarpa* leaves, were immobilized in a Falcon tube and kept at -20 °C for 15 min. A midabdominal leg was removed with scissors, and the emerging hemolymph was absorbed with a tissue. Afterwards the caterpillar was opened by a ventral cut. The midgut was separated from fore- and hindgut, and malphigian tubulae were removed from the gut tissue using a pair of tweezers. The midgut was emptied and extensively rinsed with PBS (pH 7.8) until no traces of gut content remained. During the dissection procedure, samples were stored on ice and kept at -80 °C until further use. For incubation experiments, the frozen midgut tissue was thawed on ice and manually homogenized in a Potter-Elvehjem tissue grinder with 1 mL chilled PBS (pH 7.8). The gut homogenate was divided into two 500 µL portions and transferred into 1.5 mL Eppendorf tubes. For the assay, 500 µL gut homogenate, 500 µL PBS (pH 7.8), and 10 µL salicortinoid stock solution in dimethylsulfoxide (DMSO, concentration 10 mg/mL) were mixed. To determine the background, 500 µL gut homogenate, 500 µL PBS (pH 7.8), and 10 µL DMSO was used. The incubation was carried out at room temperature (25 °C). For sampling, aliquots (100 µL) of both groups were taken initially after mixing and at 0.5 h, 1 h, 1.5 h, 2 h, 3 h, and 4 h. Prior to the analyses, the decomposition reaction was quenched by the addition of 0.1% formic acid in methanol (100 µL). Samples were then centrifuged at 4 °C for 10 min at 13,200 rpm/16,100 rcf. The supernatant was transferred into a HPLC vial and subjected to HPLC-HR-ESI-MS analysis using the same conditions as described above. The metabolites were identified by retention time, main isotope peak, and most prominent adducts as given in the SI (Supporting Information - MS peaklist).

## Results

### Salicortinoids Degrade Slowly in vitro at pH 7.8

**Fig. 2:**
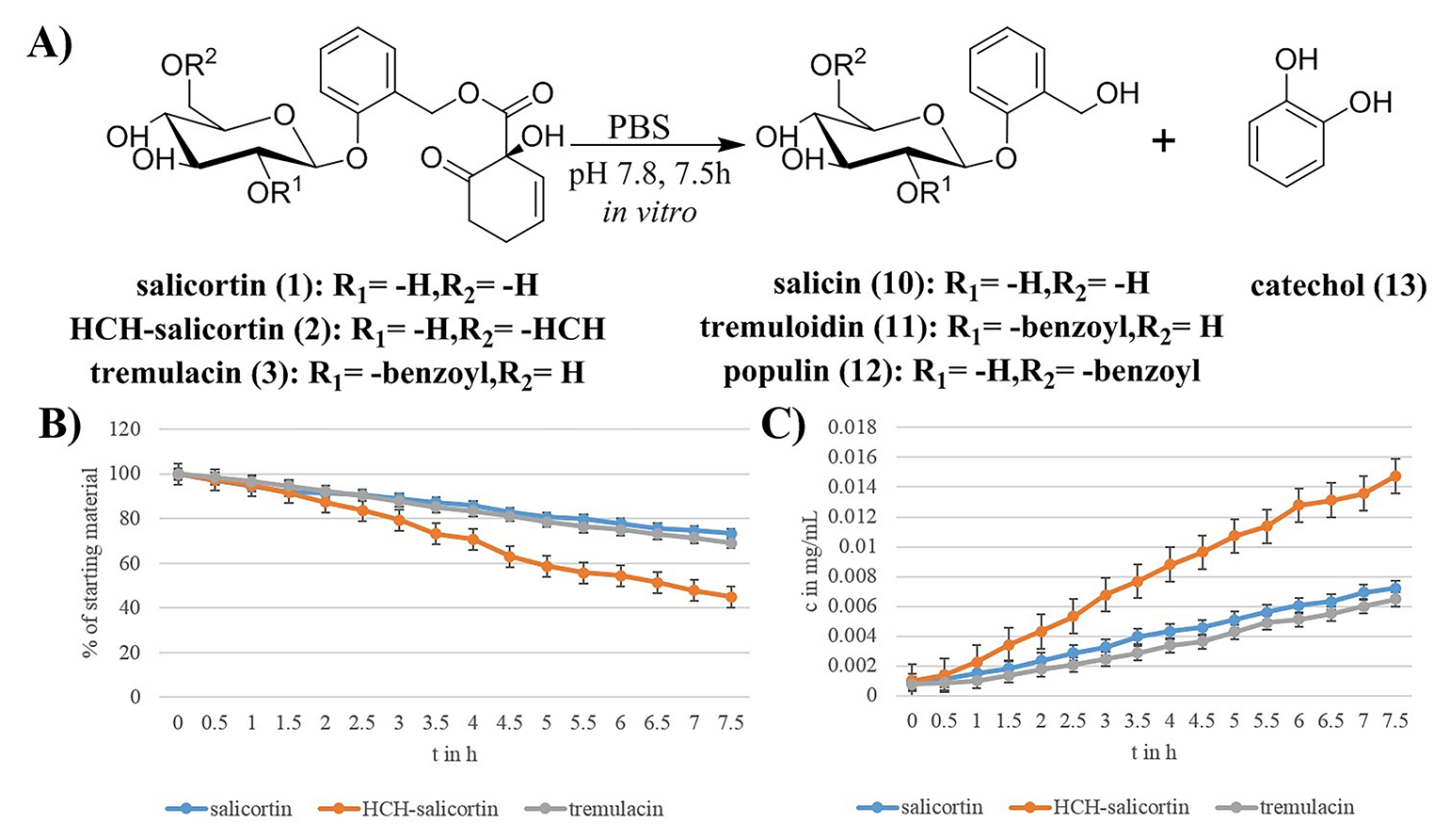
Results from the decomposition experiments of salicortinoids in PBS at pH 7.8. (**A**) salicortin (**1**) and HCH-salicortin (**2**) decompose to salicin (**10**) and catechol (**13**); tremulacin decomposes to tremuloidin (**11**), populin (**12**), and catechol (**13**). (**B**) Percentage of intact salicortinoids during decomposition experiments (pH 7.8). (**C**) Catechol (**13**) concentration during decomposition of salicortinoids (pH 7.8)

The midgut of most lepidopteran larvae has an alkaline pH value (Dow 1992; Harrison 2001). Chemical defense compounds like the salicortinoids are known to decompose quickly under alkaline conditions (Pearl and Darling 1971). We therefore wanted to estimate the contribution of spontaneously degraded compounds before analyzing the salicortinoid metabolites in gut homogenate experiments. To determine the chemical stability of salicortinoids at conditions present in the gut of *C. vinula* larvae, in vitro hydrolysis experiments were conducted. 100 µg of salicortinoid was incubated at 25 °C, and decomposition was monitored every 30 min by HPLC-HR-ESI-MS analysis. Hydrolysis products of salicortin (**1**) generated in vitro were determined to be salicin (**10**) and catechol (**13)** (Fig. 2A). For HCH-salicortin (**2**), the hydrolysis products were salicortin (**1**), salicin (**10**), and catechol (Fig. 2A). Salicortin (**1**), salicin (**10**), catechol, and also tremuloidin (**11**) and populin (**12**) were identified as degradation products of tremulacin, suggesting that an ester cleavage liberated the HCH-moiety, which was then oxidized to catechol (Fig. 2A, SI Fig. 37, and Fig. 38). Spontaneous salicortinoid decomposition was slow at pH 7.8. After 7.5 h of incubation, 73% of salicortin (**1**), 45% of HCH-salicortin (**2**), and 69% tremulacin remained (Fig. 2B). In comparison to salicortin (**1**), HCH-salicortin generated about twice as much catechol (Fig. 2C). This increase also indicated that the origin of catechol has to be the HCH-moiety in salicortinoids.

### Salicortinoids Degrade Quickly in Midgut Homogenate at pH 7.8

We assumed that the main metabolic steps happen in the midgut of *C. vinula* larvae and conducted experiments with midgut homogenate at pH 7.8 to follow the degradation of salicortinoids. To exclude any interference with transformations catalyzed by plant enzymes, the midgut tissue of *C. vinula* was rinsed extensively prior to the incubation experiments. We used ^13^C-labeled substrates to exclude interference with sequestered compounds in the gut tissue.

**Fig. 3:**
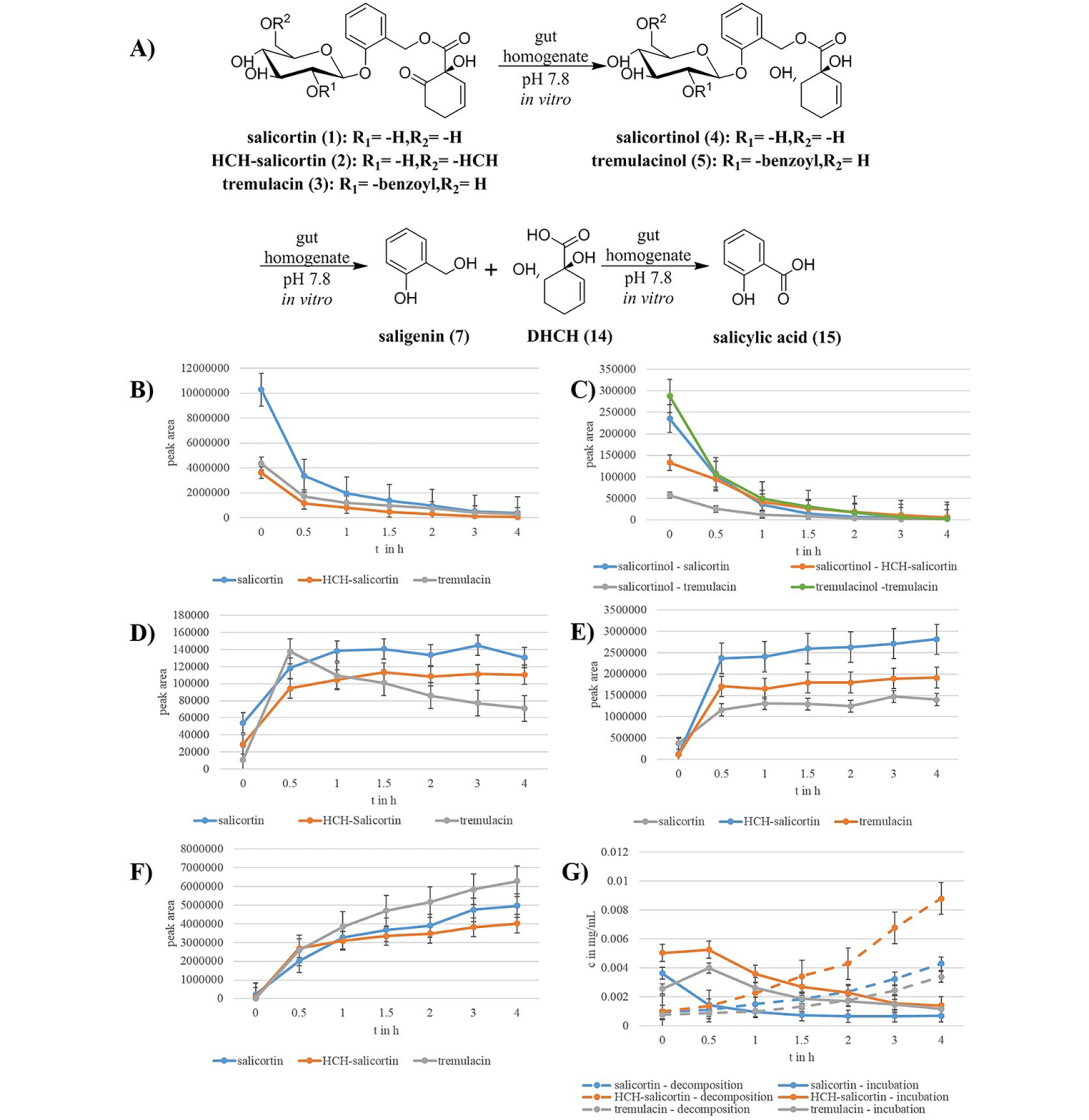
Results of incubation experiments with gut homogenate (pH 7.8), (**B)** to (**E)**: Concentrations of metabolites vs. time, (**A**) metabolic reactions observed during the gut homogenate incubation experiments. Salicortin (**1**) is reduced to salicortinol (**4**), the glycosidic 6-HCH-moiety of HCH-salicortin (**2**) is cleaved, while the benzylic 6-HCH-moiety is reduced to form salicortinol (**4**). Tremulacin (**3**) forms tremulacinol (**5**). **4** and **5** are further hydrolyzed to saligenin (**7**) and DHCH (**14**). (**B**) Decay of salicortinoids occurs during the incubation with gut homogenate. Salicortin (**1**) and HCH-salicortin (**2**) decay at the same rate. (**C**) Relative concentration of reduced salicortinoids during the experiments. (**D**) Relative concentration of saligenin (**7**) generated from salicortinoids. (**E**) Relative concentration of DHCH (**14**) generated by reduction and cleavage of the 6-HCH-moiety from salicortinoids. (**F**) Relative concentration of salicylic acid (**15**) generated by the oxidation of saligenin (**7**) and DHCH (**14**). (**G**) Concentration of catechol (**13**) from in vitro decomposition experiments with buffer pH 7.8 (dashed lines) vs. gut homogenate incubations (continuous lines)

We observed that all tested salicortinoids were completely degraded within four hours with a half-life time of approximately 30 min (Fig. 3B). Already during initial mixing of salicortinoids and gut homogenate, a reduction of the 6-HCH-moieties of the compounds took place and thus we observed salicortinol (**4**) and tremulacinol (**5**) (Fig. 3C). Both compounds were further converted at the same rate as salicortin (**1**) and tremulacin (**3**), respectively. For all salicortinoids, we observed ^13^C-labeled salicin (**10**), saligenin (**7**), and 1,6-dihydroxycyclohex-2-ene-1-carboxylic acid (hereafter referred to as DHCH (**14**)) as metabolites (Fig. 3A). For tremulacin, we detected short-lived benzoylated compounds such as tremuloidin (**11**) and populin (**12**) (see SI Figs. 40–50).

Saligenin (**7**) was formed from all salicortinoids, (Fig. 3D). The HCH-moiety of the salicortinoids was transformed into DHCH (**14**) (Fig. 3E). Saligenin and DHCH were oxidized to salicylic acid (**15**) (Fig. 3F). Salicylate adducts with quinic acid were found as abundant metabolites in Notodontidae larvae frass. Catechol (**13**) was generated in all gut homogenate incubation experiments but in lower concentrations compared to those determined for the in vitro degradation (Fig. 3G).

### The Tremulacinol Isomer 6’-*O*-benzoylsalicortinol (6) Results From Reductively Transformed Tremulacin

The incubation experiments with *C. vinula* gut homogenate revealed reductively transformed salicortinoids as intermediates of the metabolic degradation process. We wanted to understand the metabolization of these intermediates in detail and used the more stable form of tremulacinol, 6’-*O*-benzoylsalicortinol (**6**), as the object of our studies. The molecule is derived from tremulacinol (**5**) through acyl migration of the benzoyl substituent. When we re-examined the frass constituents of *C. vinula* after the larvae were raised on *P. tremula x tremuloides* leaves, we detected a compound with the molecular formula C_27_H_30_O_11_ (*m/z* 529.1728 [M-H]^−^, calc. for C_27_H_29_O_11_^−^, *m/z* 529.1715). The main fragment ion was determined at *m/z* 389.1267 [M-H]^−^, corresponding to a molecular formula of C_20_H_22_O_8_. The molecular composition suggested the presence of tremuloidin or populin. Another main fragment of this compound displayed a HRESIMS ion at *m/z* 157.0508 [M-H]^−^, indicating a sum formula of C_7_H_9_O_4_^−^ (calc. for C_7_H_9_O4^−^, *m/z* 157.0506). Analysis of the fragmentation pattern suggested a reductively transformed tremulacin-like compound. Using NMR spectroscopy, we identified the structure as 6’-*O*-benzoylsalicortinol (**6**), where the 6-HCH-moiety is reduced to 1,6-dihydroxycyclohex-2-ene-1-carboxylate. Comparison with literature data led us to define the stereochemistry as (*S*)-configured at C1’ and (*R*)-configured at C6’ (SI Figs. 1–8; Table 3) (Wei et al. 2015).

### The in vitro Decomposition of 6’-*O*-benzoylsalicortinol (6) is pH Dependent

**Fig. 4:**
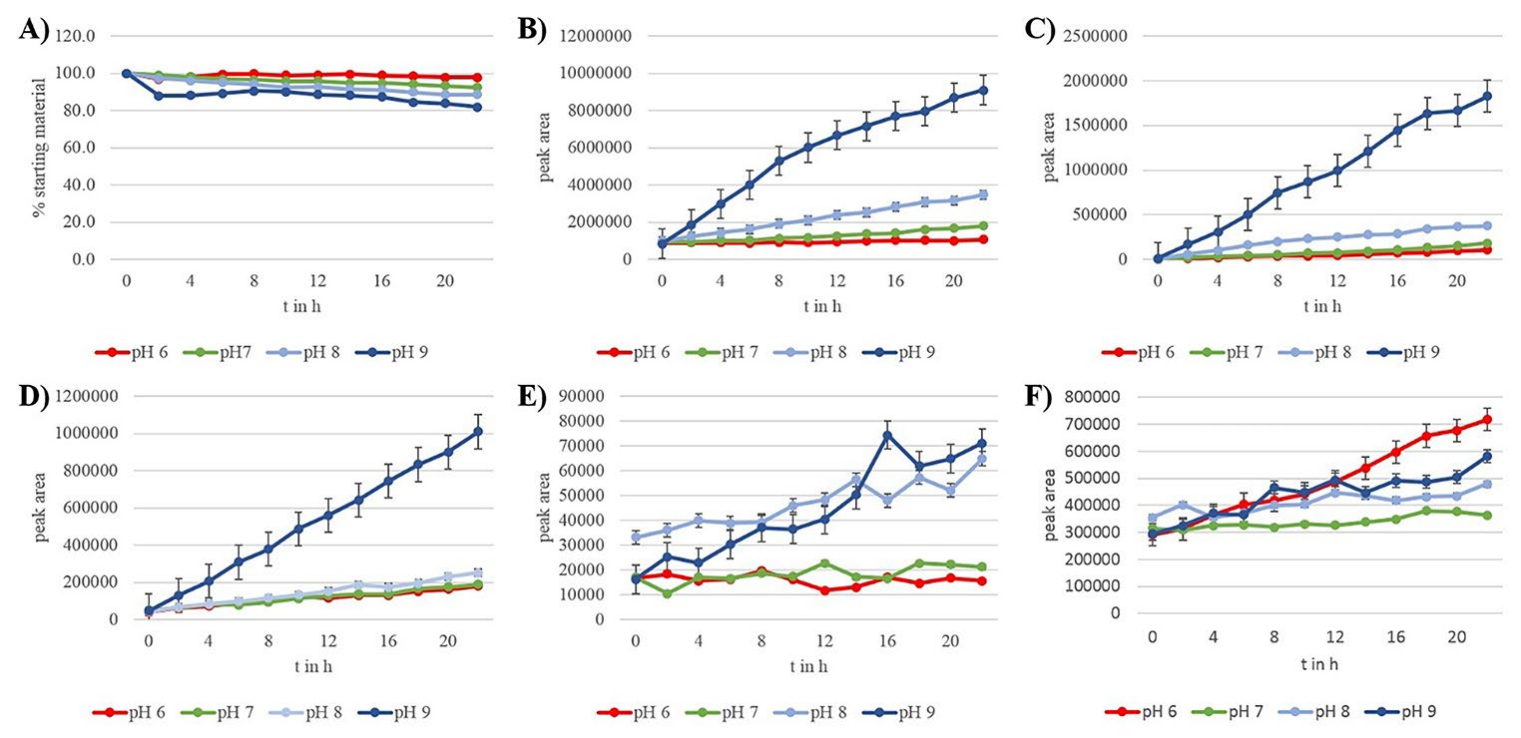
Results of in vitro degradation of 6’-*O*-benzoylsalicortinol. (**A**) Percentage of intact 6’-*O*-benzoyl-salicortinol (**6**) in various buffers as determined by UV-Vis. Peak area of populin (**12**) (**B**) and DHCH (**14**) (**C**) generated by benzylic ester cleavage from 6’-*O*-benzoylsalicortinol. (**D**) Peak area of salicortinol (**4**) generated by glucosidic ester cleavage of 6’-*O*-benzoylsalicortinol. (**E**) Peak area of salicin (**10**) generated by benzylic ester cleavage of populin. (**F**) Peak area of salicylic acid (**15**) generated by autoxidation of DHCH (**14**)

The gut of *C. vinula* is divided into zones of differential pH values. Whereas the fore- and hindguts are acidic (Feistel 2018), the midgut is alkaline (Dow 1992; Harrison 2001). We consequently investigated the metabolization of 6’-*O*-benzoylsalicortinol (**6**) over a broad pH range, from alkaline (pH 9) to acidic (pH 6). An equimolar amount of the compound was dissolved in PBS adjusted to pH 6, pH 7, pH 8, and pH 9, and its decomposition products were analyzed by HPLC-HR-ESI-MS. Samples were taken every 2 h over a time course of 22 h. Only 2% (pH 6) to 18% (pH 9) of the compound decomposed, and we identified the same decomposition products for all tested pH values: salicortinol (**4**), populin (**12**), salicin (**10**), DHCH (**14**), and salicylic acid (**15**). Remarkably, no catechol formation was observed. The highest amounts of salicortinol (**4**), populin (**12**), salicin (**10**), and DHCH (**14**) were observed at pH 9. The decomposition at pH 8 occurred more slowly than that at pH9, and the slowest decomposition was observed at pH 6. The highest amount of salicylic acid (**15**) was formed at pH 6, the second highest amount of salicylic acid (**15**) at pH 9, and the lowest at pH 7. Based on these observations, we assume that salicylic acid is formed under either alkaline or acidic conditions, noting that acidic conditions catalyzed the DHCH (**14**) conversion to salicylic acid (**15**) most efficiently (Fig. 4).

### The Salicortinoid Metabolism is Similar in Notodontidae Specialists

Only *C. vinula’s* metabolism of its host plant’s defensive compounds (out of all Notodontidae) was examined to date. We asked whether our results were valid for other species of the Notodontidae. Consequently, we compared the frass metabolites of other specialist herbivores adapted to thrive on Salicaceae.

All Notodontidae larvae performed well on their diet (*P. deltoides x trichocarpa* foliage). We prepared samples by extracting frass with MeOH and subjected them to analysis by HPLC-HR-ESI-MS. Metabolites are composed of highly similar compounds (Table 1). All investigated species excreted salicin (**10**), salicylic acid (**15**), and DHCH (**14**). Tremulacinol (**5**) was found for all species, with an exception for *F. furcula*. The various salicyloyl- and benzoyl-quinic acid esters were also present in all investigated species. Based on these findings, we conclude that the metabolism of Salicaceae compounds in Notodontidae proceeds by the same enzymatic transformations.

**Table 1:** Main salicinoid and salicortinoid metabolites in frass of Notodontidae specialist larvae raised on *P. deltoides x trichocarpa*. Frass extracts were analyzed by HPLC-HR-ESI-MS. S, salicin (**10**); SA, salicylic acid (**15**); T, tremulacinol (**5**); SA-QA, mono-salicyloyl-quinic acid ester; BA-QA, mono-benzoyl-quinic acid ester; 2SA-QA, bis-salicyloyl-quinic acid ester; SB-QA, salicyloyl-benzoyl-quinic acid; detected, +; not detected, -

Species	S	SA	DHCH	T	SA-QA	BA-QA	2SA-QA	SB-QA
*Cerura vinula*	+	+	+	+	+	+	+	+
*Cerura erminea*	+	+	+	+	+	+	+	+
***Furcula furcula***	**+**	**+**	**+**	**-**	**+**	**+**	**+**	**+**
***Clostera anachoreta***	**+**	**+**	**+**	**+**	**+**	**+**	**+**	**+**
***Notodonta ziczac***	**+**	**+**	**+**	**+**	**+**	**+**	**+**	**+**
***Pheosia tremula***	**+**	**+**	**+**	**+**	**+**	**+**	**+**	**+**

## Discussion

Earlier in vitro studies of the metabolism of salicortinoids assumed that degradation by *β*-glucosidases and esterases leads to the release of saligenin and the 6-HCH-moiety (Julkunen-Tiitto and Meier 1992; Lindroth 1988). The latter is further oxidized to catechol and/or *ortho*-quinone under the alkaline conditions present in lepidopteran midgut (Appel 1993; Appel and Martin 1990; Harrison 2001). *Ortho*-quinone is believed to cause harm to herbivores by protein cross-linking (Felton et al. 1992; Haruta et al. 2001). Recent research on *C. vinula* established another pathway for the metabolism of salicortinoids, based on observations that larvae fed with [U-^13^ C]salicortin excreted salicylic acid conjugates. This led to the conclusion that both parts of salicortin, saligenin and the 6-HCH-moiety, were transformed to salicylic acid (Feistel et al. 2018). However, the mechanism of this transformation remained unclear. For the present study, we aimed to understand the mechanism of metabolic salicortinoid degradation and, to that end, conducted experiments with salicortin, HCH-salicortin, and tremulacin.

Although we assumed that salicortinoids undergo enzymatic transformation (likely the major degradation mechanism), we hypothesized that salicortinoids might also degrade spontaneously. Therefore we compared the stability of salicortin, HCH-salicortin, and tremulacin in vitro at pH conditions present in the midgut of *C. vinula* (pH 7.8). The salicortinoids were observed to quickly decompose under strongly alkaline conditions (Pearl and Darling 1971), but in our experiments, at certain physiological conditions (pH 7.8), the main part of the compounds remained intact until the end of the experiment (for 7.5 h). Salicortin hydrolyzed to salicin and catechol, and HCH-salicortin to salicortin, salicin, and catechol. As expected, HCH-salicortin released twice as much catechol as salicortin. This is in accordance with earlier results that identified the HCH-moiety in salicortinoids as the source of catechol under alkaline conditions (Boeckler et al. 2016; Julkunen-Tiitto and Meier 1992; Ruuhola et al. 2003). Decomposition products of tremulacin were catechol, salicortin, salicin, tremuloidin, and populin. In tremuloidin, we observed acyl-migration from the 2’-OH group to the 6’-OH group of the glucosyl part (Pearl and Darling 1963). In summary, we found that the midgut conditions (pH of 7.8) hindered the spontaneous degradation of salicortinoids.

We then wanted to follow the enzymatic degradation process of salicortinoids in *C. vinula*. Several studies have used midgut homogenate or enzyme preparations to elucidate the metabolism of plant xenobiotics (Lindroth 1988; Marty and Krieger 1984; Wouters et al. 2014). In our study, when samples were incubated with midgut homogenate at pH 7.8, the tested salicortinoids degraded completely within 4 h. We therefore concluded that degradation was the result of enzymatic action. Although we cannot exclude the involvement of microorganisms residing in the gut tissue, recent findings point to a minor role of microorganisms in caterpillars (Hammer et al. 2017).

**Fig. 5:**
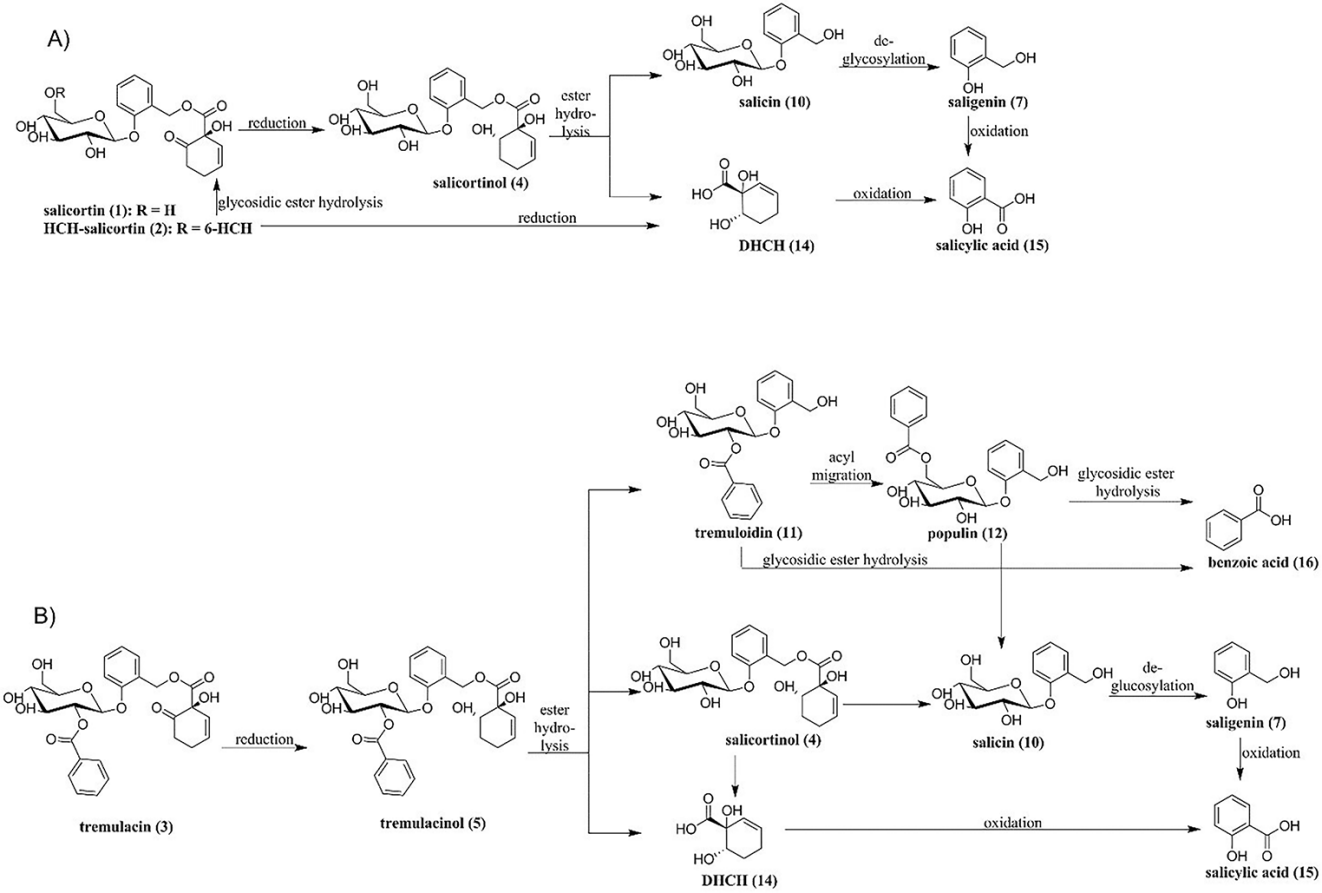
Overview of the transformations of salicortinoids by *C. vinula* midgut homogenate

For salicortin, we tentatively identified the metabolic breakdown products as salicortinol, salicin, saligenin, DHCH, salicylic acid, and catechol. Salicortinol, a reduced metabolite of salicortin, was hydrolyzed to salicin and DHCH. We also found the metabolism of HCH-salicortin to be highly similar to that of salicortin (Fig. 5A). Although we did not find reduced forms of HCH-salicortin, we observed the formation of a compound we assumed to be salicortinol. Both HCH-moieties of HCH-salicortin seemed to be reduced, since the signal intensity of free DHCH is highest for HCH-salicortin (Fig. 3E). The additional HCH-moiety, which is initially cleaved from the glucosylated form, resulting in salicortin, then undergoes the breakdown described above. The metabolism of tremulacin (Fig. 5B) is similar to that of salicortin. In the initial step, a reduced metabolite of tremulacin is formed that we attributed to tremulacinol. After tremulacinol was hydrolyzed, we observed salicortinol, as well as tremuloidin, populin, and DHCH. Salicortinol and DHCH were metabolized as described above. We observed the migration of the benzoyl moiety of tremuloidin to position 6’-OH and also an ester cleavage. The resulting salicin was then transformed as described for salicortin. We summarized the metabolic reactions in Fig. 5.

The formation of catechol was observed for all tested salicortinoids when samples were incubated with the midgut homogenate. Interestingly, the compound was only formed initially; during the course of the experiment, its concentration decreased, probably due to protein binding (Felton et al. 1992). The catechol that formed initially is attributable to the uncontrolled action of glucosidases and esterases, which was described in other in vitro studies (Julkunen-Tiitto and Meier 1992). Notably, the concentration of catechol never reached that observed in the buffer-only decomposition experiments, although more than 50% of the salicortinoids had degraded within the first 30 min. Instead of being oxidized, 6-HCH was reduced, as evident from the high amount of free DHCH (Fig. 3E). Whether an insect will benefit from the suppression of all catechol is questionable. Previous studies have shown that the oxidized form of catechol, *ortho*-quinone, can bind to the occlusion bodies of the nuclear polyhedrosis virus (Felton and Duffey 1990). This binding reduces the infectivity of the virus and improves larval survival rates after a viral challenge (Ali et al. 1999; Wan et al. 2018; Wang et al. 2020).

The HPLC-HR-ESI-MS data suggested that reduced salicortinoids occur during the metabolism. It was not possible to isolate these metabolites when we followed the breakdown of salicortin and HCH-salicortin. We could, however, observe that a more stable reductively transformed product was formed during the breakdown of tremulacin. To be certain that reduction is indeed an essential part of the salicortinoid breakdown in *C. vinula*, we attempted to isolate this metabolite from frass.

**Fig. 6:**
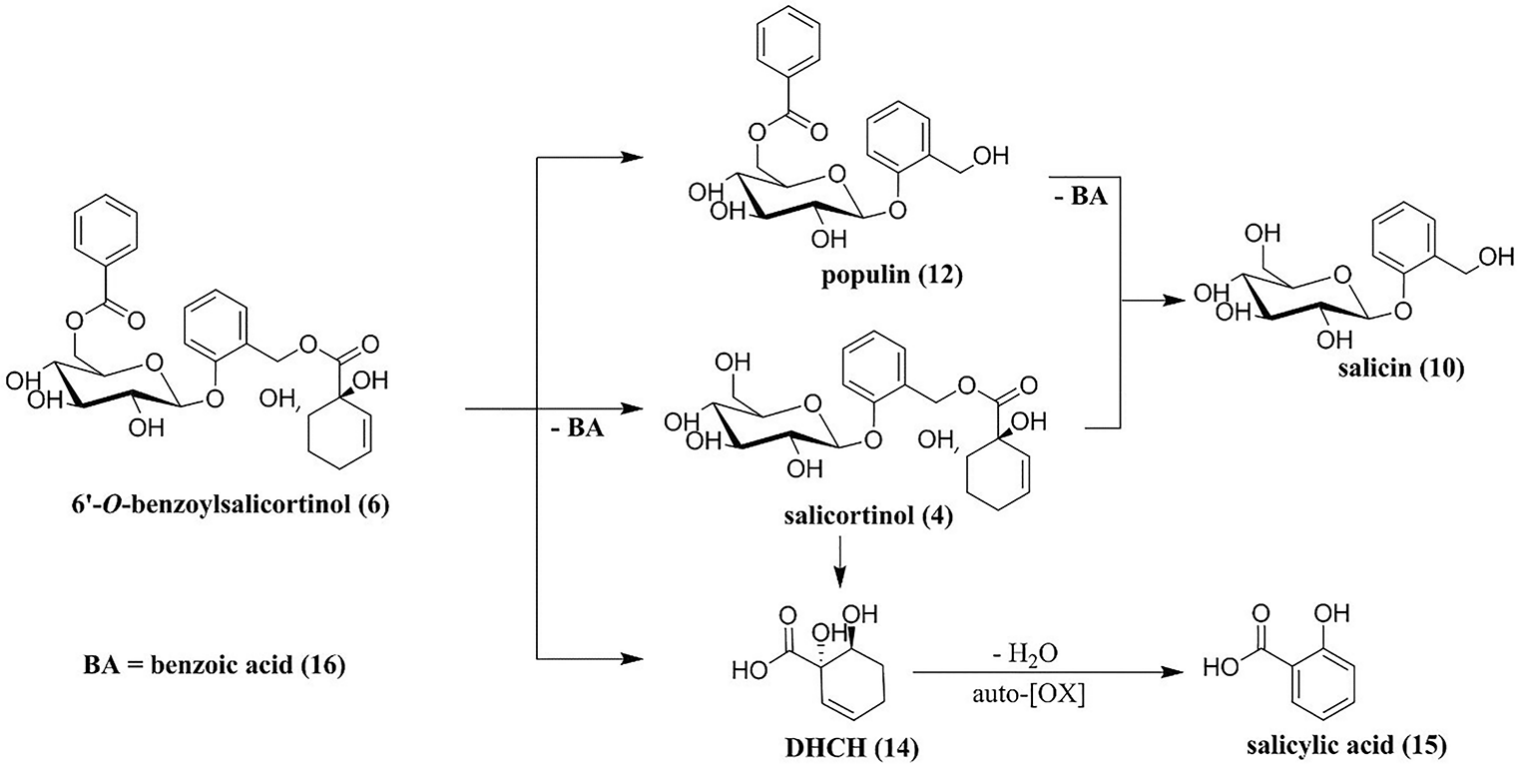
Decomposition of 6’-*O*-benzoylsalicortinol in PBS at pH 6 to pH 9

For this, we raised *C. vinula* larvae on *P. tremula x tremuloides*, a species whose leaves contain high amounts of the salicortinoids tremulacin and salicortin (see SI Fig. 50). After isolating an intermediate product that resulted from a reductive transformation of tremulacin by means of chromatography, we elucidated its structure by NMR spectroscopy. The metabolite was revealed to be 6’-*O*-benzoylsalicortinol, a rearranged derivative of tremulacinol, where the 2’-benzoyl group migrated to position 6’. The isolated 6’-*O*-benzoylsalicortinol was incubated at 25 °C in PBS at pH 6, pH 7, pH 8, and pH 9 to test its stability and to identify possible decomposition products. Only 2% of the compound decomposed at pH 6, while 18% decomposed at pH 9 during an experimental time of 22 h. The decomposition products, results of ester hydrolyses, were elucidated as populin, salicortinol, salicin, and DHCH (Ruuhola et al. 2003). We also identified salicylic acid as a breakdown product (Fig. 6). Catalyzed by alkaline conditions, ester cleavage illustrates the relationship between pH values and speed of degradation: the higher its pH, the faster a compound degrades.

For the salicortinoids salicortin, HCH-salicortin and tremulacin, we observed the formation of catechol during the incubation with buffer solutions of different pH values. Interestingly, regardless of the pH, no catechol formation was observed when 6’-*O*-benzoylsalicortinol was incubated. We conclude that the reductive transformation effectively hinders the production of toxic catechol and therefore this transformation represents a true detoxification step in the metabolism of salicortinoids.

After we found the key transformation of the salicortinoid metabolism for *C. vinula*, we wanted to know if this mechanism is a common feature in other Notodontidae species. We raised five other Salicaceae specialists, larvae of *Cerura erminea*, *Clostera anachoreta*, *Furcula furcula*, *Notodonta ziczac*, and *Pheosia tremula*, on *P. deltoides x trichocarpa*, and found that all of them excreted salicyloyl- and benzoyl quinc acid esters like *C. vinula*. Also the reductive transformation of salicortinoids could be observed in all species. *Clostera anachoreta* was reported to be a major pest in poplar plantations (Liang et al. 2006). Insights into the adaptation of these species will improve our understanding of the ecology of Notodontidae and their host plants.

In this study, we identified the metabolic key transformation that initiates the detoxification in Notodontidae, a previously unknown deactivation mechanism for salicortinoids. After observing this mechanism in all examined Notodontidae caterpillars, we conclude that this transformation represents an important adaption to the salicortinoid defense system of *Populus spp*. We believe that a similar mechanism for deactivating salicortinoids also evolved in other Salicaceae specialists.

## Electronic Supplementary Material

Below is the link to the electronic supplementary material.

Supplementary Material 1
