# Supplementary material for: Reductive Conversion Leads to Detoxification of Salicortin-like Chemical Defenses (Salicortinoids) in Lepidopteran Specialist Herbivores (Notodontidae)
Source: J Chem Ecol. 2023 May 16;49(5-6):251–61. doi: 10.1007/s10886-023-01423-4 (PMC10495269; doi:10.1007/s10886-023-01423-4)
Supplement: Supplementary file 1 — Supplementary Material 1 [file 10886_2023_1423_MOESM1_ESM.docx]

**Supporting Information**

**On the Metabolic Conversion of Salicortin-like Chemical Defenses (Salicortinoids) in the Lepidopteran Specialist Herbivore *Cerura vinula* (Notodontidae)**

Florian Schnurrer, Christian Paetz^*^

*Max-Planck-Institute for Chemical Ecology, Hans-Knöll-Straße 8, 07745 Jena, Germany*

Table of Contents

[Extraction of 6’-*O*-benzoyl-salicortinol 4](#_Toc120277492)

[Structure of 6’-*O*-benzoyl-salicortinol 5](#_Toc120277493)

[Isolation of [U-^13^C]salicortin, [U-^13^C]HCH-salicortin and [U-^13^C]tremulacin 10](#_Toc120277494)

[Characterization of isolated ^13^C-labelled compounds 11](#_Toc120277495)

[[U-^13^C]salicortin 11](#_Toc120277496)

[[U-^13^C]HCH-salicortin 17](#_Toc120277497)

[[U-^13^C]Tremulacin 23](#_Toc120277498)

[Decomposition of salicortinoids at pH 7.8 30](#_Toc120277499)

[Gut homogenate incubation experiments 31](#_Toc120277500)

[HPLC-HR-ESI-MS Chromatogram of the methanolic extract of *P. tremula x tremuloides* leaves 36](#_Toc120277501)

[HPLC-HR-ESI-MS Chromatogram of the methanolic extract of *C. vinula* frass after feeding on *P. tremula x tremuloides* leaves 36](#_Toc120277502)

[Mechanism of DHCH transformation to salicylic acid 37](#_Toc120277503)

[**Table 1**: Masses of fractions from coarse frass separation on HR-X resin 4](#_Toc120878729)

[**Table 2**: Masses of MPLC fractions after drying (Biotage Isolera One/Biotage Sfär C18 Duo column) 4](#_Toc120878730)

[**Table 3**: Mass fragments of 6'-*O*-benzoyl-salicortinol 9](#_Toc120878731)

[**Table 4**: Extracted MS data used for the calculation of the ^13^C-enrichment of salicortin isotopologues (m/z) together with their signal intensities 11](#_Toc120878732)

[**Table 5**: Extracted MS data used for the calculation of the ^13^C-enrichment of the salicortin isotopologues (m/z) together with their signal intensities 12](#_Toc120878733)

[**Table 6**: Extracted MS data used for calculation of the ^13^C-enrichment of the HCH-salicortin isotopologues (m/z) together with their signal intensities 17](#_Toc120878734)

[**Table 7**: Extracted MS data used for the calculation of ^13^C-enrichment of HCH-salicortin isotopologues (m/z) together with their signal intensities 18](#_Toc120878735)

[**Tab. 8**: Extracted MS data used for the calculation of ^13^C-enrichment of tremulacin isotopologues (m/z) together with their signal intensities 24](#_Toc120878736)

[**Tab. 9**: Extracted MS data used for calculation of ^13^C-enrichment of the tremulacin isotopologues (m/z) together with their signal intensities 25](#_Toc120878737)

[**Fig. 1**: Structure of 6’-*O*-benzoyl-salicortinol as determined by NMR-spectroscopy. Chemical shift δ_H_ (mult., J_HH_) in red, δ_C_ in blue. 6](#_Toc130477813)

[**Fig. 2**: 6’-*O*-benzoyl-salicortinol, 1H-NMR spectrum (MeOH-*d*3) 7](#_Toc130477814)

[**Fig. 3**: 6’-*O*-benzoyl-salicortinol, ^1^H-^13^C HSQC spectrum (MeOH-*d*_3_) 7](#_Toc130477815)

[**Fig. 4**: 6’-*O*-benzoyl-salicortinol, ^1^H-^13^C HMBC spectrum (MeOH-*d*_3_) 8](#_Toc130477816)

[**Fig. 5**: 6’-*O*-benzoyl-salicortinol, ^1^H-^1^H COSY spectrum (MeOH-*d*_3_) 8](#_Toc130477817)

[**Fig. 6**: 6’-*O*-benzoyl-salicortinol, ^1^H-^1^H TOCSY spectrum (MeOH-*d*_3_) 9](#_Toc130477818)

[**Fig. 7**: HR-ESI-MS^2^ spectrum of 6'-*O*-benzoyl-salicortinol 9](#_Toc130477819)

[**Fig. 8**: HR-ESI-MS^2^ spectrum of 6'-*O*-benzoyl-salicortinol, main isotope peak 10](#_Toc130477820)

[**Fig. 9**: [U-^13^C]salicortin, HR-ESI-MS spectrum, m/z 423.1308 [M-H]^-^ 12](#_Toc130477821)

[**Fig. 10**: [U-^13^C]salicortin, structure with chemical shifts (MeOH-*d*_3_) 14](#_Toc130477822)

[**Fig. 11**: [U-^13^C]salicortin, ^1^H-NMR spectrum (MeOH-*d*_3_) 14](#_Toc130477823)

[**Fig. 12**: [U-^13^C]salicortin, ^1^H-NMR spectrum, ^13^C-decoupled (MeOH-*d*_3_) 15](#_Toc130477824)

[**Fig. 13**: [U-^13^C]salicortin, ^13^C-NMR spectrum (MeOH-*d*_3_) 15](#_Toc130477825)

[**Fig. 14**: [U-^13^C]salicortin, ^1^H-^13^C HSQC spectrum (MeOH-*d*_3_) 16](#_Toc130477826)

[**Fig. 15**: [U-^13^C]salicortin, ^1^H-^13^C HMBC spectrum (MeOH-*d*_3_) 16](#_Toc130477827)

[**Fig. 16**: [U-^13^C]salicortin, ^13^C-^13^C COSY spectrum (MeOH-*d*_3_) 17](#_Toc130477828)

[**Fig. 17**: [U-^13^C]salicortin, UV spectrum from HPLC-DAD 17](#_Toc130477829)

[**Fig. 18**: [U-^13^C]HCH-salicortin, HRESIMS spectrum, m/z 561.1607 [M-H]^-^ 18](#_Toc130477830)

[**Fig. 19**: [U-^13^C]HCH-salicortin, chemical structure with chemical shifts (MeOH-*d*_3_) 20](#_Toc130477831)

[**Fig. 20**: [U-^13^C]HCH-salicortin, ^1^H-NMR spectrum (MeOH-*d*_3_) 20](#_Toc130477832)

[**Fig. 21**: [U-^13^C]HCH-salicortin, ^1^H-NMR spectrum, ^13^C-decoupled (MeOH-*d*_3_) 21](#_Toc130477833)

[**Fig. 22**: [U-^13^C]HCH-salicortin, ^13^C-NMR spectrum (MeOH-*d*_3_) 21](#_Toc130477834)

[**Fig. 23**: [U-^13^C]HCH-salicortin, ^1^H-^13^C HSQC spectrum (MeOH-*d*_3_) 22](#_Toc130477835)

[**Fig. 24**: [U-^13^C]HCH-salicortin, ^1^H-^13^C HMBC spectrum (MeOH-*d*_3_) 23](#_Toc130477836)

[**Fig. 25**: [U-^13^C]HCH-salicortin, ^13^C-^13^C COSY spectrum (MeOH-*d*_3_) 23](#_Toc130477837)

[**Fig. 26**: [U-^13^C]HCH-salicortin, UV spectrum from HPLC-DAD 24](#_Toc130477838)

[**Fig. 27**: [U-^13^C]tremulacin, HR-ESI-MS spectrum, m/z 527.1572 [M-H]^-^ 24](#_Toc130477839)

[**Fig. 28**: [U-^13^C]tremulacin, chemical structure with chemical shifts (MeOH-*d*_3_) 27](#_Toc130477840)

[**Fig. 29**: [U-^13^C]tremulacin, ^1^H-NMR spectrum (MeOH-*d*_3_) 27](#_Toc130477841)

[**Fig. 30**: [U-^13^C]tremulacin, ^1^H-NMR spectrum, ^13^C-decoupled (MeOH-*d*_3_) 28](#_Toc130477842)

[**Fig. 31**: [U-^13^C]tremulacin, ^13^C-NMR spectrum (MeOH-*d*_3_) 28](#_Toc130477843)

[**Fig. 32**: [U-^13^C]tremulacin, ^1^H-^13^C HSQC spectrum (MeOH-*d*_3_) 29](#_Toc130477844)

[**Fig. 33**: [U-^13^C]tremulacin, ^1^H-^13^C HMBC spectrum (MeOH-*d*_3_) 29](#_Toc130477845)

[**Fig. 34**: [U-^13^C]tremulacin, ^1^H-^1^H COSY spectrum (MeOH-*d*_3_) 30](#_Toc130477846)

[**Fig. 35**: [U-^13^C]tremulacin, ^13^C-^13^C COSY spectrum (MeOH-*d*_3_) 30](#_Toc130477847)

[**Fig. 36**: [U-^13^C] tremulacin, UV spectrum from HPLC-DAD 31](#_Toc130477848)

[**Fig. 37**: Peak area of salicin generated by benzylic ester cleavage of salicortin HCH-salicortin (by benzylic ester cleavage), and tremulacin (by benzylic and glycosidic ester cleavage) during decomposition experiments (pH 7.8) 31](#_Toc130477849)

[**Fig. 38**: peak area of tremuloidin and populin liberated by benzylic ester cleavage from tremulacin during decomposition experiments (pH 7.8) 32](#_Toc130477850)

[**Fig. 39**: Catechol concentration calibration. The concentration of catechol was calculated as follows: From the determination of salicortin decomposition we calculated the amount of intact salicortin based on UV data. From the difference of decomposed salicortin to intact salicortin we deduced the concentration of catechol formed. This value was correlated with the integral value of the ^12^C signal in the mass spectrum for catechol. 32](#_Toc130477851)

[**Fig. 40**: Labelling grade of salicortin (**1**) during gut homogentate incubation 33](#_Toc130477852)

[**Fig. 41**: Labelling grade of salicortinol (**4**) during gut homogenate incubation 33](#_Toc130477853)

[**Fig. 42**: Labelling grade of DHCH (**14**) during gut homogenate incubation 34](#_Toc130477854)

[**Fig. 43**: Labelling grade of salicin (**10**) during gut homogenate incubation 34](#_Toc130477855)

[**Fig. 44**: Labelling grade of saligenin (**7**) during gut homogenate incubation 35](#_Toc130477856)

[**Fig. 45**: Labelling grade of salicylic acid (**15**) during gut homogenate incubation 35](#_Toc130477857)

[**Fig. 46**: Labelling grade of catechol (**13**) during gut homogenate incubation 36](#_Toc130477858)

[**Fig. 47**: Labelling grade of tremulacinol (**5**) during gut homogenate incubation 36](#_Toc130477859)

[**Fig. 48**: Labelling grade of tremuloidin (**11**) during gut homogenate incubation 36](#_Toc130477860)

[**Fig. 49**: Labelling grade of populin (**12**) during gut homogenate incubation 37](#_Toc130477861)

[**Fig. 50**: peak area of populin (blue) and tremuloidin (orange) during gut homogenate incubation experiment 37](#_Toc130477862)

[**Fig. 51**: **A**: base peak chromatogram of *P. tremula x tremuloides* MeOH leaf extract, c = 1 mg/mL. **B**: Extracted ion chromatogram (m/z = 529.1715, calc. mass for tremulacinol and 6’-*O*-benzoyl-salicortinol) of *P. tremula x tremuloides* MeOH leaf extract 38](#_Toc130477863)

[**Fig. 52**: **A**: Base peak chromatogram of C. vinula (5^th^ instar) MeOH feces extract after feeding on P. tremula x tremuloides foliage, c = 1 mg/mL. **B**: Extracted ion chromatogram (m/z = 529.1715, calc. mass for tremulacinol and 6’-*O*-benzoyl-salicortinol) of C. vinula (5^th^ instar) MeOH feces extract after feeding on *P. tremula x tremuloides* foliage 38](#_Toc130477864)

[**Fig. 53**: Dehydration and subsequent auto-oxidation of DHCH to salicylic acid under acidic conditions and to salicylate under alkaline conditions. In the first step, the hydroxy group at position one is protonated and subsequently dehydrated. The resulting intermediate, here represented as tautomeric structures, is further converted by auto-oxidation to form salicylic acid. Under alkaline conditions, the first step is the dissociation of the carboxylic acid. Due to the close proximity of the 2-OH group, a hydrogen bond between the carboxylate ion and the alcohol function can be formed, stabilizing the deprotonated carboxyl function. In the next step, the 1-OH group is protonated and the molecule undergoes dehydration. The resulting intermediate is then auto-oxidized to salicylate. Salicylic acid formation from DHCH is fastest in acidic media. 39](#_Toc130477865)

[**Fig. 54:** Notodontidae species used in this study, authentic pictures 40](#_Toc130477866)

# Extraction of 6’-*O*-benzoyl-salicortinol

**Table 1**: Masses of fractions from coarse frass separation on HR-X resin

| % solvent B (MeOH) | mass in mg |
| --- | --- |
| 0% - I | 260.29 |
| 0% - II | 123.71 |
| 10% - I | 148.29 |
| 10% - II | 153.23 |
| 20% - I | 128.65 |
| 20% - II | 298.54 |
| 30% - I | 155.08 |
| 30% - II | 199.24 |
| 40% - I | 63.75 |
| 40% - II | 95.4 |
| 50% - I | 127.12 |
| 50% - II | 154.75 |
| 60% - I | 34.49 |
| 60% - II | 78.61 |
| 70% - I | 77.6 |
| 70% - II | 99.56 |
| 80% - I | 96.66 |
| 80% - II | 151.1 |
| 90% - I | 104.22 |
| 90% - II | 104.57 |
| 100% - I | 48.37 |
| 100% - II | 44.83 |
| 100% - III | 47.48 |

**Table 2**: Masses of MPLC fractions after drying (Biotage Isolera One/Biotage Sfär C18 Duo column)

| Fraction | mass in mg |
| --- | --- |
| F1 | 0.4 |
| F2 | 1.0 |
| F3 | 1.4 |
| F4 | 3.0 |
| F5 | 13.3 |
| F6 | 39.9 |
| F7 | 14.3 |
| F8 | 5.8 |

# Structure of 6’-*O*-benzoyl-salicortinol

**Fig. 1**: Structure of 6’-O-benzoyl-salicortinol as determined by NMR-spectroscopy. Chemical shift δ_H_ (mult., J_HH_) in red, δ_C_ in blue.


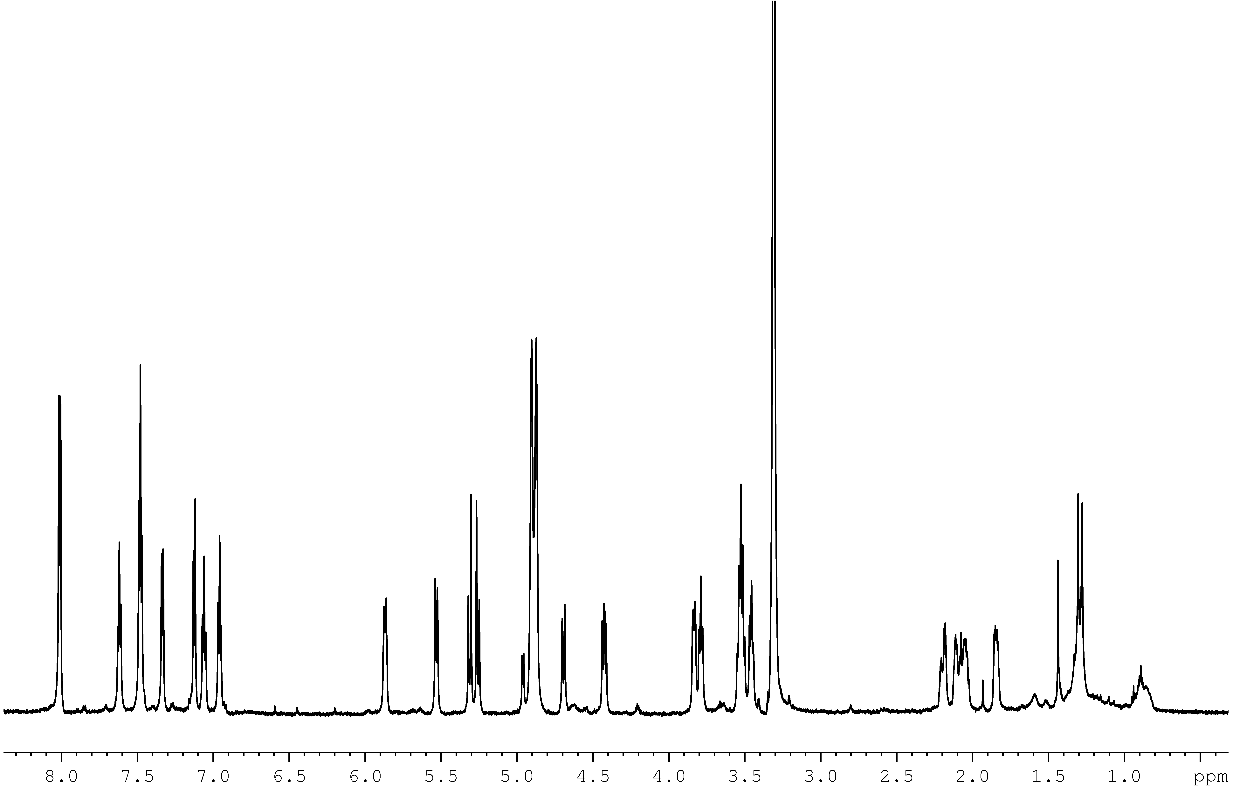


**Fig. 2**: 6’-O-benzoyl-salicortinol, ^1^H-NMR spectrum (MeOH-d3)


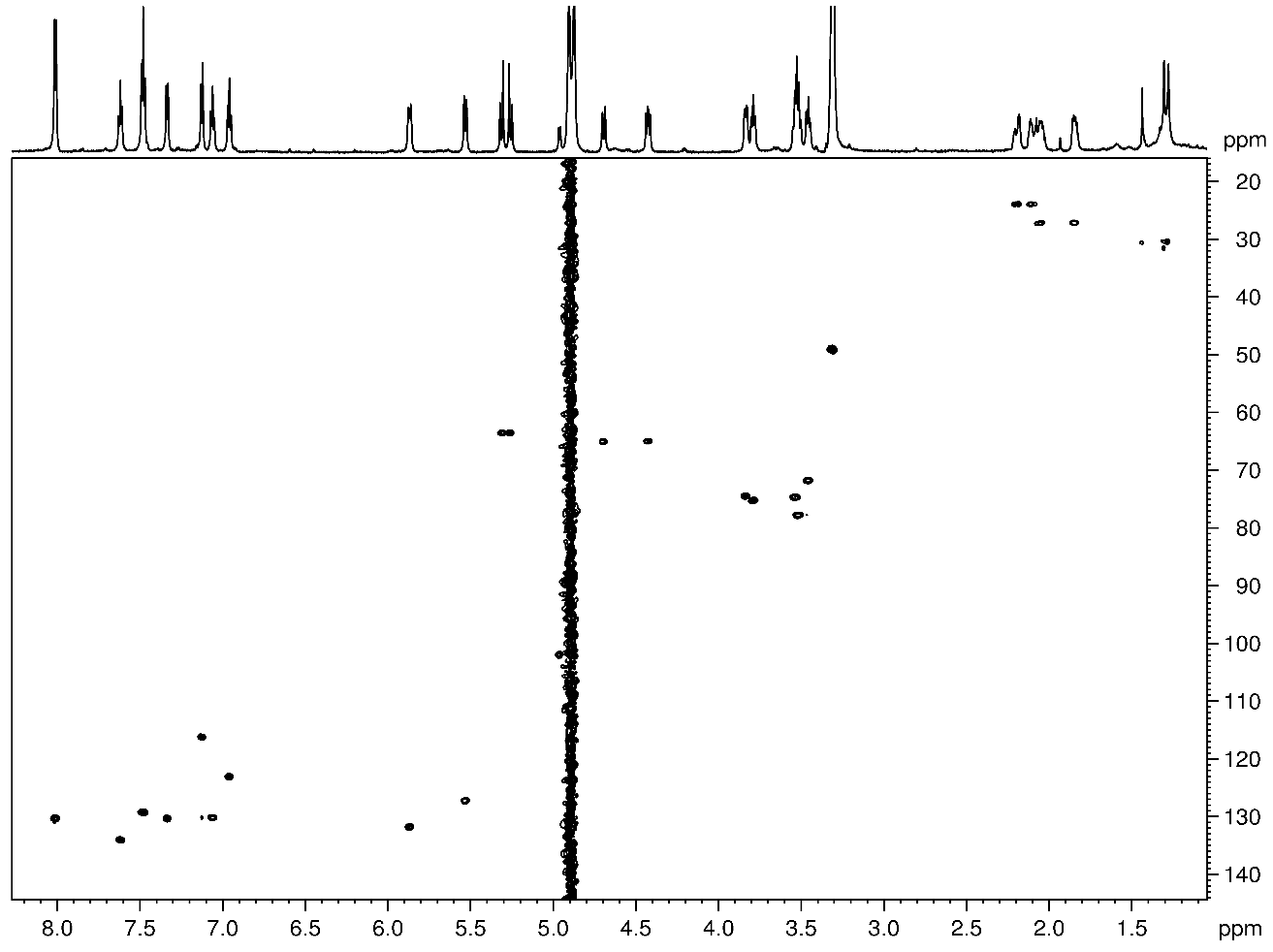


**Fig. 3**: 6’-O-benzoyl-salicortinol, ^1^H-^13^C HSQC spectrum (MeOH-d_3_)


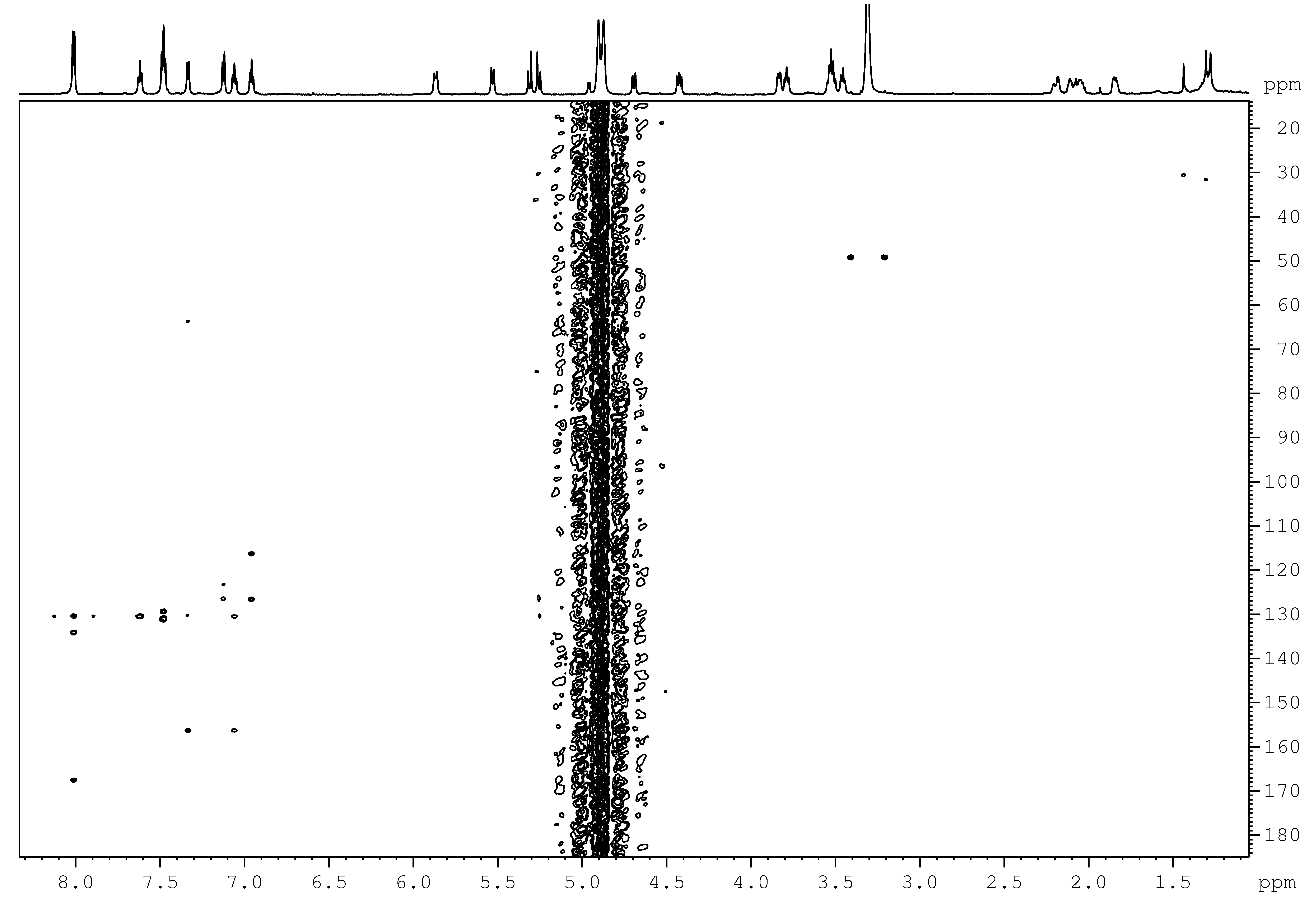


**Fig. 4**: 6’-O-benzoyl-salicortinol, ^1^H-^13^C HMBC spectrum (MeOH-d_3_)


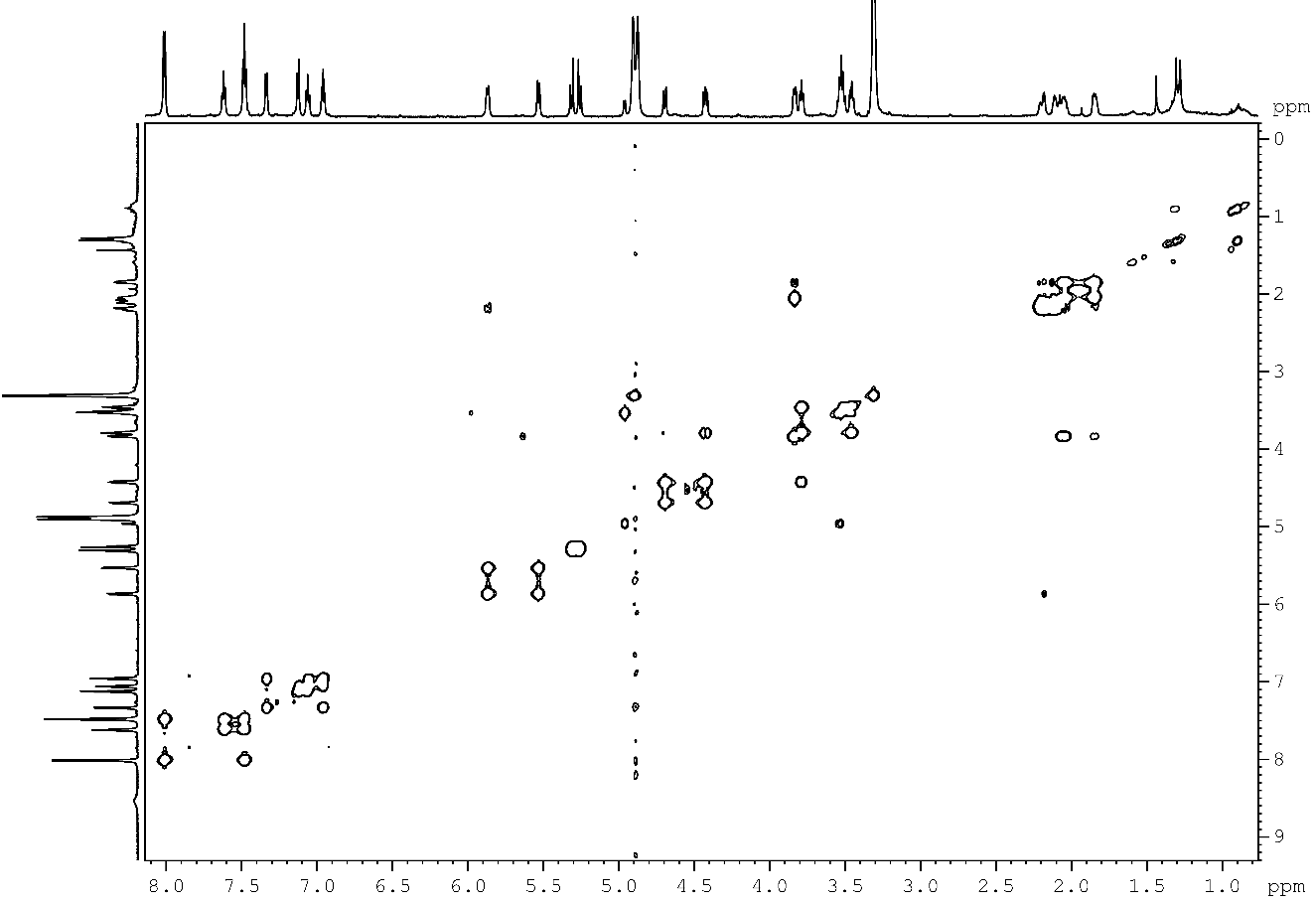


**Fig. 5**: 6’-O-benzoyl-salicortinol, ^1^H-^1^H COSY spectrum (MeOH-d_3_)


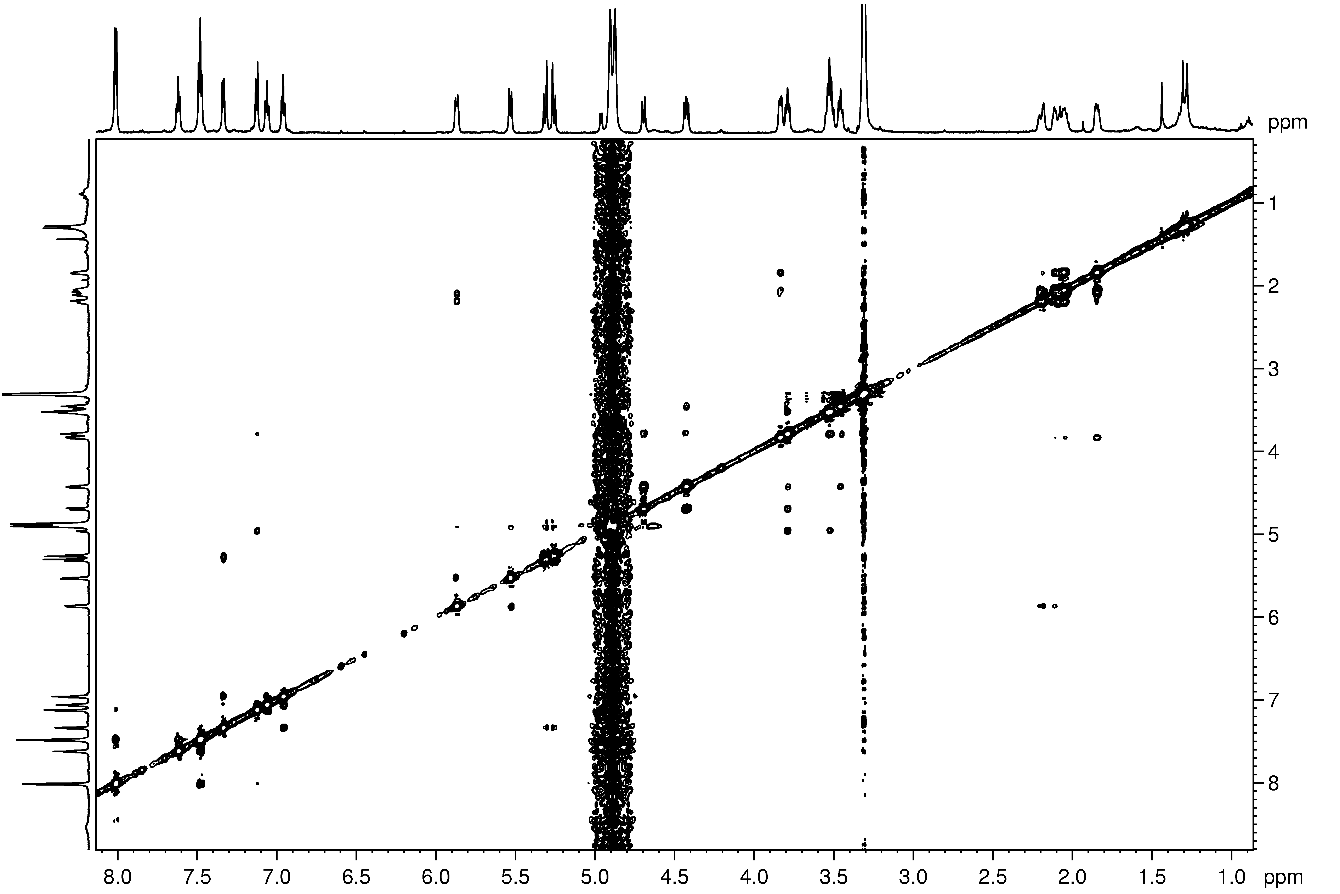


**Fig. 6**: 6’-O-benzoyl-salicortinol, ^1^H-^1^H TOCSY spectrum (MeOH-d_3_)


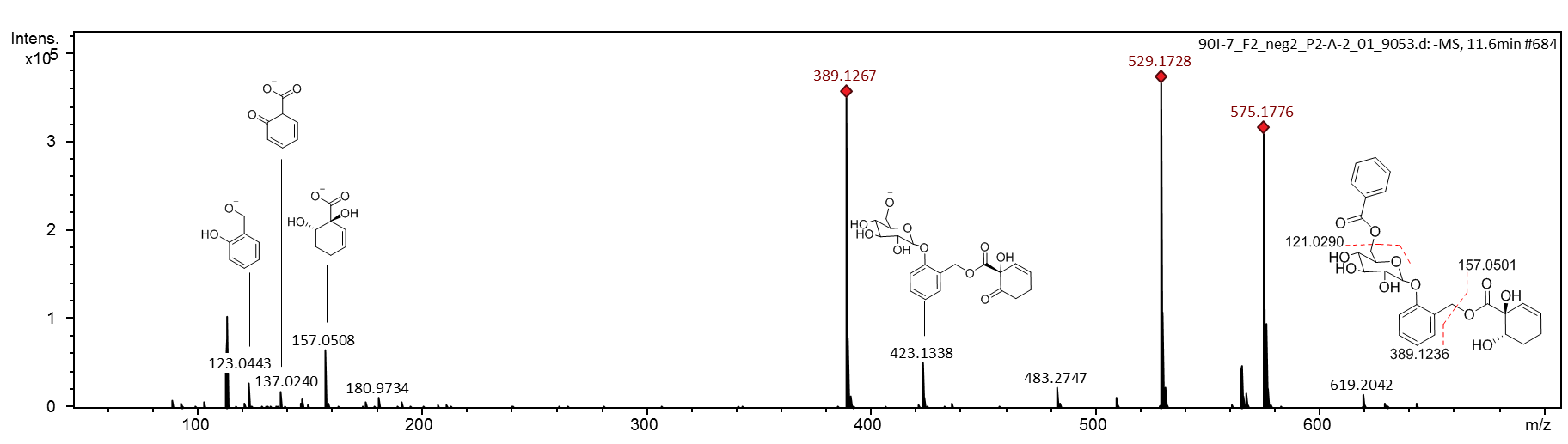


**Fig. 7**: HR-ESI-MS^2^ spectrum of 6'-O-benzoyl-salicortinol


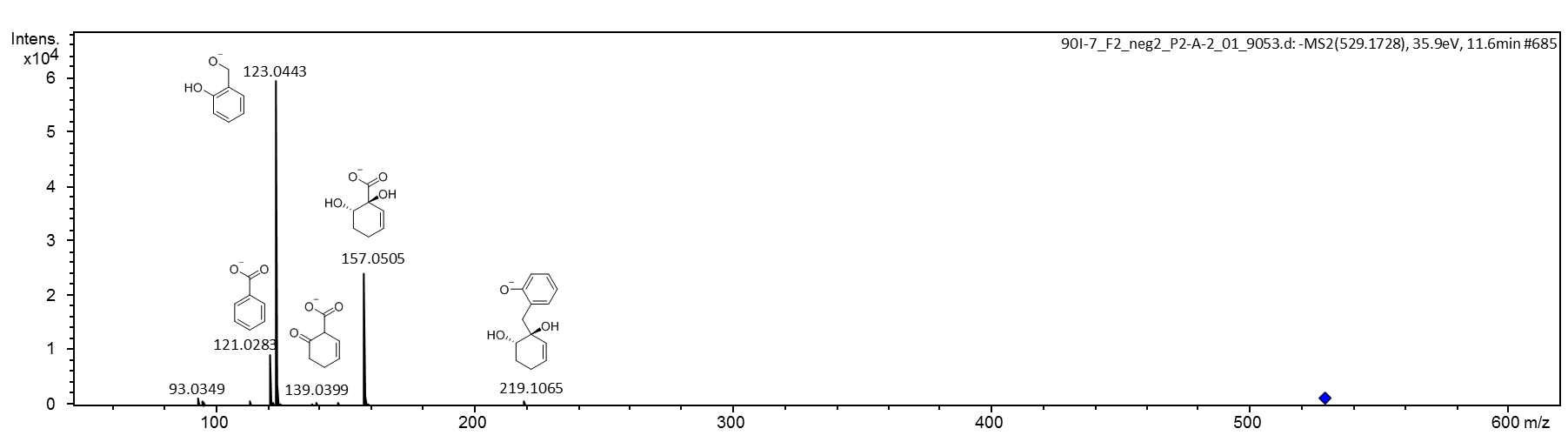


**Fig. 8**: HR-ESI-MS^2^ spectrum of 6'-O-benzoyl-salicortinol, main isotope peak

**Table 3**: Mass fragments of 6'-O-benzoyl-salicortinol

| fragment *m/z* [M-H]^-^ | sum formula | calc. fragment *m/z* | structure |
| --- | --- | --- | --- |
| 123.0443 | C_7_H_7_O_2_^-^ | 123.0452 |  |
| 121.0295 | C_7_H_6_O_2_^-^ | 121.0295 |  |
| 157.0508 | C_7_H_9_O_4_^-^ | 157.0506 |  |
| 389.1267 | C_20_H_21_O_8_^-^ | 389.1242 |  |
| 529.1728 | C_27_H_29_O_11_^-^ | 529.1715 |  |

Isolation of [U-^13^C]salicortin, [U-^13^C]HCH-salicortin and [U-^13^C]tremulacin

An extract of ^13^C-labeled *P. deltoides x trichocarpa* leaf material, prepared as described previously, was used.* An amount of 2.19 g dried extract was used for isolation of the [U-^13^C]-title compounds. After reconstitution with MeOH, aliquots (146.19 mg ml^−1^) were subjected to HPLC separation on a Macherey-Nagel (MN) Isis RP-18e column (250 × 4.6 mm, 5 μm particle size) utilizing the chromatographic conditions as described previously.* Salicortin eluted at *R*_t_ 40.2 min, trichocarpin at *R*_t_ 57.3 min, HCH-salicortin at 62.0 min, and tremulacin eluted at 86.3 min. For monitoring, the UV trace at 285 nm was used. The compounds were subsequently re-purified to remove traces of impurities, as described below.
A binary solvent system consisting of 0.1% formic acid in H_2_O (solvent A) and 0.1% formic acid in MeOH (solvent B) was used. The respective HPLC column temperature was set to 35 °C and the flow rate was set to 0.8 ml min^−1^.
*[U-^13^C]salicortin*. An aliquot solution (39.27 mg ml^−1^) was purified using a MN π^2^-column (250 × 4.6 mm, 5 μm particle size). The HPLC gradient started with 50% of solvent B and increased linearly to 62% in 12 min. Afterwards, the column was washed for 10 min with 100% solvent B, followed by a 10 min equilibration with 50% solvent B. The [U-^13^C]salicortin peak (λ = 285 nm) appeared at *R*_t_ 10.4 min. We isolated 48.19 mg of [U-^13^C]salicortin.
*[U-^13^C]HCH-salicortin.* An aliquot solution (56.02 mg ml^-1^) was purified using a MN π^^-column (250 × 4.6 mm, 5 μm particle size). The HPLC gradient started with a concentration of 40% solvent B and increased linearly to 75% solvent B in 30 min. Afterwards, the column was washed for 15 min with 100% solvent B, followed by a 10 min equilibration with 40% solvent B. The [U-^13^C]HCH-salicortin peak (λ = 285 nm) appeared at *R*_t_ 25.5 min. We isolated 45.13 mg of [U-^13^C]HCH-salicortin.
*[U-^13^C]tremulacin*. An aliquot solution (40.12 mg ml^−1^) was purified using a MN π^^-column (250 × 4.6 mm, 5 μm particle size). The HPLC gradient started with a concentration of 70% solvent B and increased linearly to 80% solvent B in 15 min. Afterwards, the column was washed for 10 min with 100% solvent B followed by an equilibration period of 10 min with 70% solvent B. The tremulacin peak (λ = 285 nm) appeared at *R*_t_ 10.6 min. We isolated 21.46 mg of [U-^13^C]tremulacin.

*) Feistel, F., *et al*., Acylated Quinic Acids Are the Main Salicortin Metabolites in the Lepidopteran Specialist Herbivore Cerura vinula. Journal of Chemical Ecology, 2018. 44(5): p. 497-509.

# Characterization of isolated ^13^C-labelled compounds

## [U-^13^C]salicortin


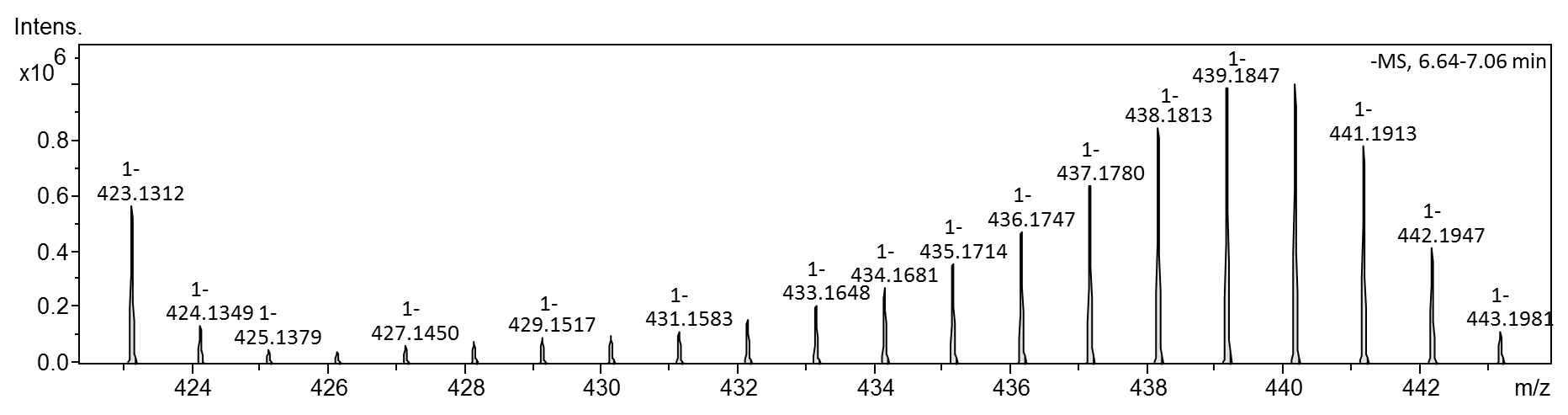


**Fig. 9**: [U-^13^C]salicortin, HR-ESI-MS spectrum, m/z 423.1308 [M-H]^-^

**Table 4**: Extracted MS data used for the calculation of the ^13^C-enrichment of salicortin isotopologues (m/z) together with their signal intensities

| C-isotope | | salicortin | |
| --- | --- | --- | --- |
| ^12^C | ^13^C | m/z | I |
| 20 | 0 | 423.1312 | 563016 |
| 19 | 1 | 424.1349 | 138922 |
| 18 | 2 | 425.1379 | 51067 |
| 17 | 3 | 426.1417 | 49058 |
| 16 | 4 | 427.1450 | 68105 |
| 15 | 5 | 428.1484 | 84101 |
| 14 | 6 | 429.1517 | 96215 |
| 13 | 7 | 430.1550 | 102883 |
| 12 | 8 | 431.1583 | 120981 |
| 11 | 9 | 432.1615 | 161279 |
| 10 | 10 | 433.1648 | 213475 |
| 9 | 11 | 434.1681 | 274441 |
| 8 | 12 | 435.1714 | 361017 |
| 7 | 13 | 436.1747 | 476937 |
| 6 | 14 | 437.1780 | 636991 |
| 5 | 15 | 438.1813 | 845567 |
| 4 | 16 | 439.1847 | 988207 |
| 3 | 17 | 440.1879 | 1004403 |
| 2 | 18 | 441.1913 | 781973 |
| 1 | 19 | 442.1947 | 419749 |
| 0 | 20 | 443.1981 | 120096 |
| ^13^C-incorporation | ~82% | R | 0.89 |

**Table 5**: Extracted MS data used for the calculation of the ^13^C-enrichment of the salicortin isotopologues (m/z) together with their signal intensities

|  | HCH | | | saligenin | | | salicin | | |
| --- | --- | --- | --- | --- | --- | --- | --- | --- | --- |
|  |  | | |  | | |  | | |
|  | 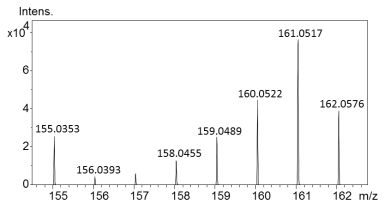 | | | 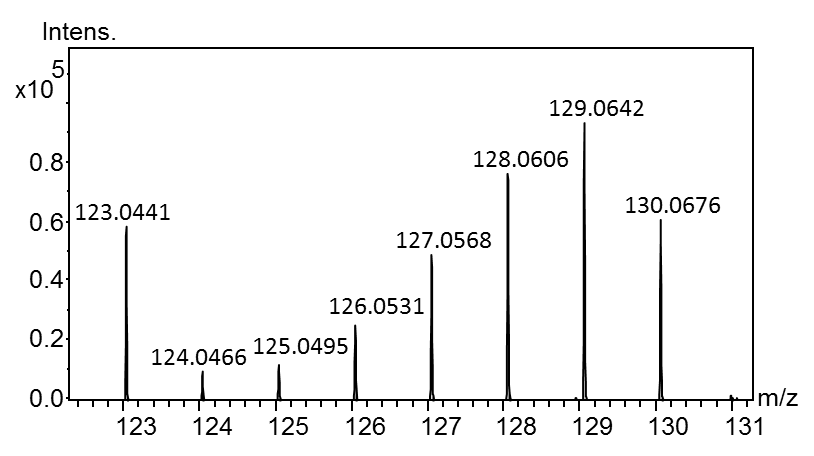 | | | 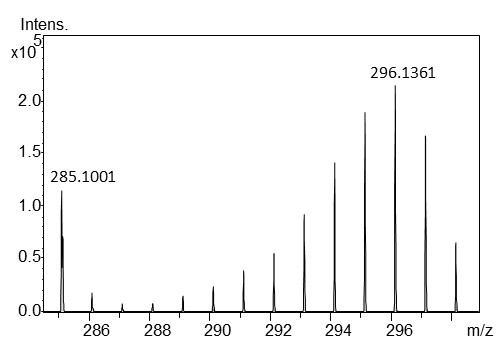 | | |
| ^13^C | m/z | intensity |  | m/z | intensity |  | m/z | intensity |  |
| 0 | 155.0353 | 25763 |  | 123.0441 | 58980 |  | 285.1002 | 117091 |  |
| 1 | 156.0393 | 4418 |  | 124.0466 | 9833 |  | 286.1033 | 18886 |  |
| 2 | 157.0427 | 6313 |  | 125.0495 | 12312 |  | 287.1062 | 7468 |  |
| 3 | 158.0455 | 12927 |  | 126.0531 | 25394 |  | 288.11 | 7977 |  |
| 4 | 159.0489 | 25066 |  | 127.0568 | 49210 |  | 289.1134 | 12761 |  |
| 5 | 160.0522 | 44823 |  | 128.0606 | 76654 |  | 290.1167 | 19617 |  |
| 6 | 161.0517 | 76388 |  | 129.0642 | 93410 |  | 291.1201 | 29141 |  |
| 7 | 162.0576 | 39063 |  | 130.0676 | 60705 |  | 292.1234 | 42306 |  |
| 8 |  |  |  |  |  |  | 293.1268 | 64903 |  |
| 9 |  |  |  |  |  |  | 294.13 | 95503 |  |
| 10 |  |  |  |  |  |  | 295.1333 | 125788 |  |
| 11 |  |  |  |  |  |  | 296.1366 | 139181 |  |
| 12 |  |  |  |  |  |  | 297.14 | 105141 |  |
| 13 |  |  |  |  |  |  | 298.1435 | 42043 |  |

**Fig. 10**: [U-^13^C]salicortin, structure with chemical shifts (MeOH-d_3_)

**
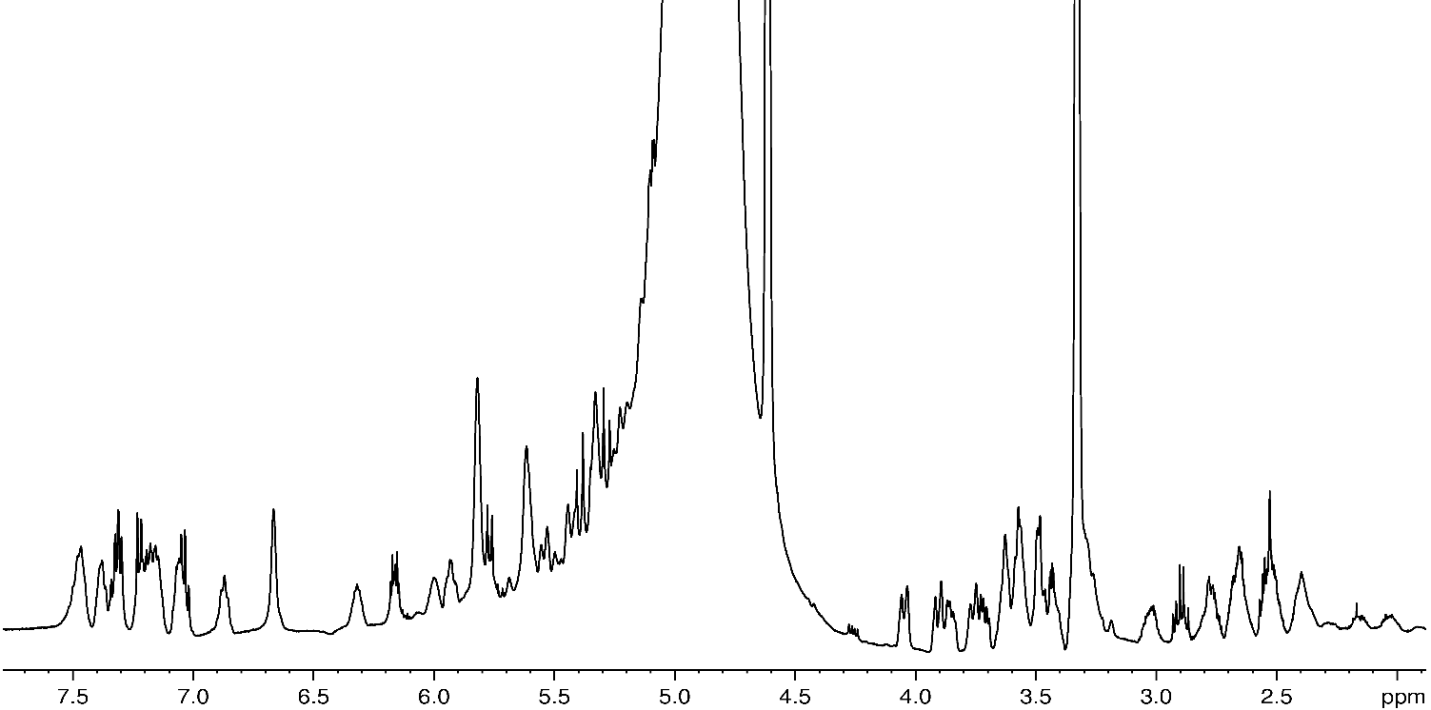
**

**Fig. 11**: [U-^13^C]salicortin, ­^1^H-NMR spectrum (MeOH-d_3_)


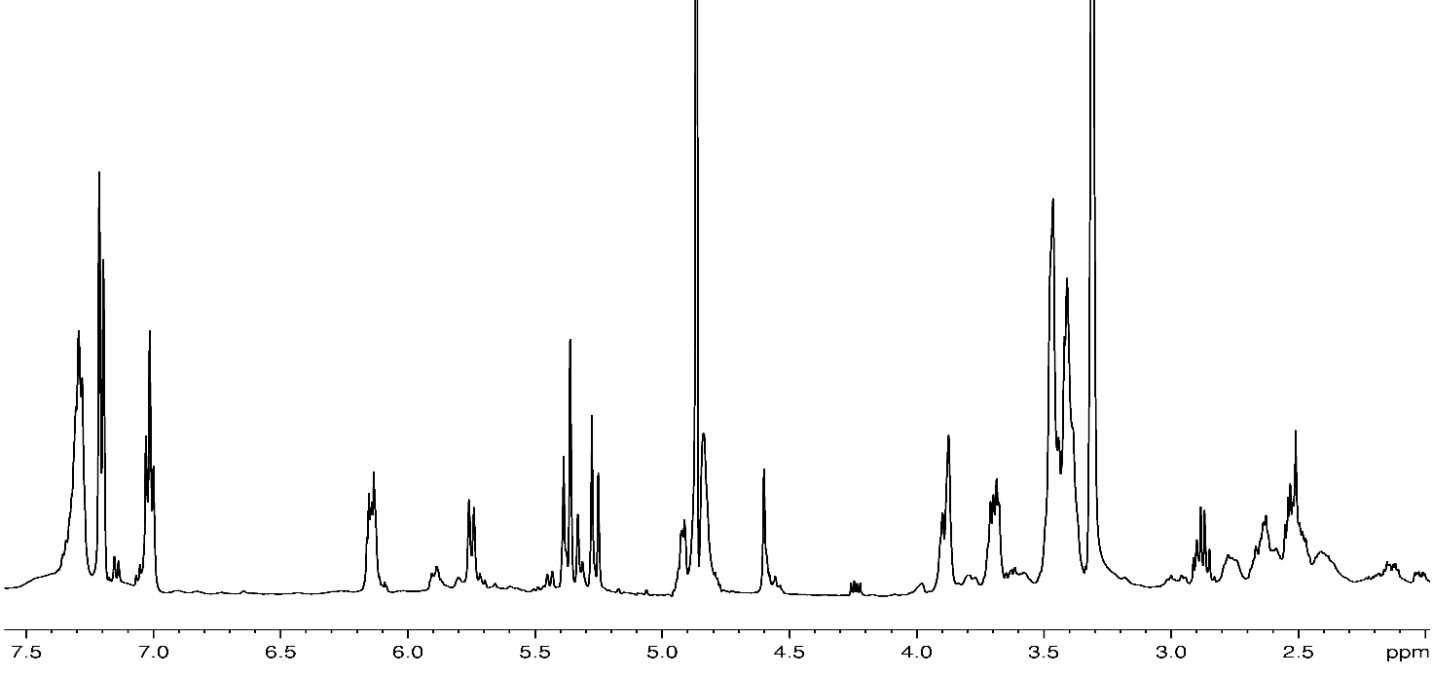


**Fig. 12**: [U-^13^C]salicortin, ­^1^H-NMR spectrum, ^13^C-decoupled (MeOH-d_3_)

**
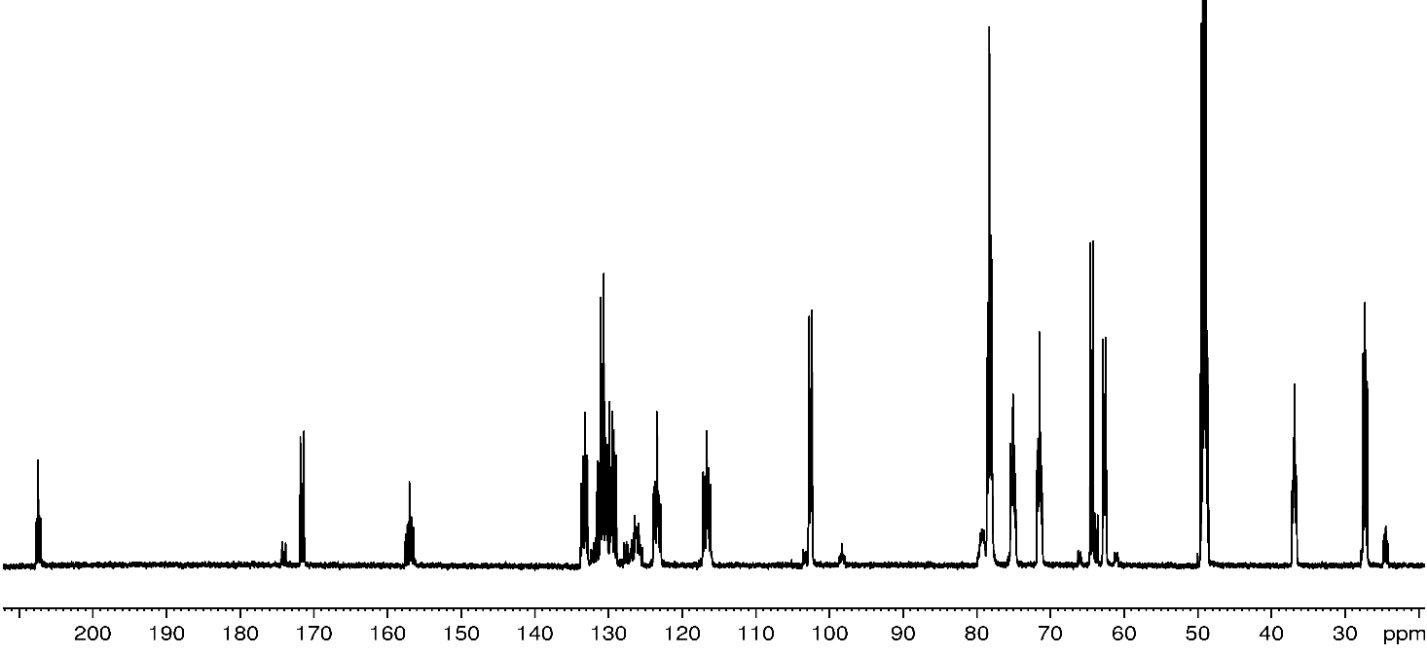
**

**Fig. 13**: [U-^13^C]salicortin, ^13^C-NMR spectrum (MeOH-d_3_)

**
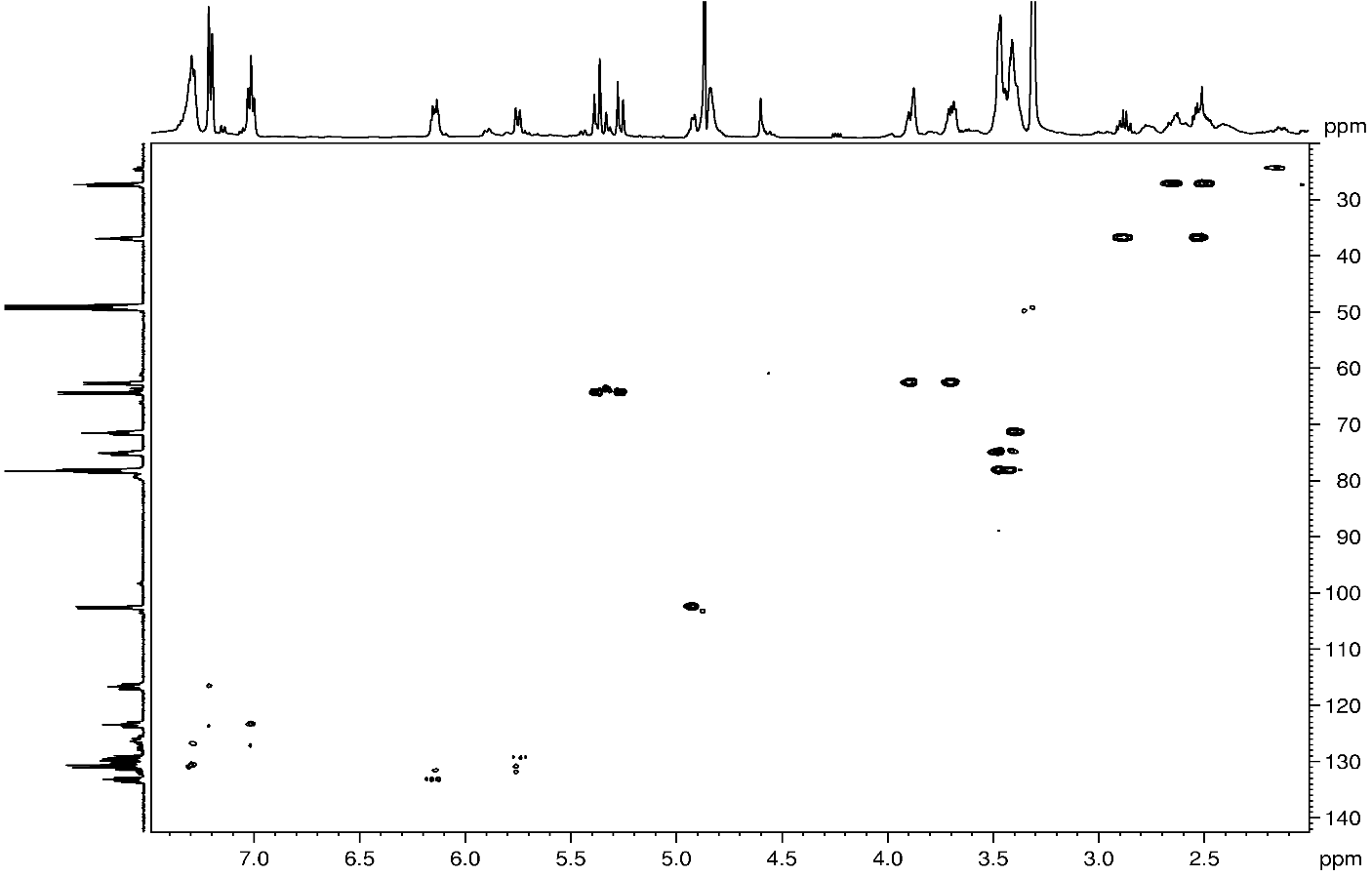
**

**Fig. 14**: [U-^13^C]salicortin, ^1^H-^13^C HSQC spectrum (MeOH-d_3_)


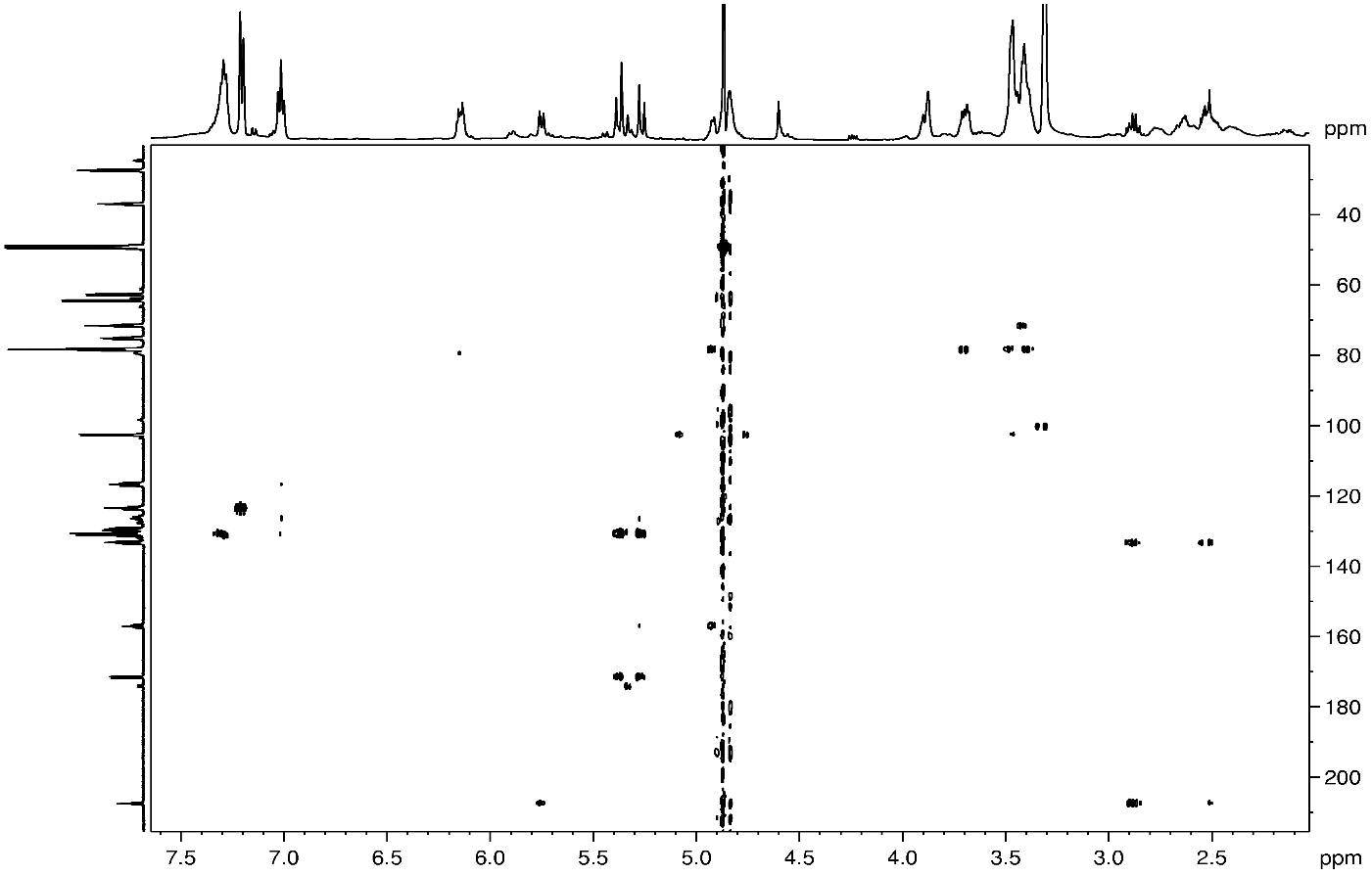


**Fig. 15**: [U-^13^C]salicortin, ^1^H-^13^C HMBC spectrum (MeOH-d_3_)


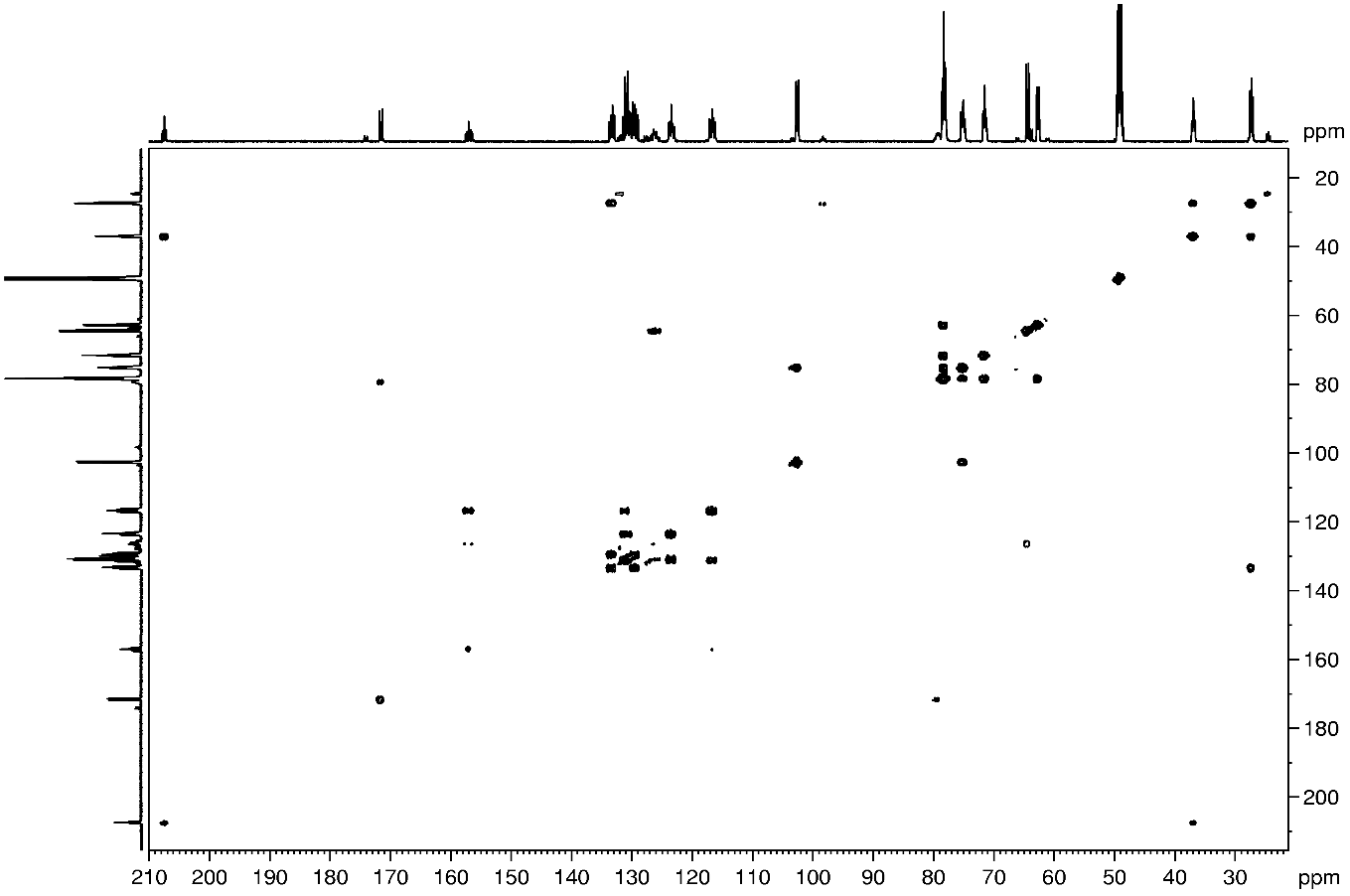


**Fig. 16**: [U-^13^C]salicortin, ^13^C-^13^C COSY spectrum (MeOH-d_3_)


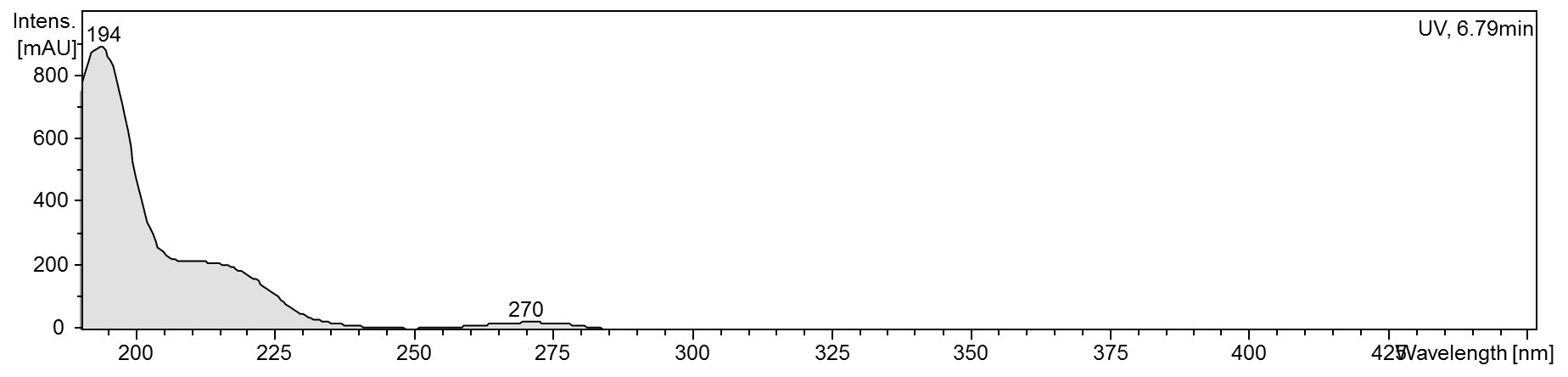


**Fig. 17**: [U-^13^C]salicortin, UV spectrum from HPLC-DAD

## [U-^13^C]HCH-salicortin


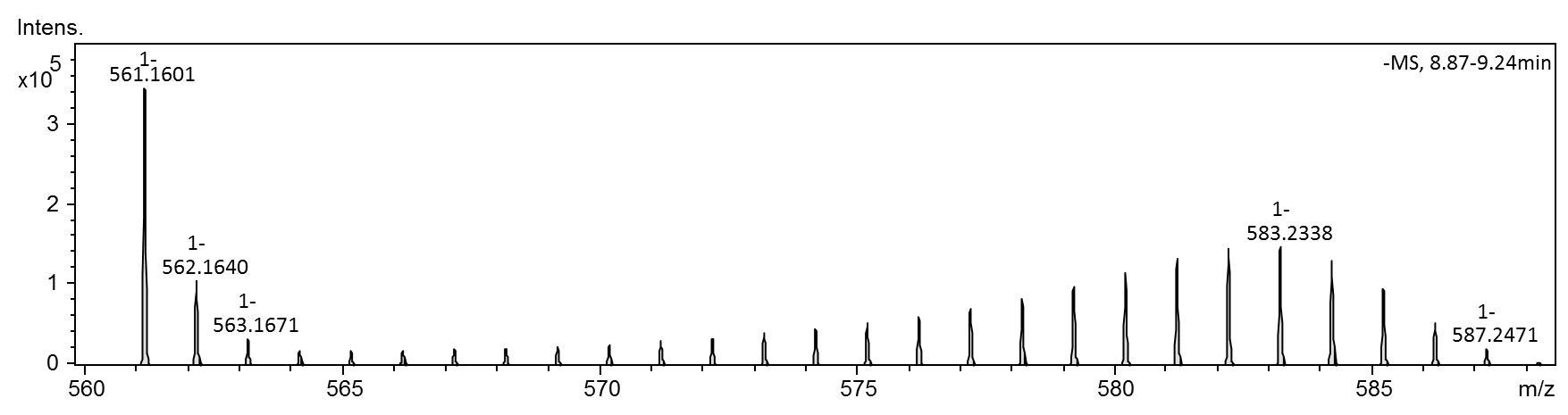


**Fig. 18**: [U-^13^C]HCH-salicortin, HRESIMS spectrum, m/z 561.1607 [M-H]^-^

**Table 6**: Extracted MS data used for calculation of the ^13^C-enrichment of the HCH-salicortin isotopologues (m/z) together with their signal intensities

| C-isotope | | HCH-salicortin | |
| --- | --- | --- | --- |
| ^12^C | ^13^C | m/z | intensity |
| 27 | 0 | 561.1601 | 345519 |
| 26 | 1 | 562.164 | 106129 |
| 25 | 2 | 563.1671 | 32580 |
| 24 | 3 | 564.1709 | 18745 |
| 23 | 4 | 565.1748 | 18666 |
| 22 | 5 | 566.1778 | 19896 |
| 21 | 6 | 567.1811 | 20099 |
| 20 | 7 | 568.1845 | 20410 |
| 19 | 8 | 569.1875 | 22764 |
| 18 | 9 | 570.1909 | 25813 |
| 17 | 10 | 571.1942 | 30439 |
| 16 | 11 | 572.1973 | 34320 |
| 15 | 12 | 573.2008 | 40010 |
| 14 | 13 | 574.204 | 46564 |
| 13 | 14 | 575.2075 | 52914 |
| 12 | 15 | 576.2108 | 61142 |
| 11 | 16 | 577.2138 | 71679 |
| 10 | 17 | 578.2171 | 83471 |
| 9 | 18 | 579.2205 | 97754 |
| 8 | 19 | 580.224 | 115086 |
| 7 | 20 | 581.2273 | 132612 |
| 6 | 21 | 582.2305 | 145940 |
| 5 | 22 | 583.2338 | 148565 |
| 4 | 23 | 584.2372 | 131557 |
| 3 | 24 | 585.2406 | 96894 |
| 2 | 25 | 586.2441 | 54139 |
| 1 | 26 | 587.2471 | 21082 |
| 0 | 27 | 588.2503 | 4863 |
| ^13^C-incorporation | ~77% | R | 0.80 |

**Table 7**: Extracted MS data used for the calculation of ^13^C-enrichment of HCH-salicortin isotopologues (m/z) together with their signal intensities

|  | HCH | | | saligenin | | | salicin | | |
| --- | --- | --- | --- | --- | --- | --- | --- | --- | --- |
|  |  | | |  | | |  | | |
|  | 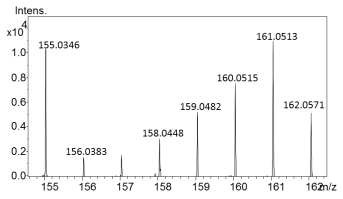 | | | 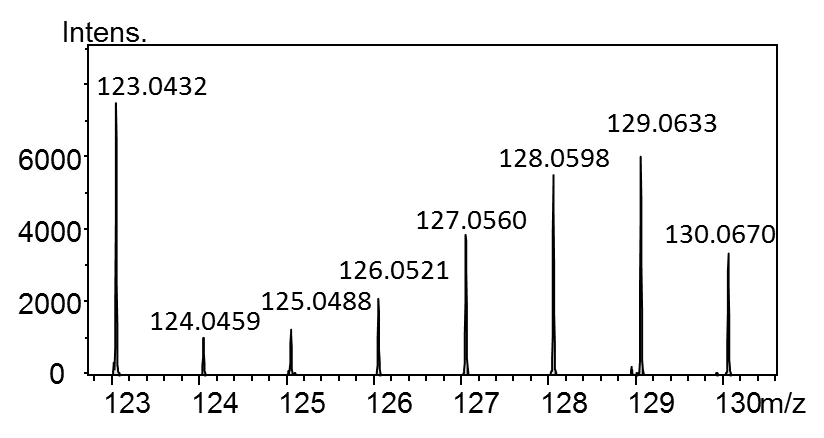 | | | 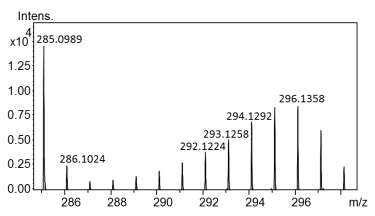 | | |
| ^13^C | m/z | intensity |  | m/z | intensity |  | m/z | intensity |  |
| 0 | 155.0346 | 10285 |  | 7490 | 7490 |  | 285.0989 | 14497 |  |
| 1 | 156.0383 | 1578 |  | 1070 | 1070 |  | 286.1024 | 2435 |  |
| 2 | 157.0414 | 1732 |  | 1299 | 1299 |  | 287.1041 | 937 |  |
| 3 | 158.0448 | 3062 |  | 2138 | 2138 |  | 288.1081 | 1004 |  |
| 4 | 159.0482 | 5221 |  | 3888 | 3888 |  | 289.1116 | 1368 |  |
| 5 | 160.0515 | 7548 |  | 5484 | 5484 |  | 290.116 | 1945 |  |
| 6 | 161.0513 | 10896 |  | 6041 | 6041 |  | 291.1192 | 2730 |  |
| 7 | 162.0571 | 5169 |  | 3370 | 3370 |  | 292.1224 | 3825 |  |
| 8 |  |  |  |  |  |  | 293.1258 | 5143 |  |
| 9 |  |  |  |  |  |  | 294.1292 | 6842 |  |
| 10 |  |  |  |  |  |  | 295.1323 | 8367 |  |
| 11 |  |  |  |  |  |  | 296.1358 | 8430 |  |
| 12 |  |  |  |  |  |  | 297.1391 | 6080 |  |
| 13 |  |  |  |  |  |  | 298.1428 | 2342 |  |

**Fig. 19**: [U-^13^C]HCH-salicortin, chemical structure with chemical shifts (MeOH-d_3_)

**
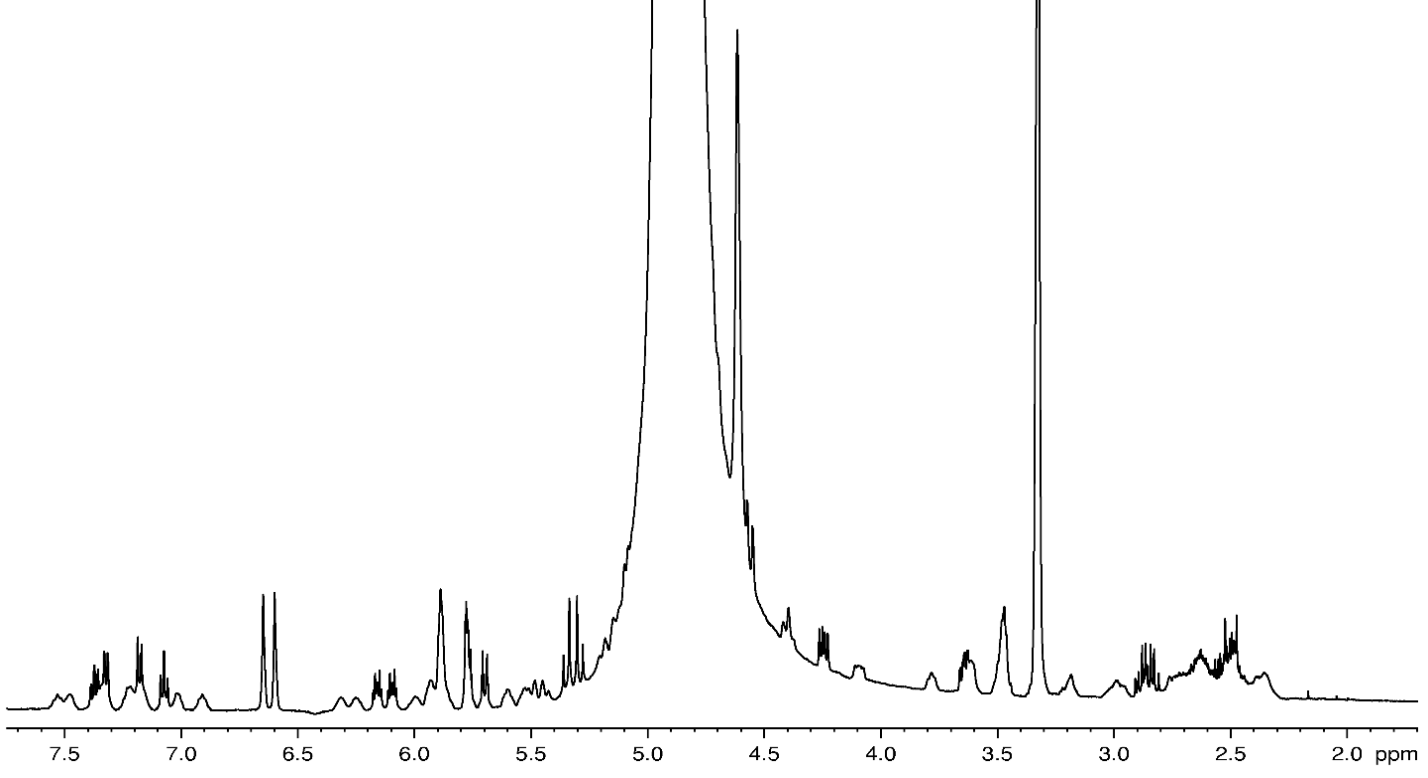
**

**Fig. 20**: [U-^13^C]HCH-salicortin, ^1^H-NMR spectrum (MeOH-d_3_)


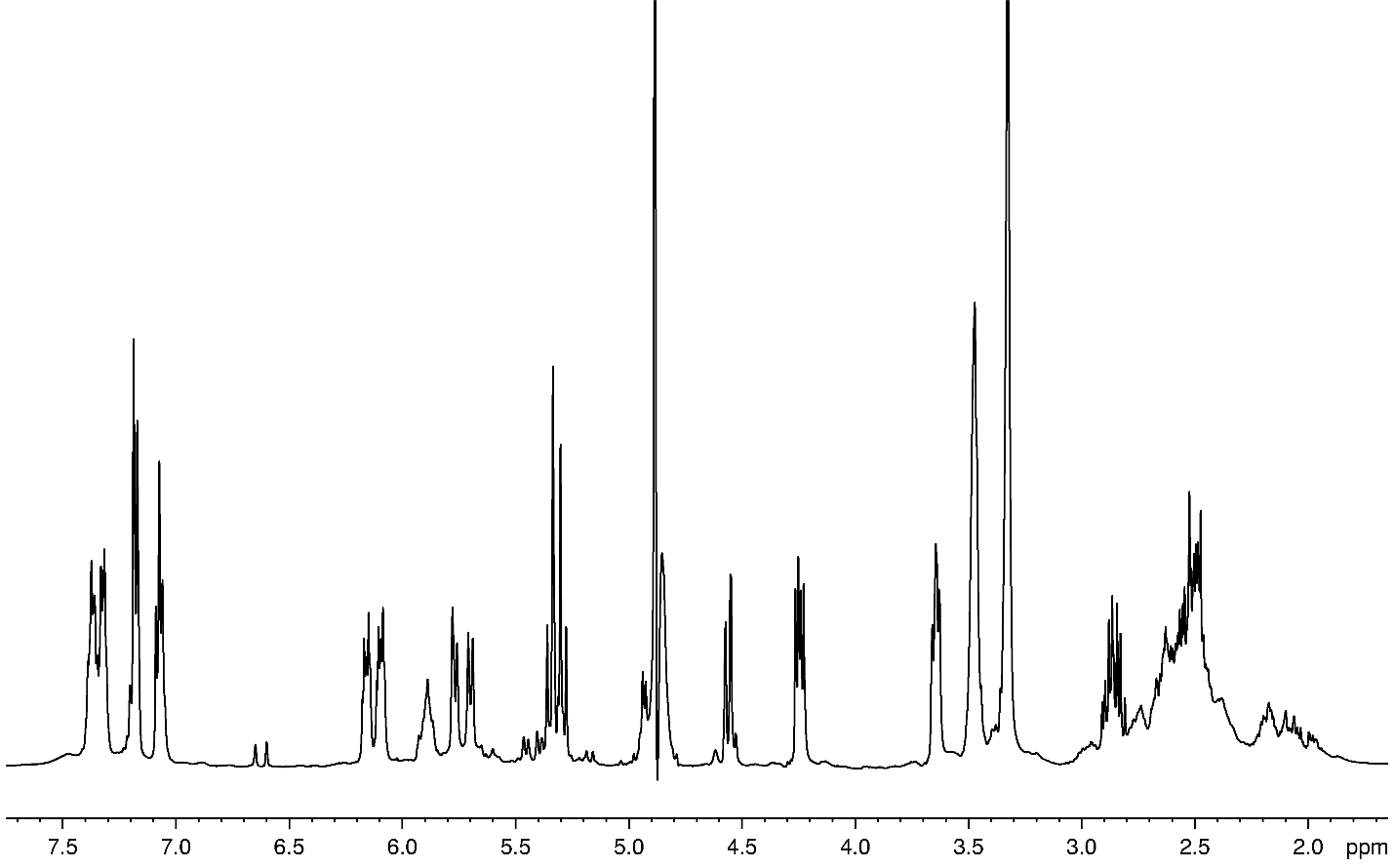


**Fig. 21**: [U-^13^C]HCH-salicortin, ^1^H-NMR spectrum, ^13^C-decoupled (MeOH-d_3_)

**
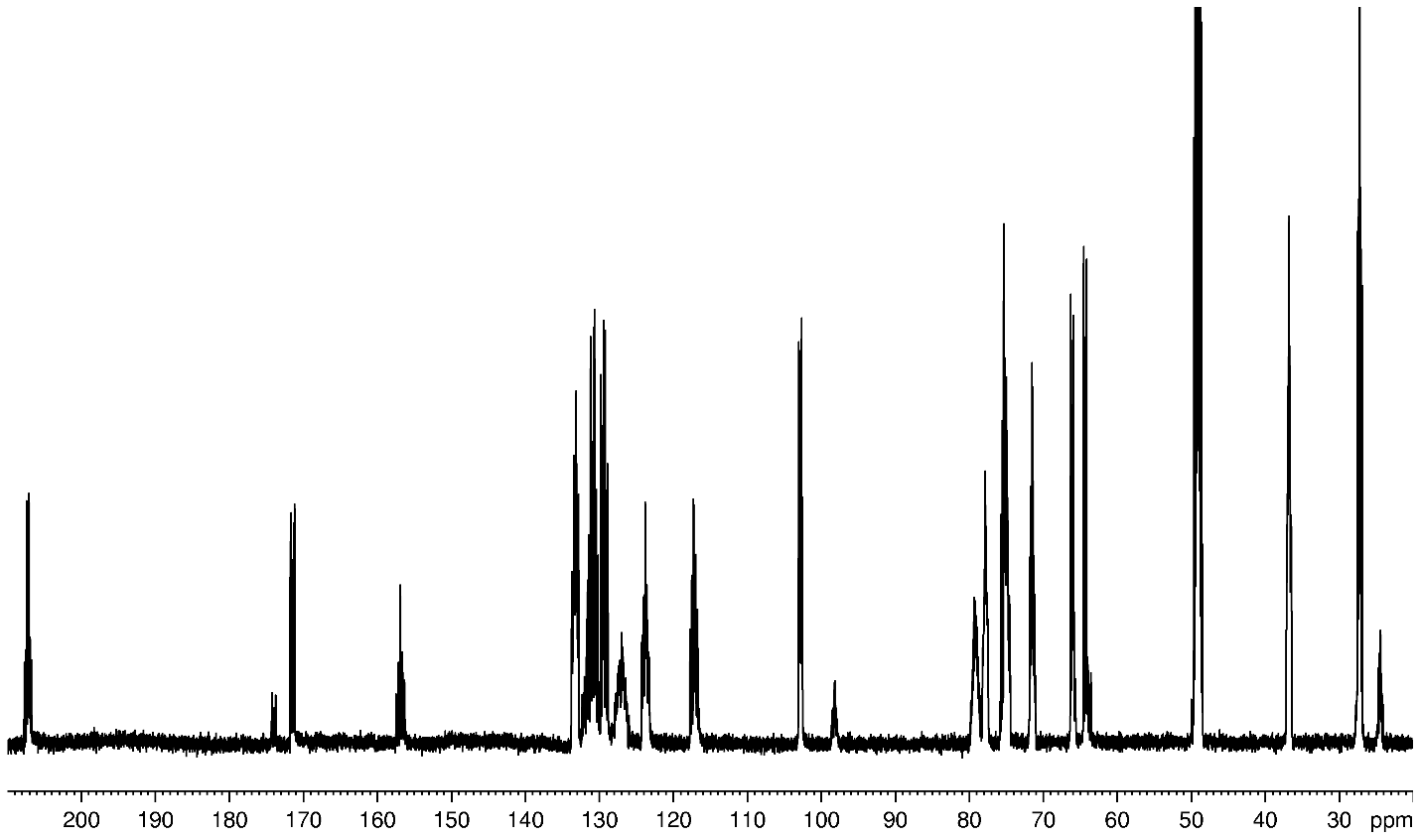
**

**Fig. 22**: [U-^13^C]HCH-salicortin, ^13^C-NMR spectrum (MeOH-d_3_)

**
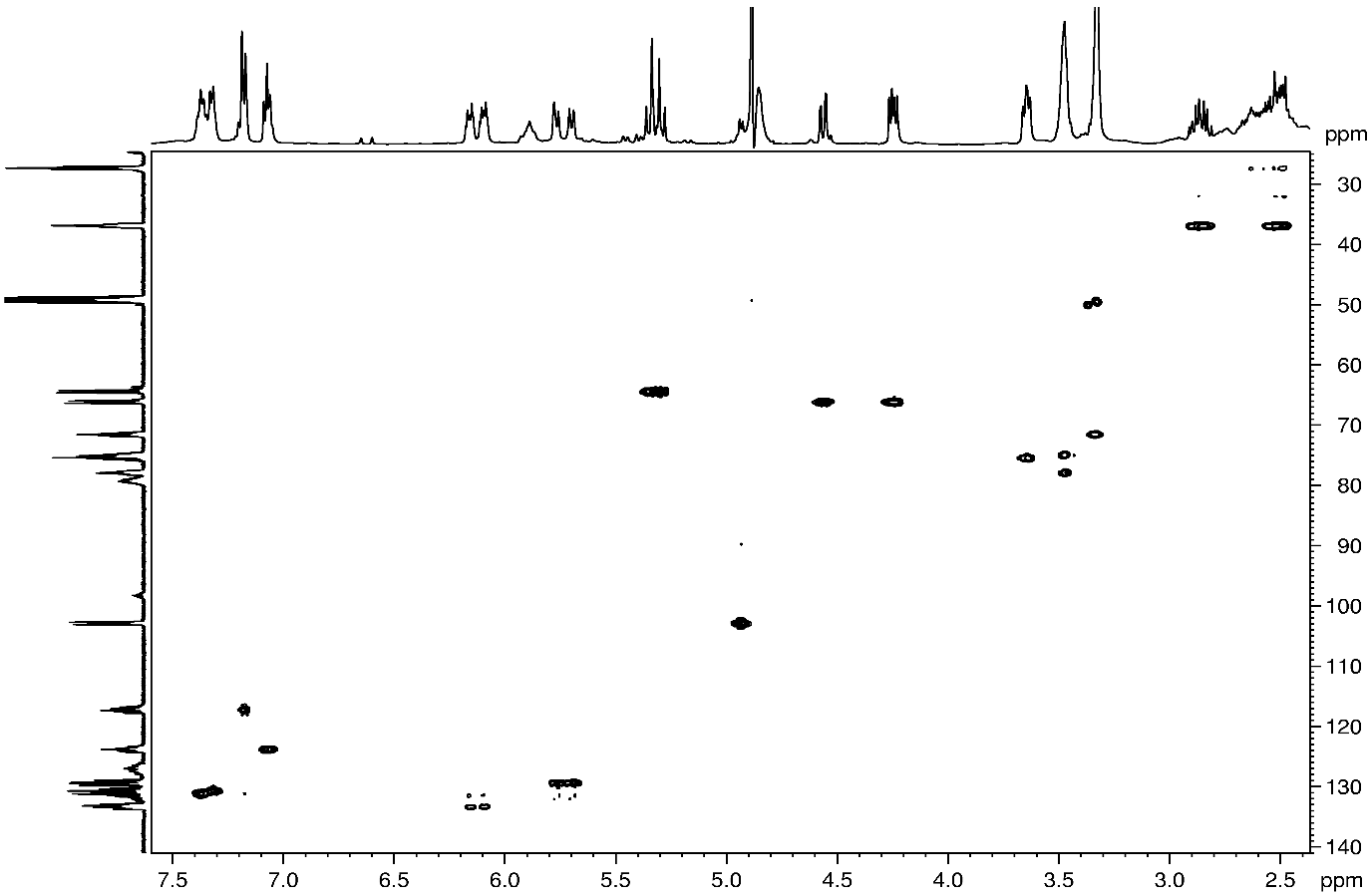
**

**Fig. 23**: [U-^13^C]HCH-salicortin, ^1^H-^13^C HSQC spectrum (MeOH-d_3_)


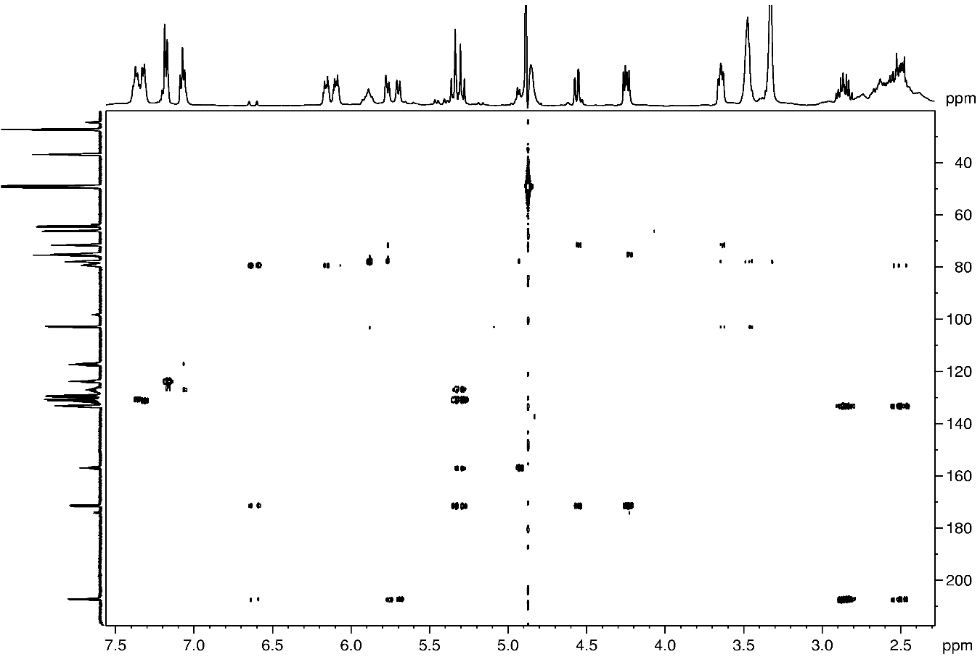


**Fig. 24**: [U-^13^C]HCH-salicortin, ^1^H-^13^C HMBC spectrum (MeOH-d_3_)
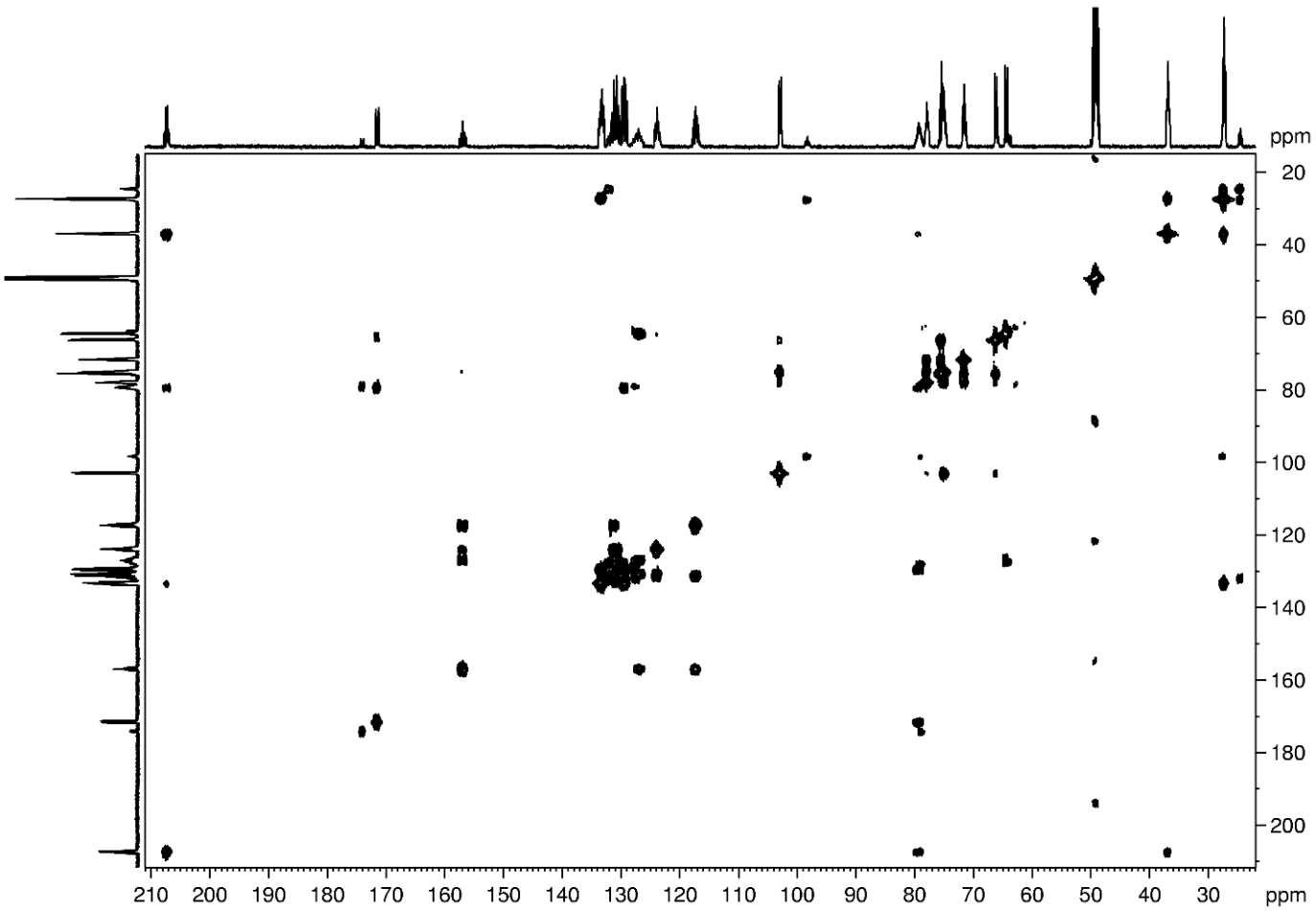


**Fig. 25**: [U-^13^C]HCH-salicortin, ^13^C-^13^C COSY spectrum (MeOH-d_3_)


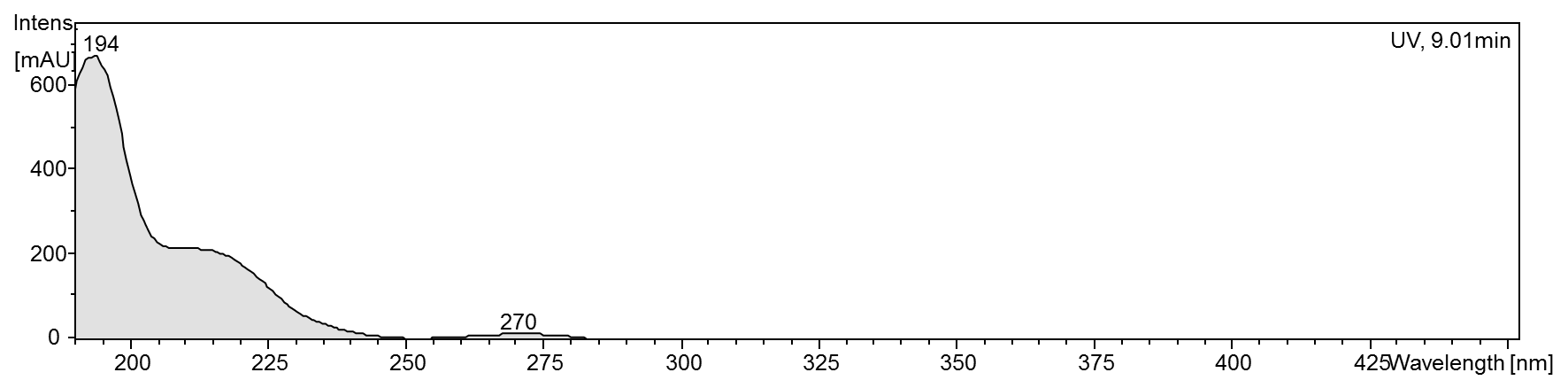


**Fig. 26**: [U-^13^C]HCH-salicortin, UV spectrum from HPLC-DAD

## [U-^13^C]Tremulacin


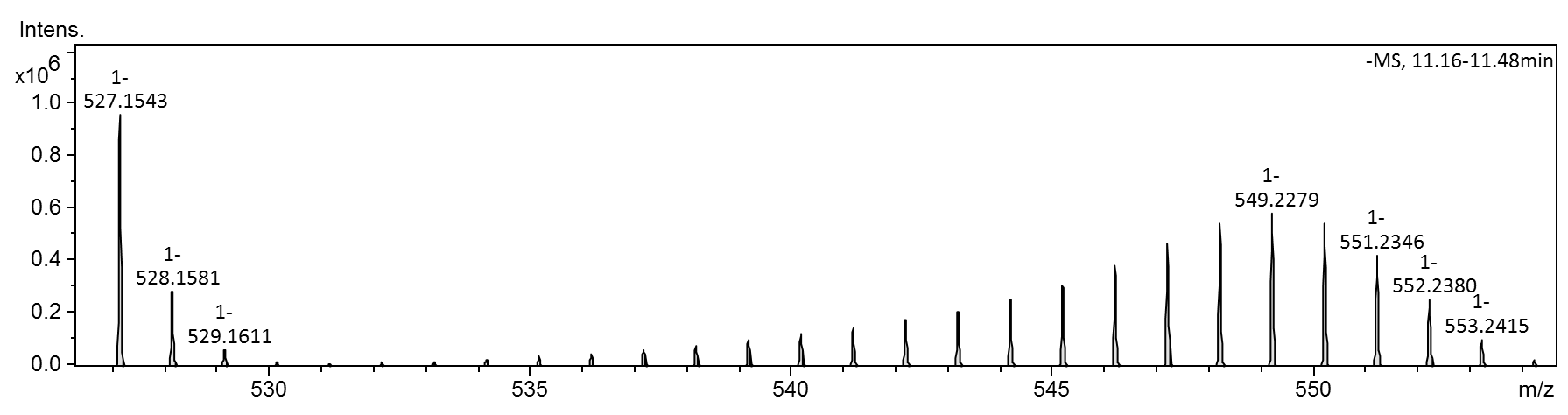


**Fig. 27**: [U-^13^C]tremulacin, HR-ESI-MS spectrum, m/z 527.1572 [M-H]^-^

**Table 8**: Extracted MS data used for the calculation of ^13^C-enrichment of tremulacin isotopologues (m/z) together with their signal intensities

| C-isotope | | tremulacin | |
| --- | --- | --- | --- |
| ^12^C | ^13^C | m/z | intensity |
| 27 | 0 | 527.1543 | 958140 |
| 26 | 1 | 528.1581 | 289642 |
| 25 | 2 | 529.1611 | 67438 |
| 24 | 3 | 530.1648 | 18289 |
| 23 | 4 | 531.1686 | 13320 |
| 22 | 5 | 532.172 | 16560 |
| 21 | 6 | 533.1755 | 22099 |
| 20 | 7 | 534.1786 | 29738 |
| 19 | 8 | 535.1817 | 38929 |
| 18 | 9 | 536.185 | 50561 |
| 17 | 10 | 537.1885 | 65170 |
| 16 | 11 | 538.1917 | 82759 |
| 15 | 12 | 539.1949 | 101433 |
| 14 | 13 | 540.1982 | 124449 |
| 13 | 14 | 541.2015 | 148680 |
| 12 | 15 | 542.2047 | 177172 |
| 11 | 16 | 543.208 | 212284 |
| 10 | 17 | 544.2113 | 255830 |
| 9 | 18 | 545.2146 | 312113 |
| 8 | 19 | 546.2179 | 381792 |
| 7 | 20 | 547.2212 | 467183 |
| 6 | 21 | 548.2246 | 543118 |
| 5 | 22 | 549.2279 | 580236 |
| 4 | 23 | 550.2311 | 547797 |
| 3 | 24 | 551.2346 | 425374 |
| 2 | 25 | 552.238 | 253339 |
| 1 | 26 | 553.2415 | 101815 |
| 0 | 27 | 554.2447 | 23895 |
| ^13^C-incorporation | ~79% | R | 0.87 |

**Table 9**: Extracted MS data used for calculation of ^13^C-enrichment of the tremulacin isotopologues (m/z) together with their signal intensities

|  | HCH | | | benzoic acid | | | salicin | | |
| --- | --- | --- | --- | --- | --- | --- | --- | --- | --- |
|  |  | | |  | | |  | | |
|  | 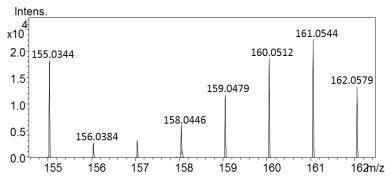 | | | 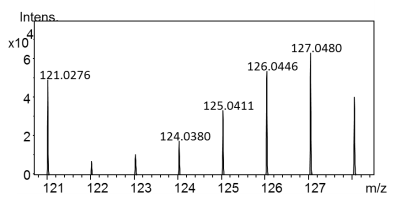 | | | 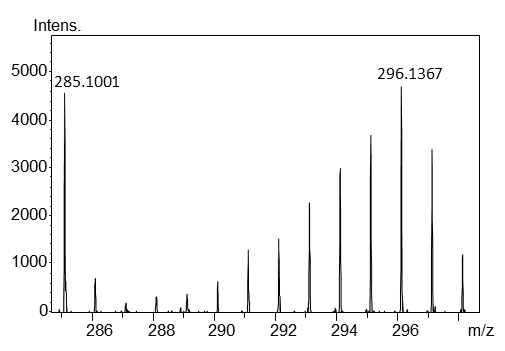 | | |
| ^13^C | m/z | intensity |  | m/z | intensity |  | m/z | intensity |  |
| 0 | 155.0344 | 18158 |  | 121.0283 | 44931 |  | 285.0982 | 2390 |  |
| 1 | 156.0384 | 2801 |  | 122.0323 | 6081 |  | 286.1005 | 367 |  |
| 2 | 157.0415 | 3284 |  | 123.0393 | 10539 |  | 287.1027 | 175 |  |
| 3 | 157.9304 | 217 |  | 124.0389 | 16257 |  | 288.1076 | 196 |  |
| 4 | 158.0446 | 6331 |  | 125.0419 | 32067 |  | 289.1106 | 306 |  |
| 5 | 159.0479 | 11764 |  | 126.0455 | 49367 |  | 290.1139 | 379 |  |
| 6 | 160.0512 | 18492 |  | 127.0488 | 60692 |  | 291.1173 | 742 |  |
| 7 | 161.0544 | 22147 |  | 128.0529 | 39599 |  | 292.1209 | 854 |  |
| 8 |  |  |  |  |  |  | 293.1252 | 1323 |  |
| 9 |  |  |  |  |  |  | 294.1279 | 1731 |  |
| 10 |  |  |  |  |  |  | 295.1317 | 2339 |  |
| 11 |  |  |  |  |  |  | 296.1353 | 2488 |  |
| 12 |  |  |  |  |  |  | 297.1394 | 1898 |  |
| 13 |  |  |  |  |  |  | 298.1428 | 725 |  |

**Fig. 28**: [U-^13^C]tremulacin, chemical structure with chemical shifts (MeOH-d_3_)

**
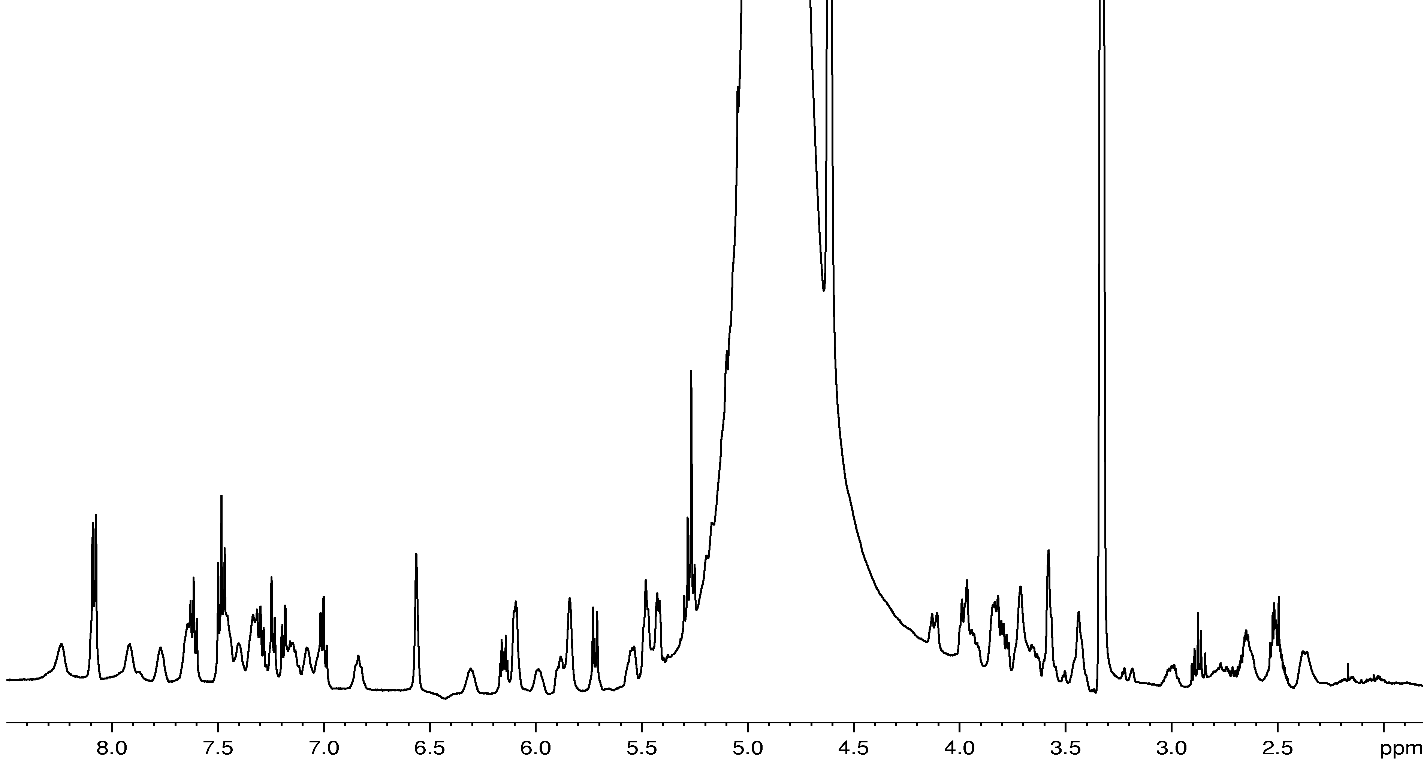
**

**Fig. 29**: [U-^13^C]tremulacin, ­^1^H-NMR spectrum (MeOH-d_3_)


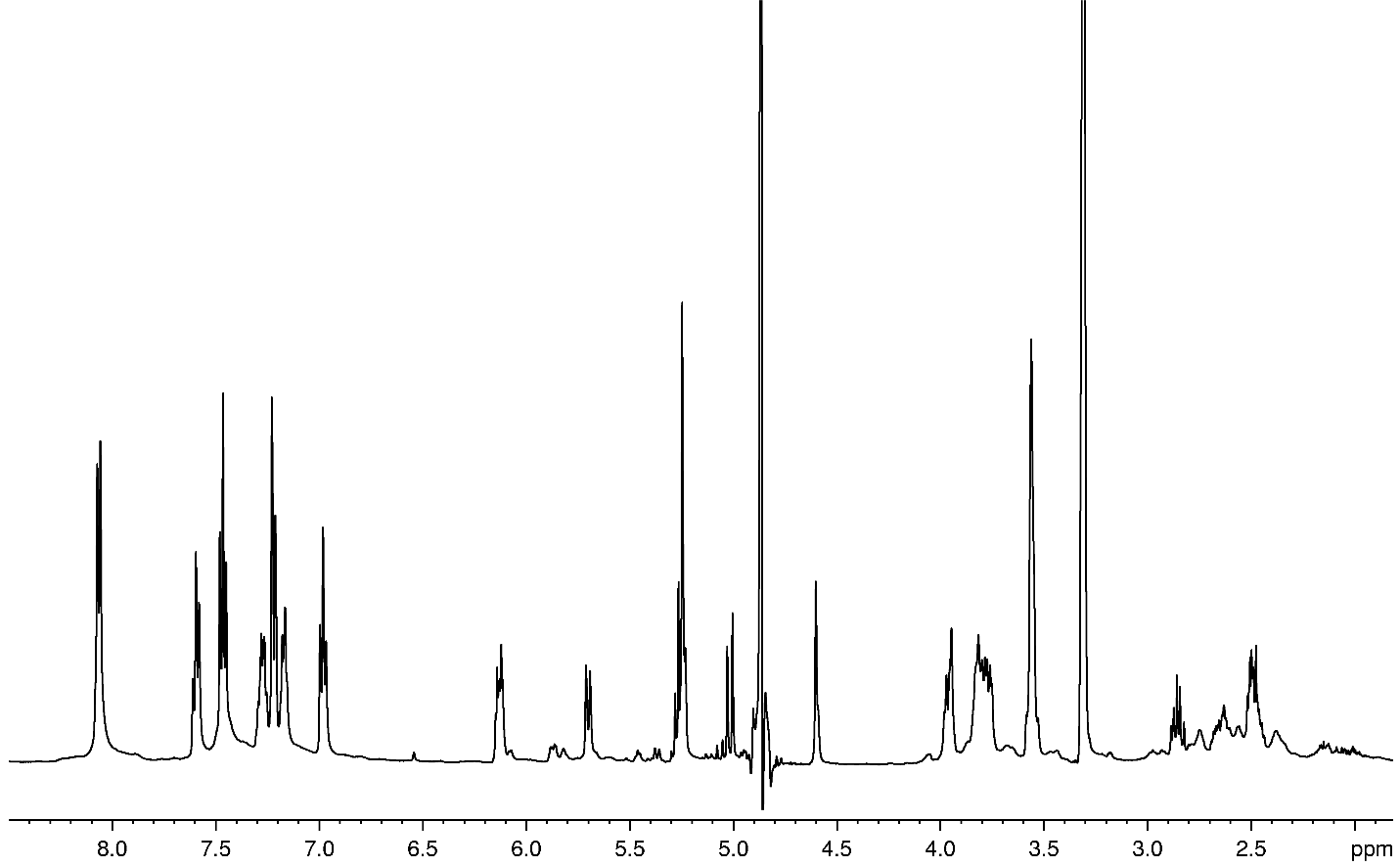


**Fig. 30**: [U-^13^C]tremulacin, ^1^H-NMR spectrum, ^13^C-decoupled (MeOH-d_3_)

**
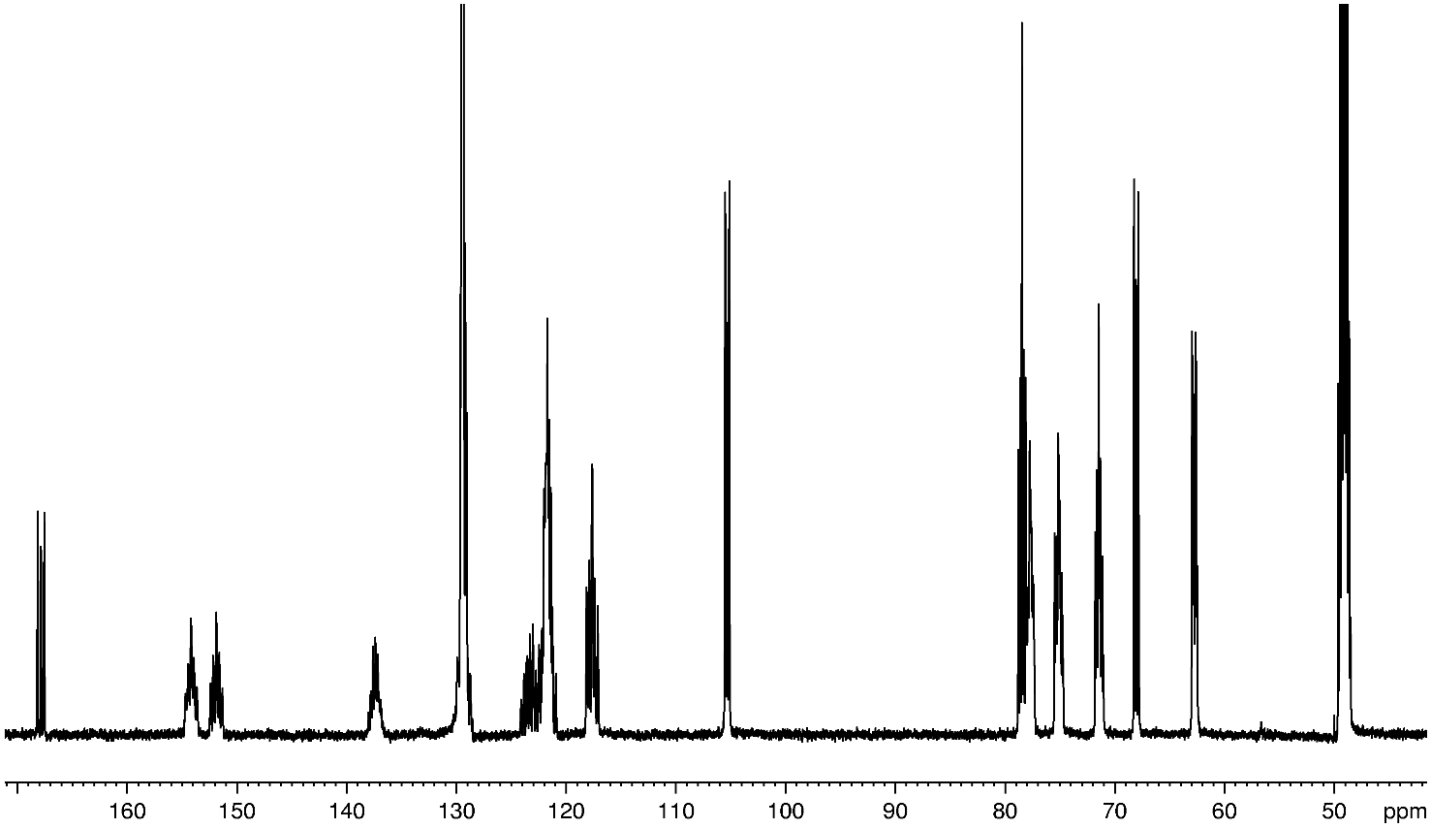
**

**Fig. 31**: [U-^13^C]tremulacin, ^13^C-NMR spectrum (MeOH-d_3_)

**
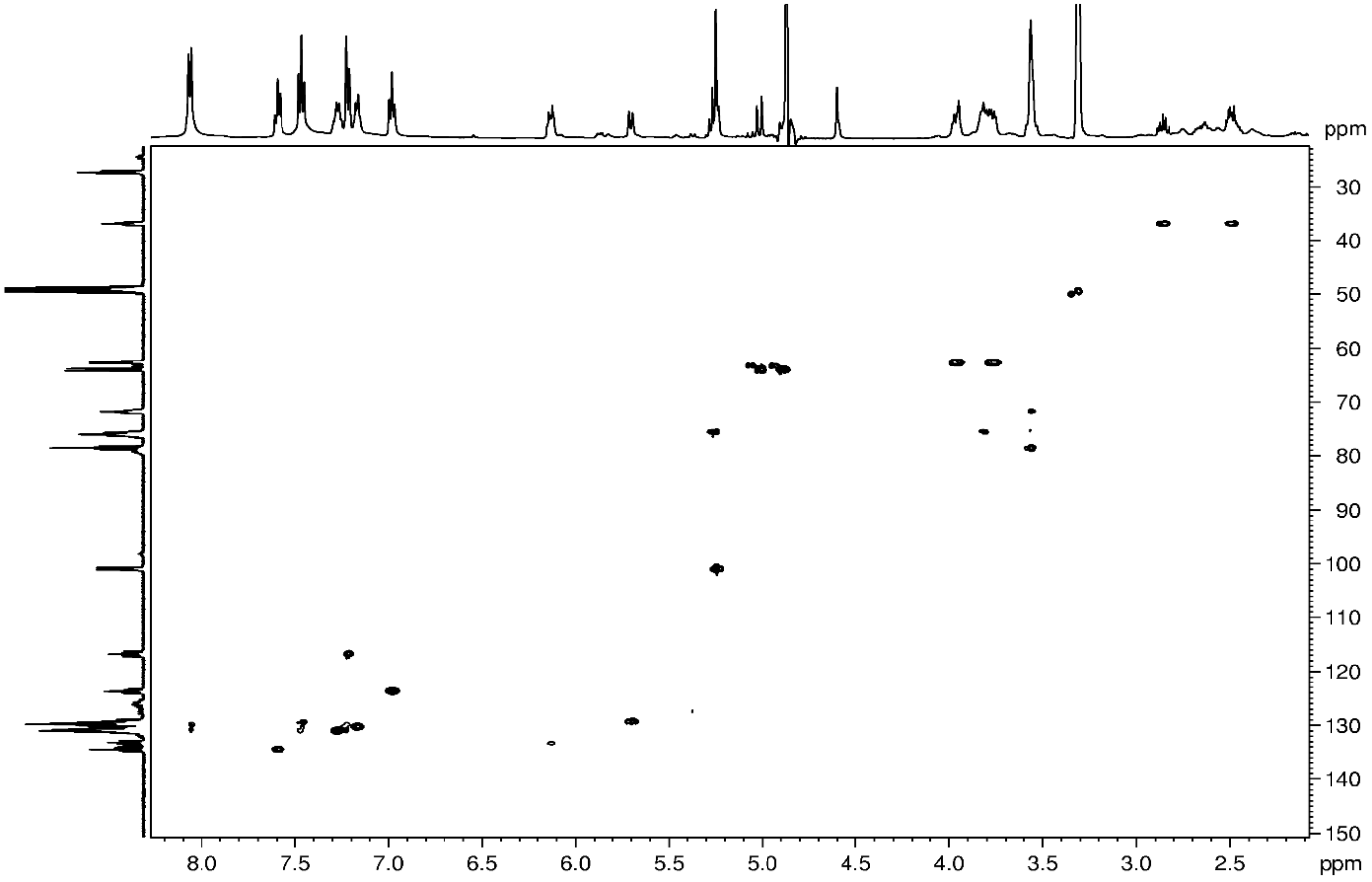
**

**Fig. 32**: [U-^13^C]tremulacin, ^1^H-^13^C HSQC spectrum (MeOH-d_3_)


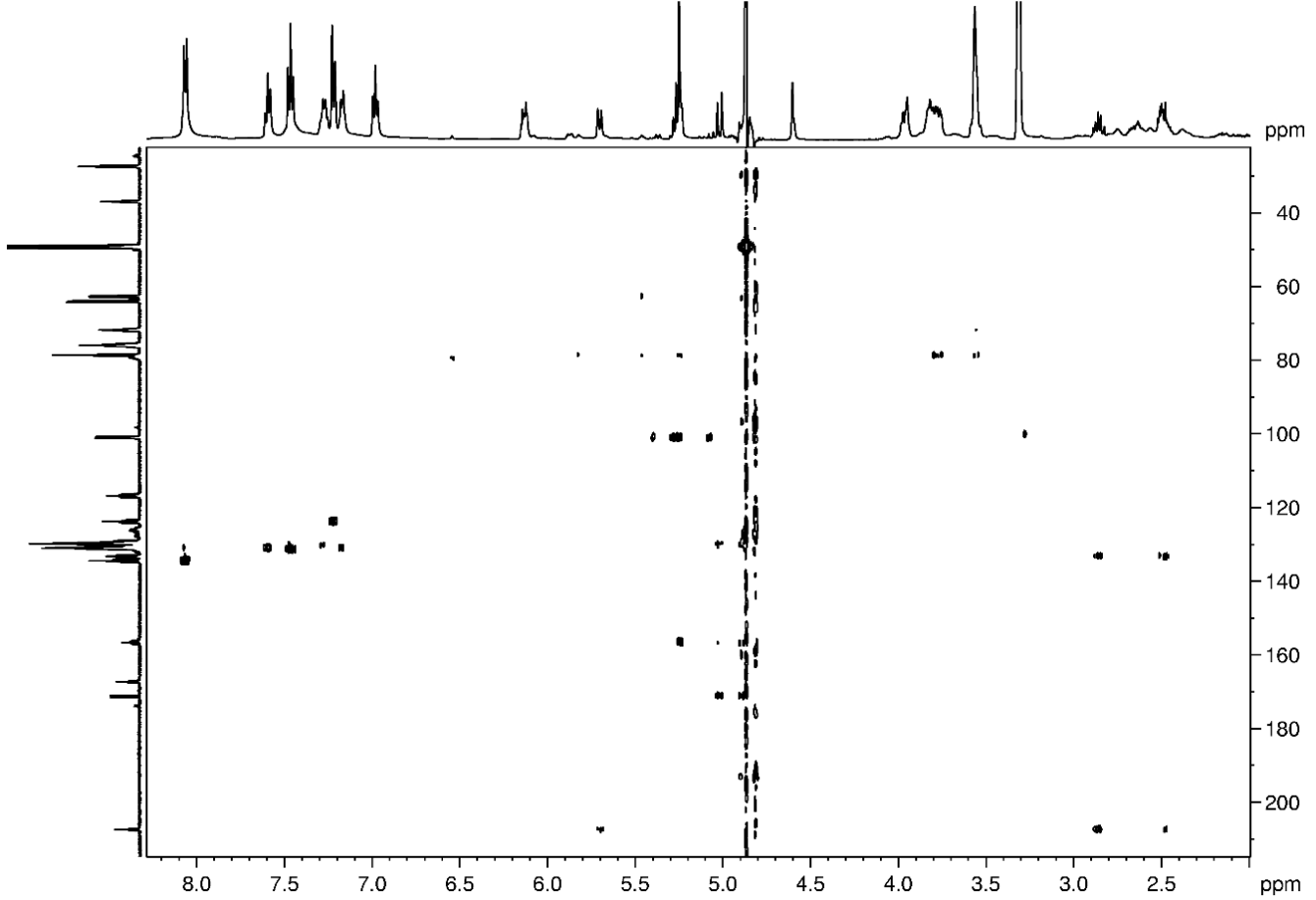


**Fig. 33**: [U-^13^C]tremulacin, ^1^H-^13^C HMBC spectrum (MeOH-d_3_)


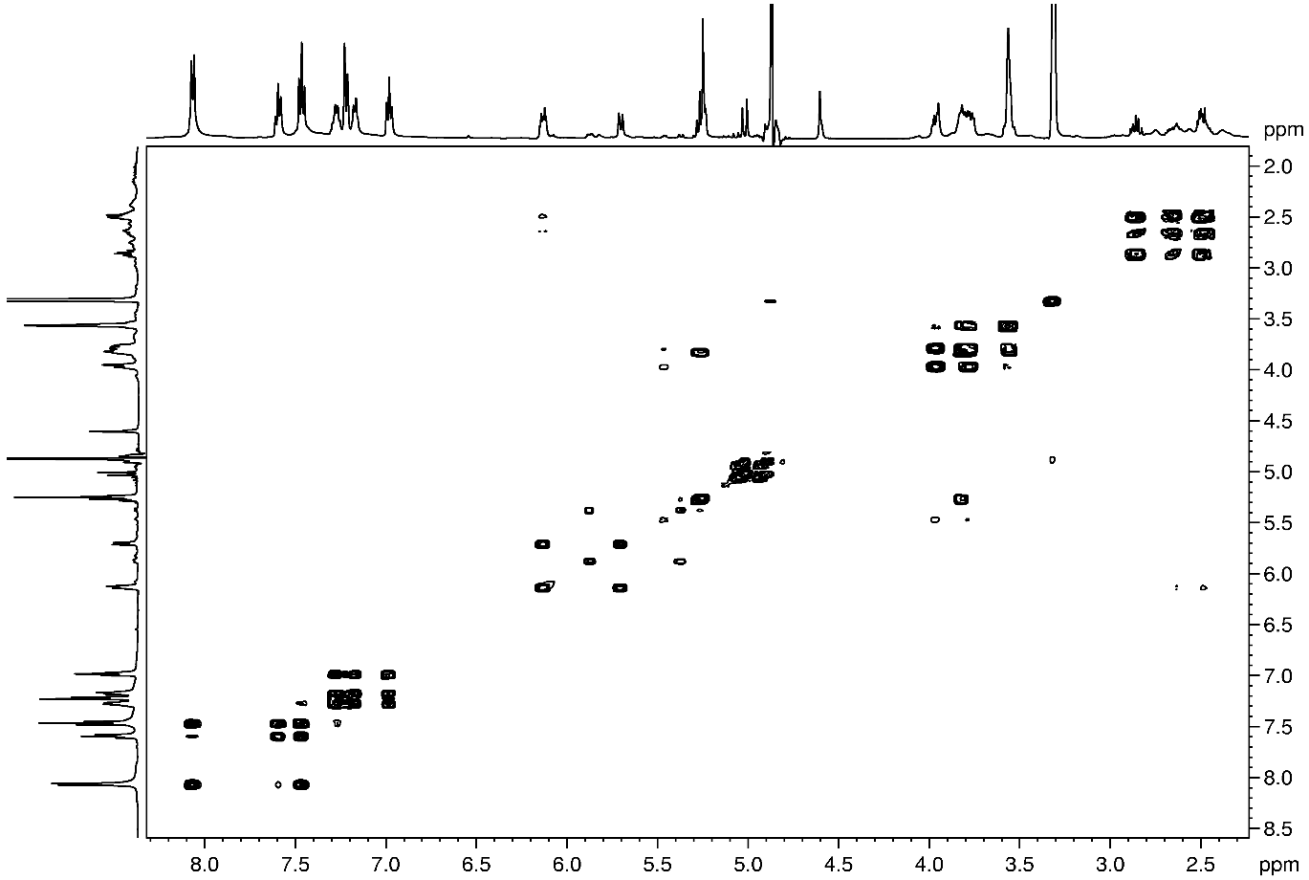


**Fig. 34**: [U-^13^C]tremulacin, ^1^H-^1^H COSY spectrum (MeOH-d_3_)


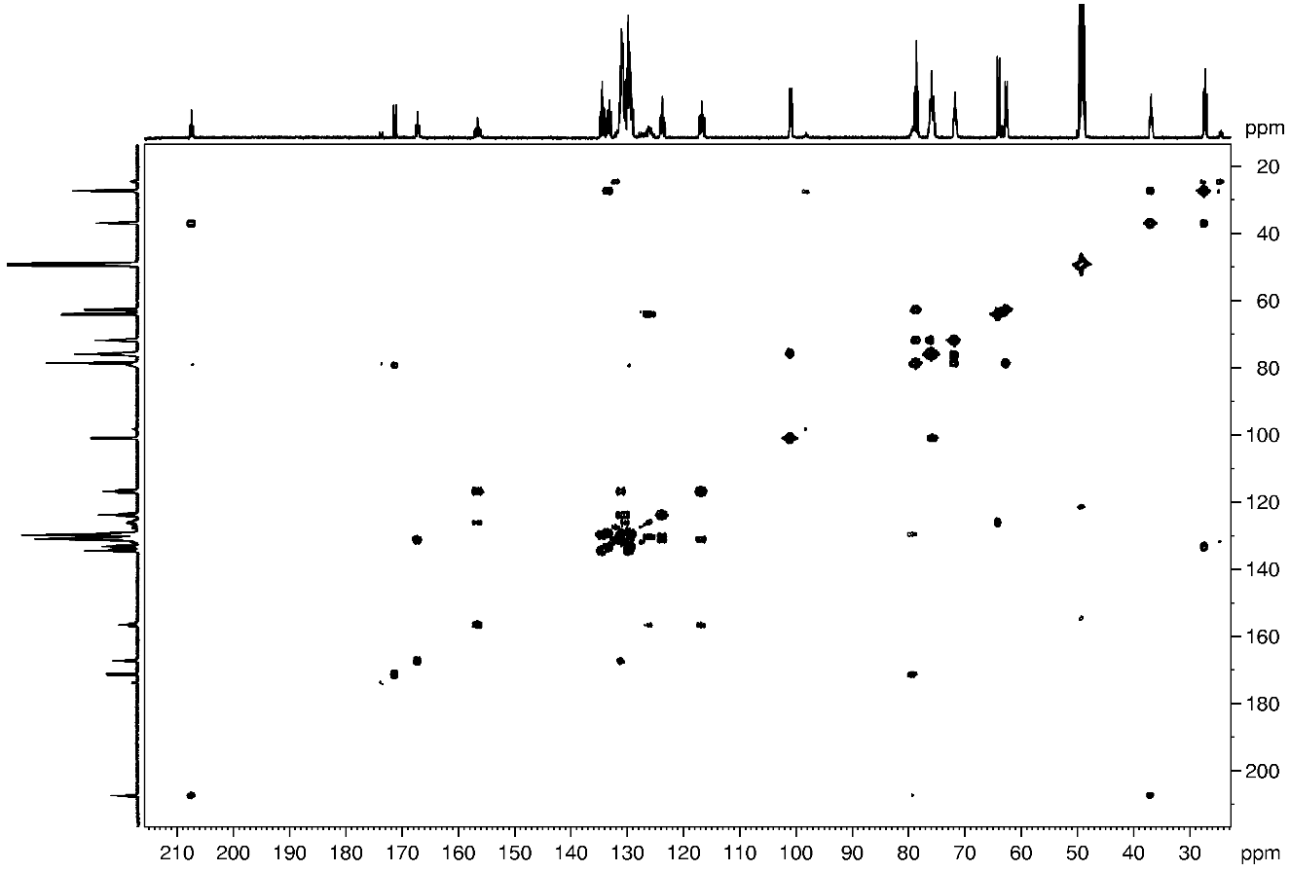


**Fig. 35**: [U-^13^C]tremulacin, ^13^C-^13^C COSY spectrum (MeOH-d_3_)


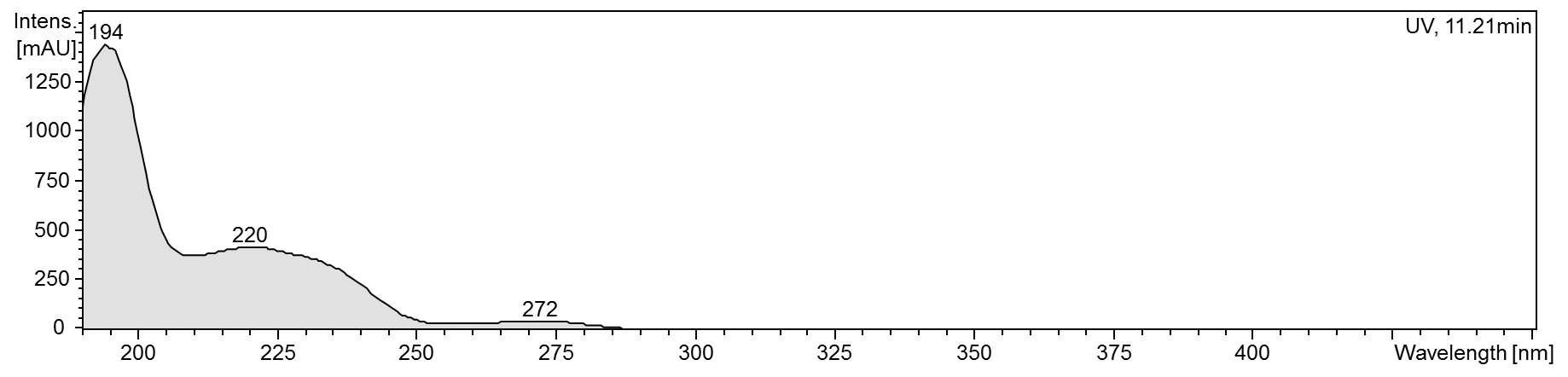


**Fig. 36**: [U-^13^C] tremulacin, UV spectrum from HPLC-DAD

# Decomposition of salicortinoids at pH 7.8


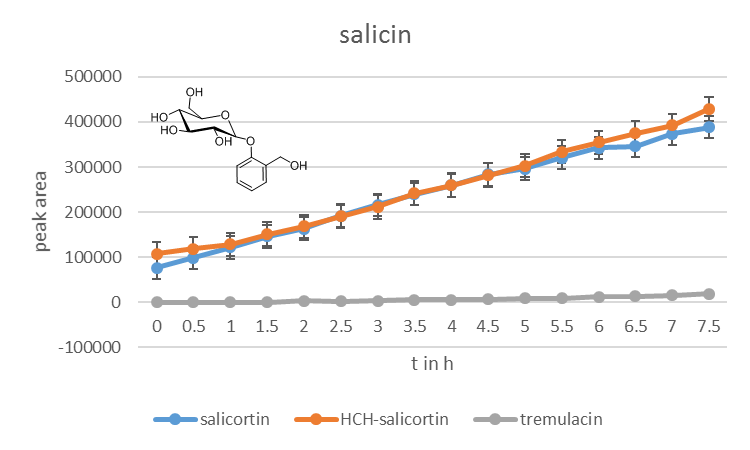


**Fig. 37**: Peak area of salicin generated by benzylic ester cleavage of salicortin HCH-salicortin (by benzylic ester cleavage), and tremulacin (by benzylic and glycosidic ester cleavage) during decomposition experiments (pH 7.8)

#


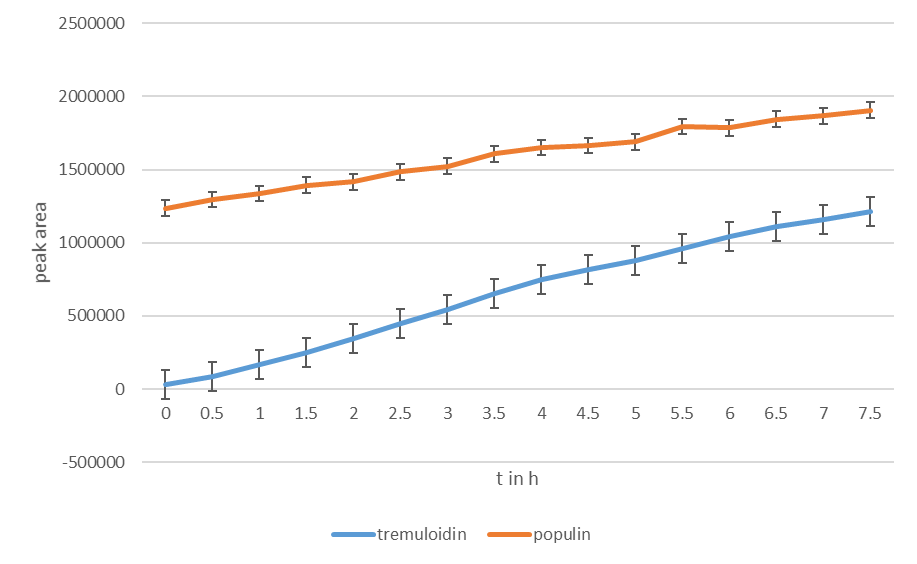


**Fig. 38**: peak area of tremuloidin and populin liberated by benzylic ester cleavage from tremulacin during decomposition experiments (pH 7.8)

**Fig. 39**: Catechol concentration calibration. The concentration of catechol was calculated as follows:
From the determination of salicortin decomposition we calculated the amount of intact salicortin based on UV data. From the difference of decomposed salicortin to intact salicortin we deduced the concentration of catechol formed. This value was correlated with the integral value of the ^12^C signal in the mass spectrum for catechol.

# Gut homogenate incubation experiments


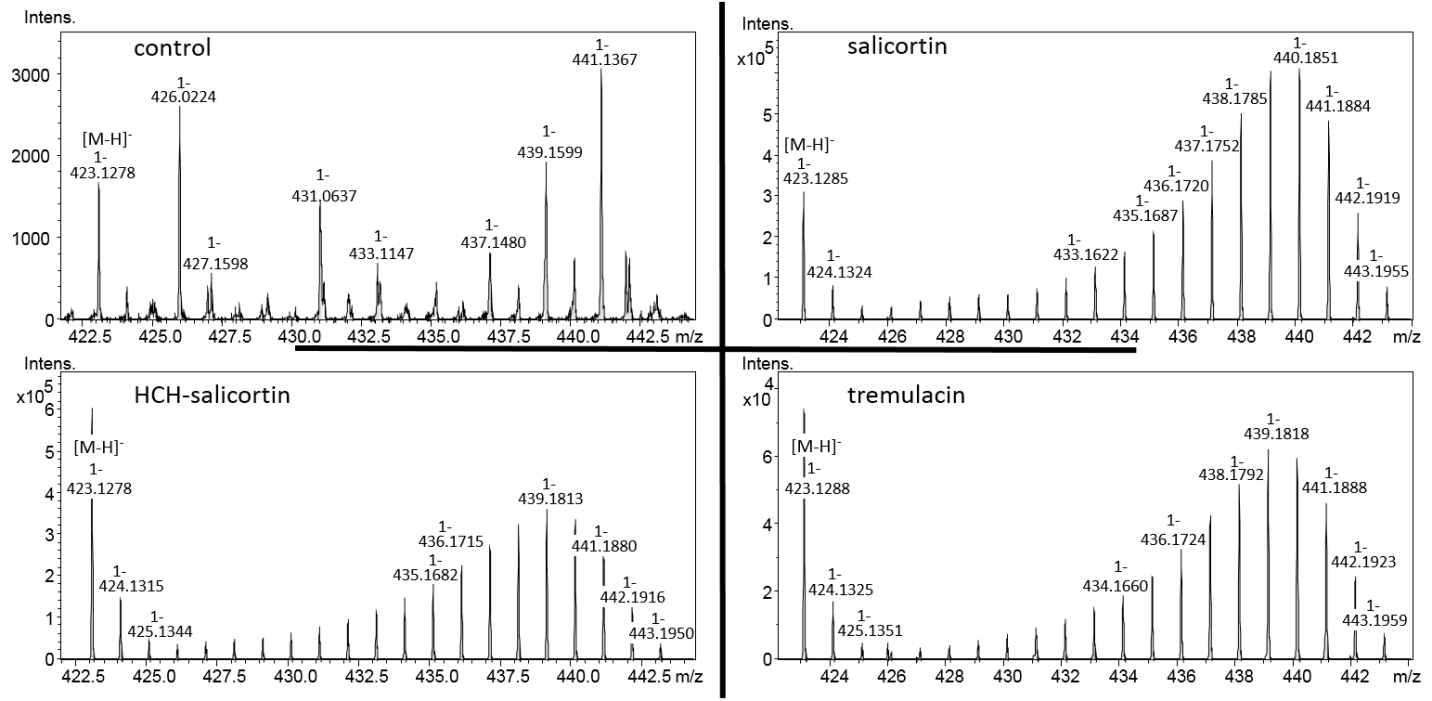


**Fig. 40**: Labelling grade of salicortin (**1**) during gut homogentate incubation


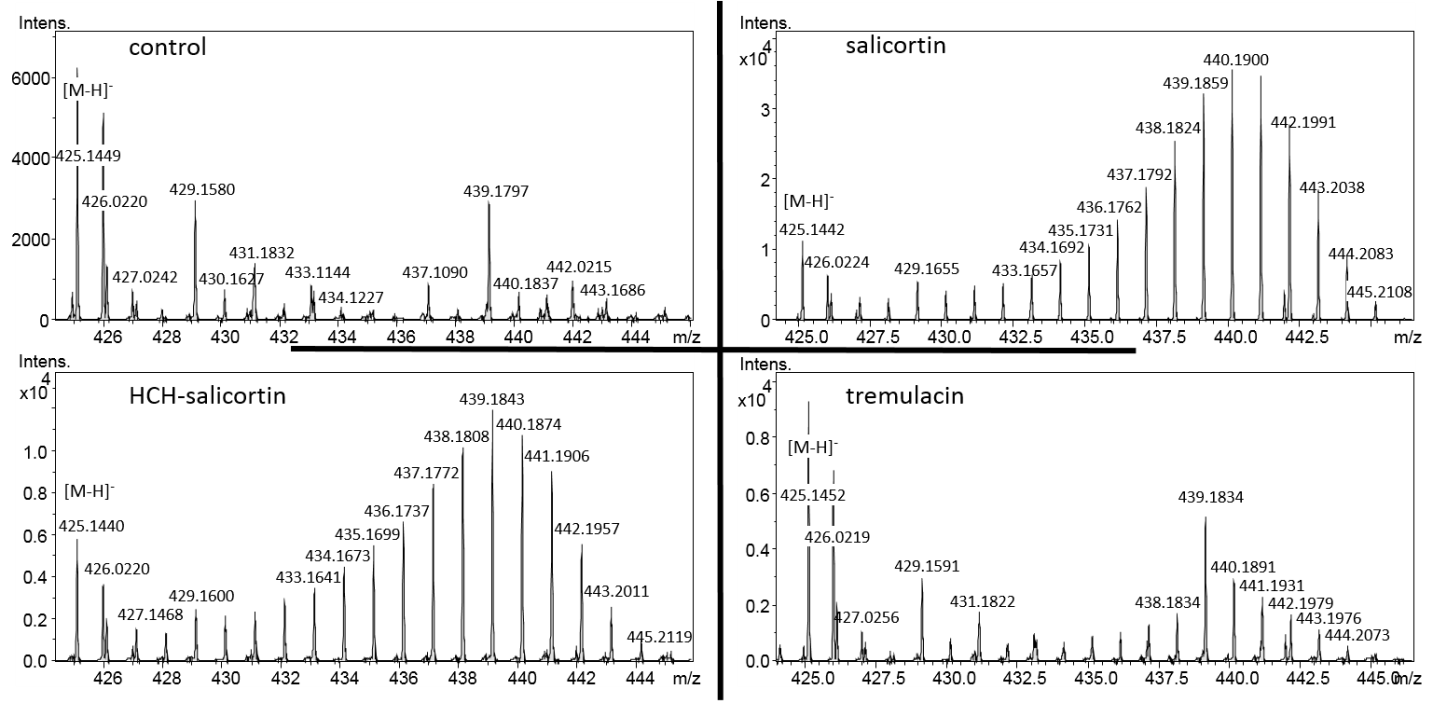


**Fig. 41**: Labelling grade of salicortinol (**4**) during gut homogenate incubation


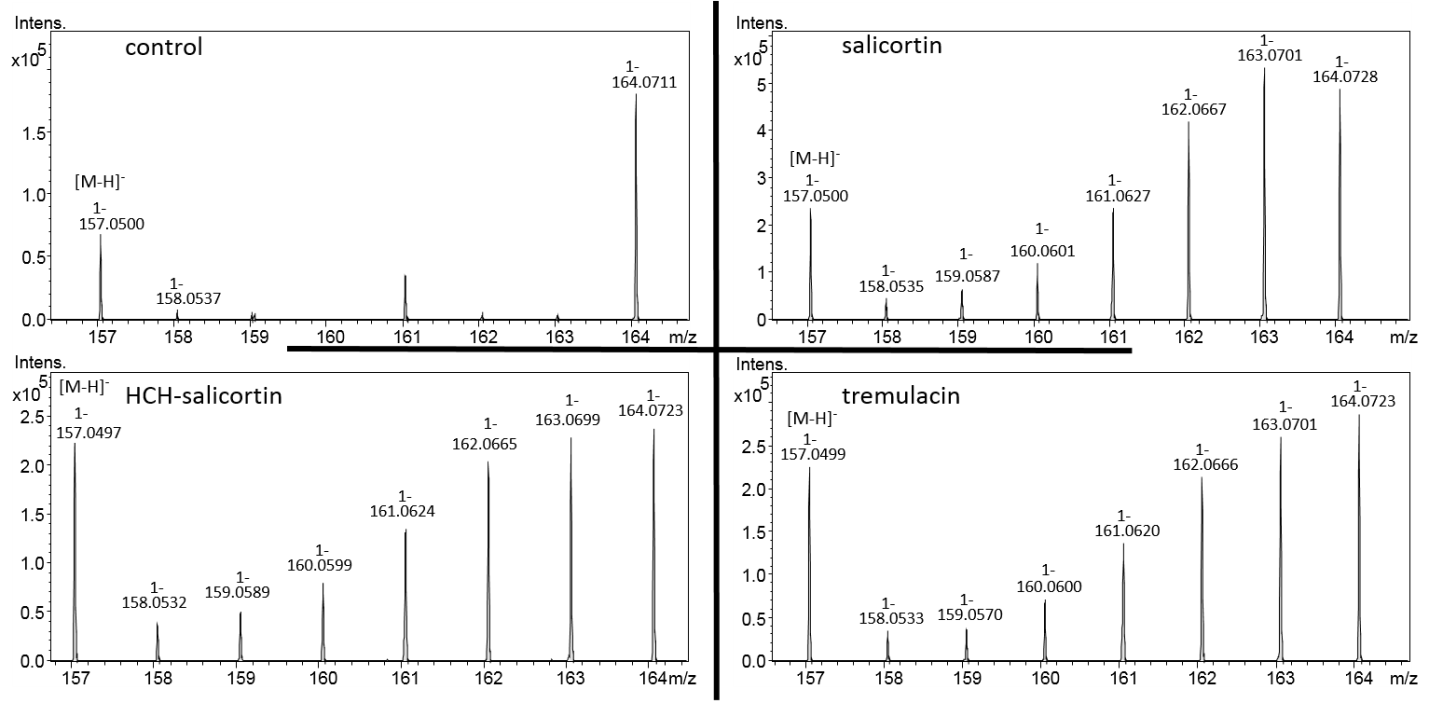


**Fig. 42**: Labelling grade of DHCH (**14**) during gut homogenate incubation


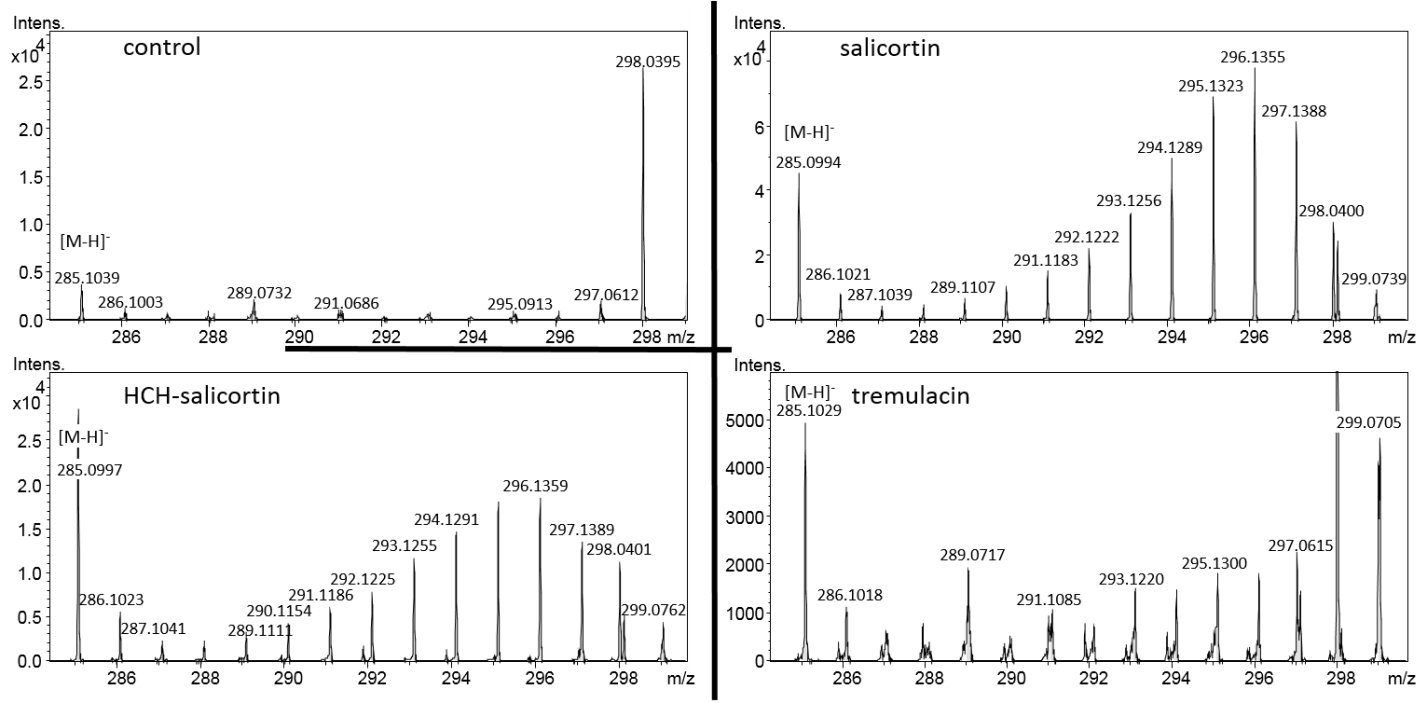


**Fig. 43**: Labelling grade of salicin (**10**) during gut homogenate incubation


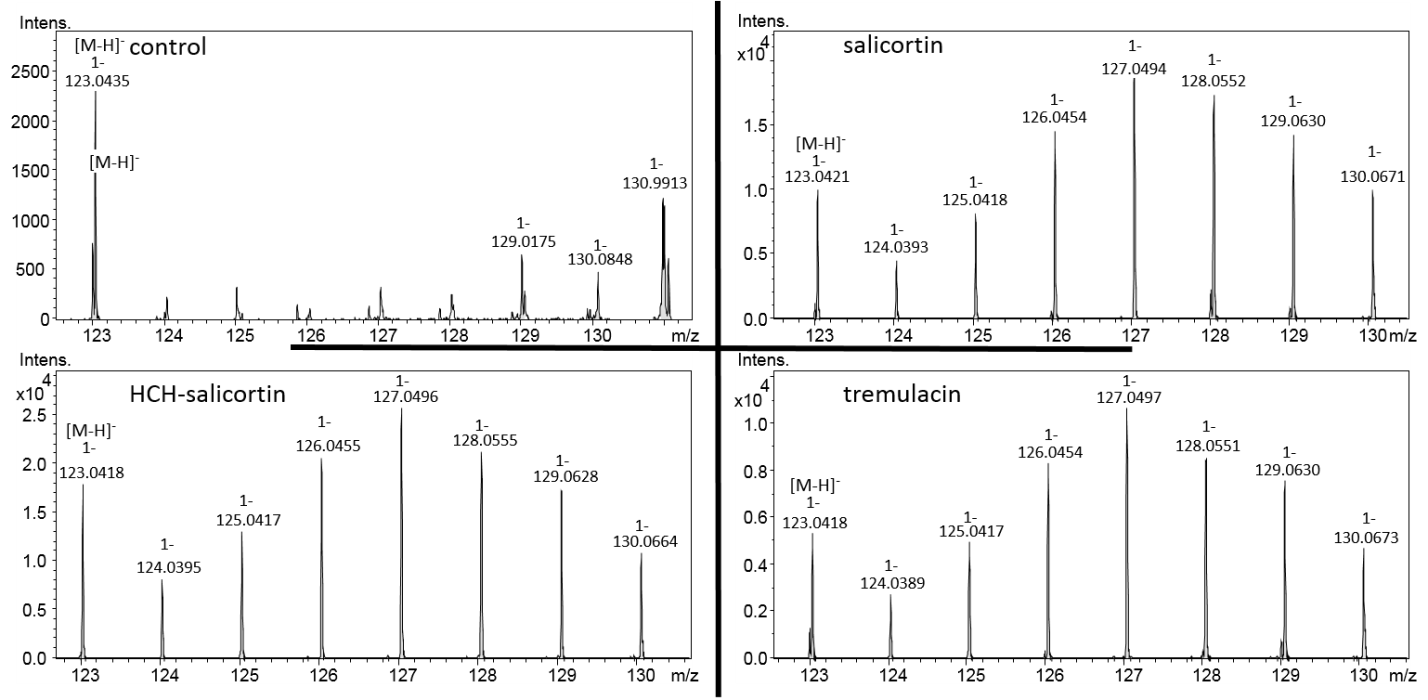


**Fig. 44**: Labelling grade of saligenin (**7**) during gut homogenate incubation


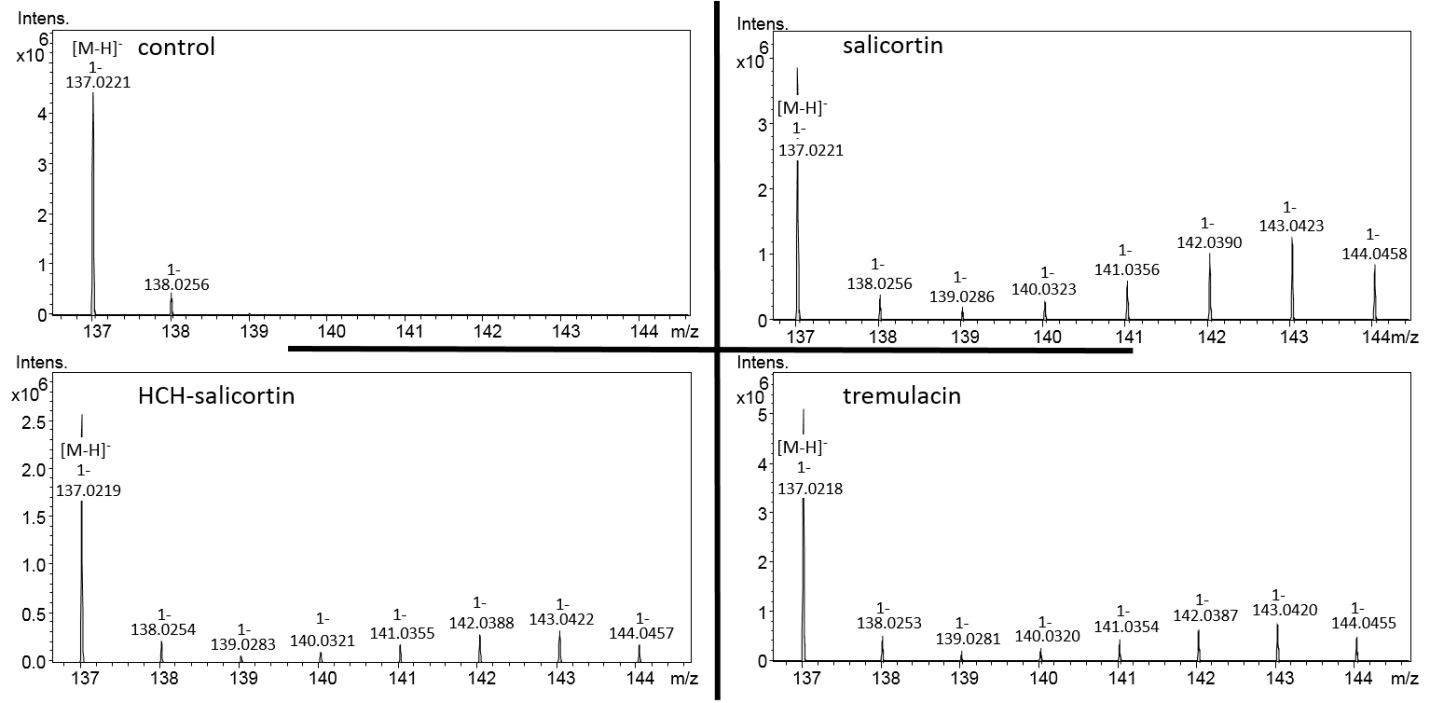


**Fig. 45**: Labelling grade of salicylic acid (**15**) during gut homogenate incubation


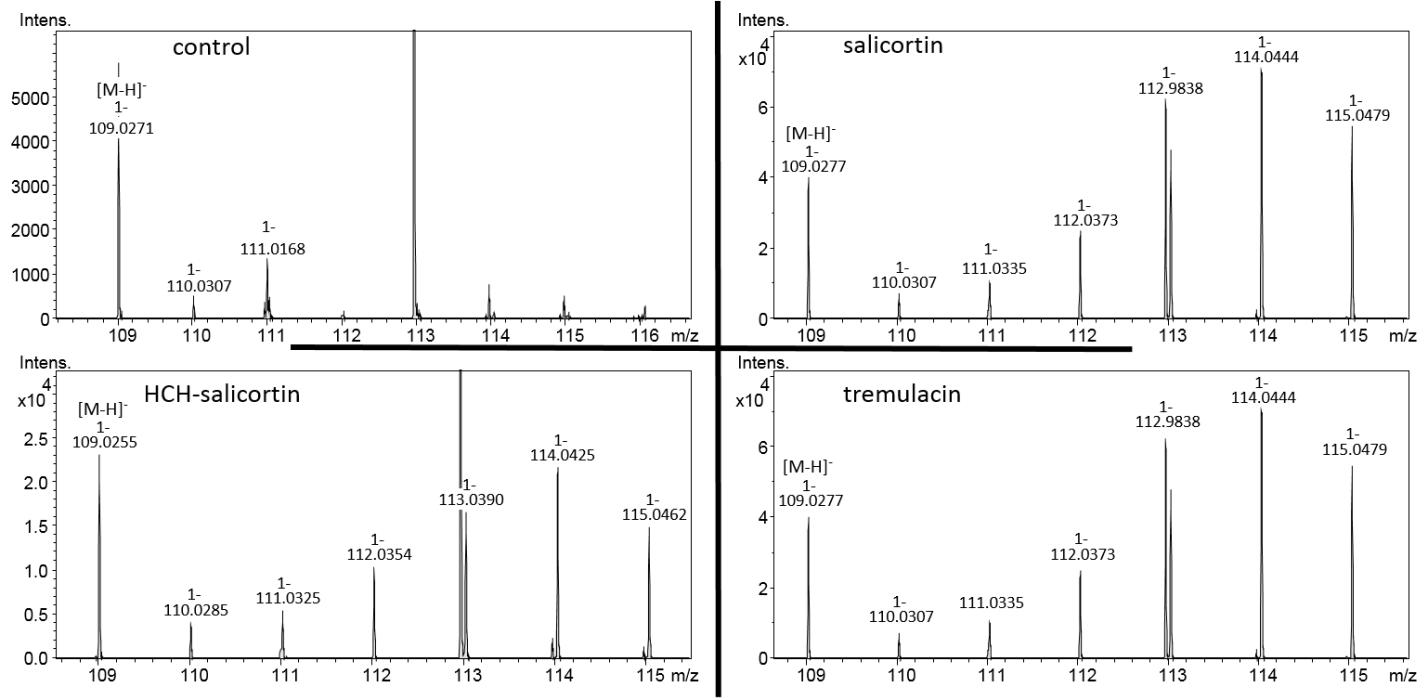


**Fig. 46**: Labelling grade of catechol (**13**) during gut homogenate incubation


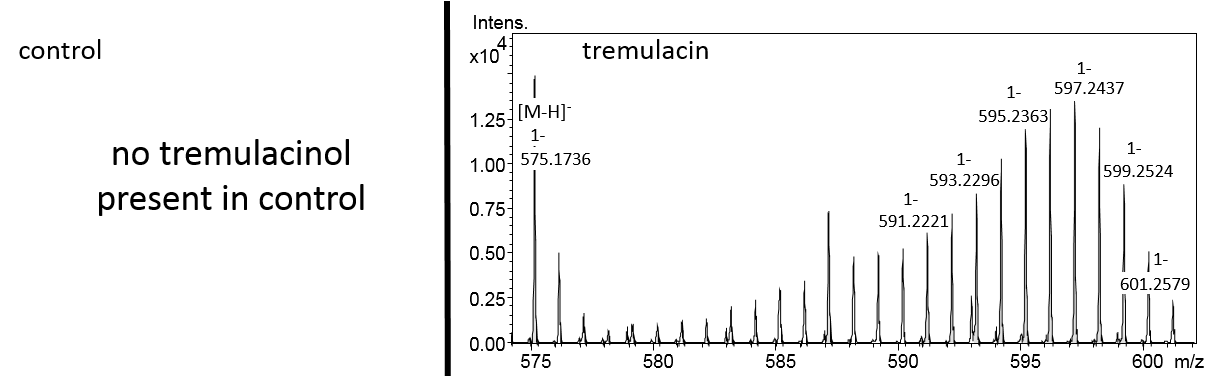


**Fig. 47**: Labelling grade of tremulacinol (**5**) during gut homogenate incubation


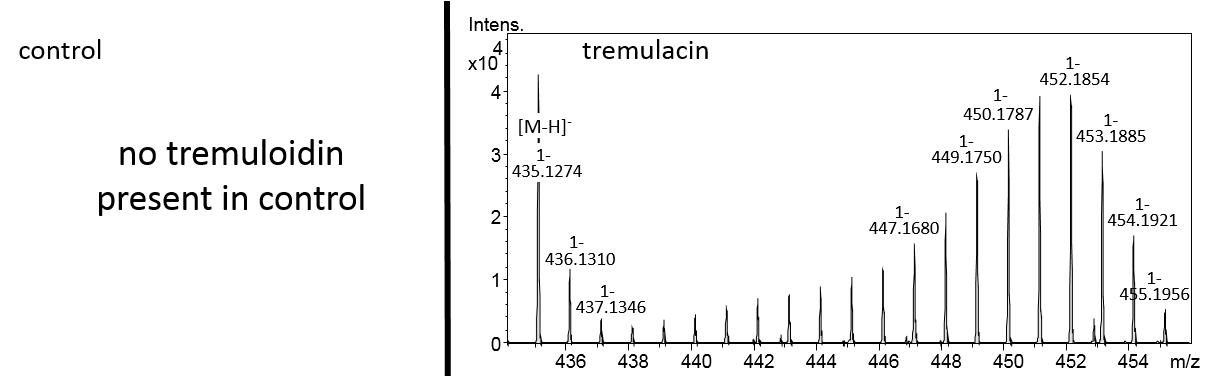


**Fig. 48**: Labelling grade of tremuloidin (**11**) during gut homogenate incubation


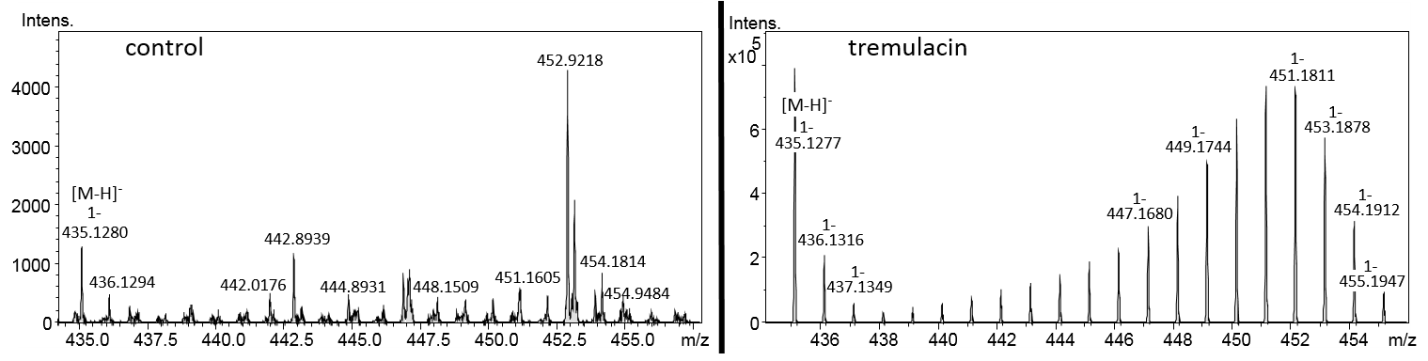


**Fig. 49**: Labelling grade of populin (**12**) during gut homogenate incubation


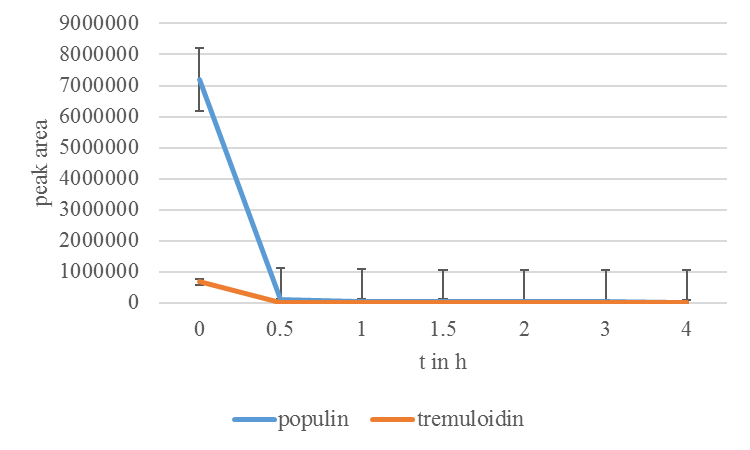


**Fig. 50**: peak area of populin (blue) and tremuloidin (orange) during gut homogenate incubation experiment

# HPLC-HR-ESI-MS Chromatogram of methanol extract of *P. tremula x tremuloides* leaves


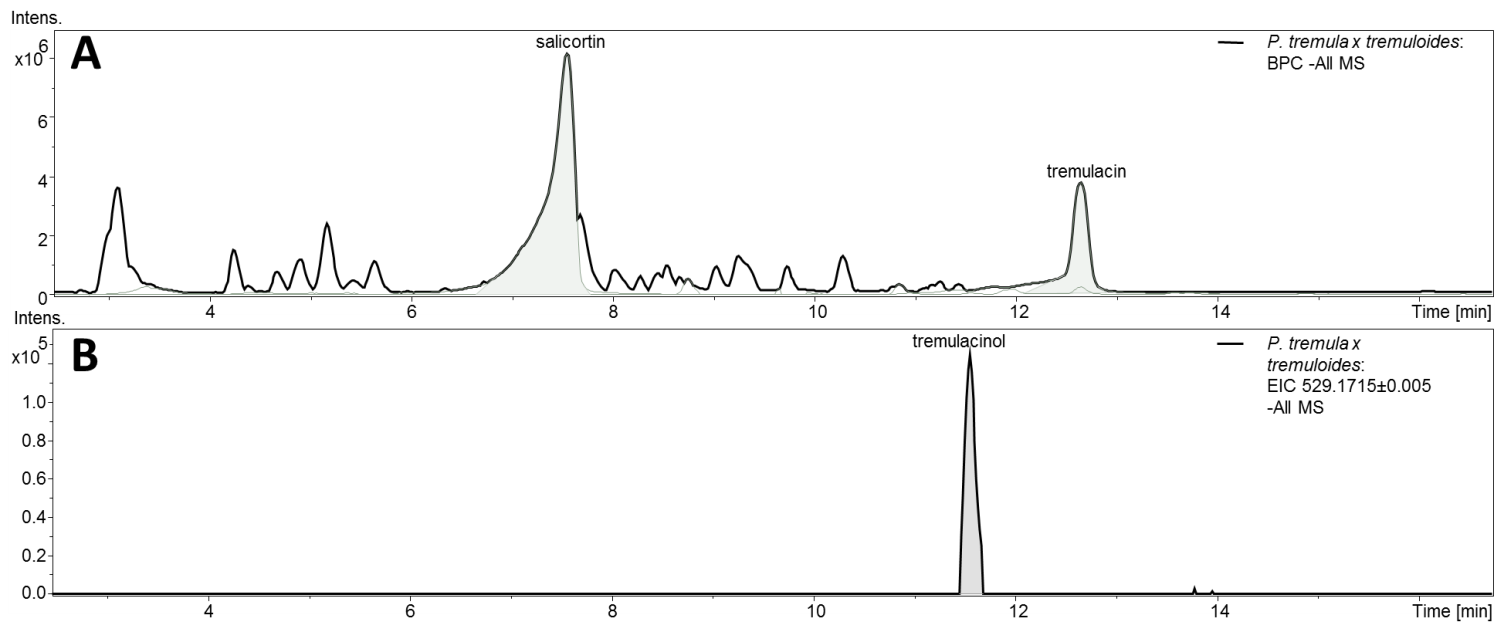


**Fig. 51**: **A**: base peak chromatogram of P. tremula x tremuloides MeOH leaf extract, c = 1 mg/mL. **B**: Extracted ion chromatogram (m/z = 529.1715, calc. mass for tremulacinol and 6’-O-benzoyl-salicortinol) of P. tremula x tremuloides MeOH leaf extract

# HPLC-HR-ESI-MS Chromatogram of methanol extract of *C. vinula* frass after feeding on *P. tremula x tremuloides* leaves


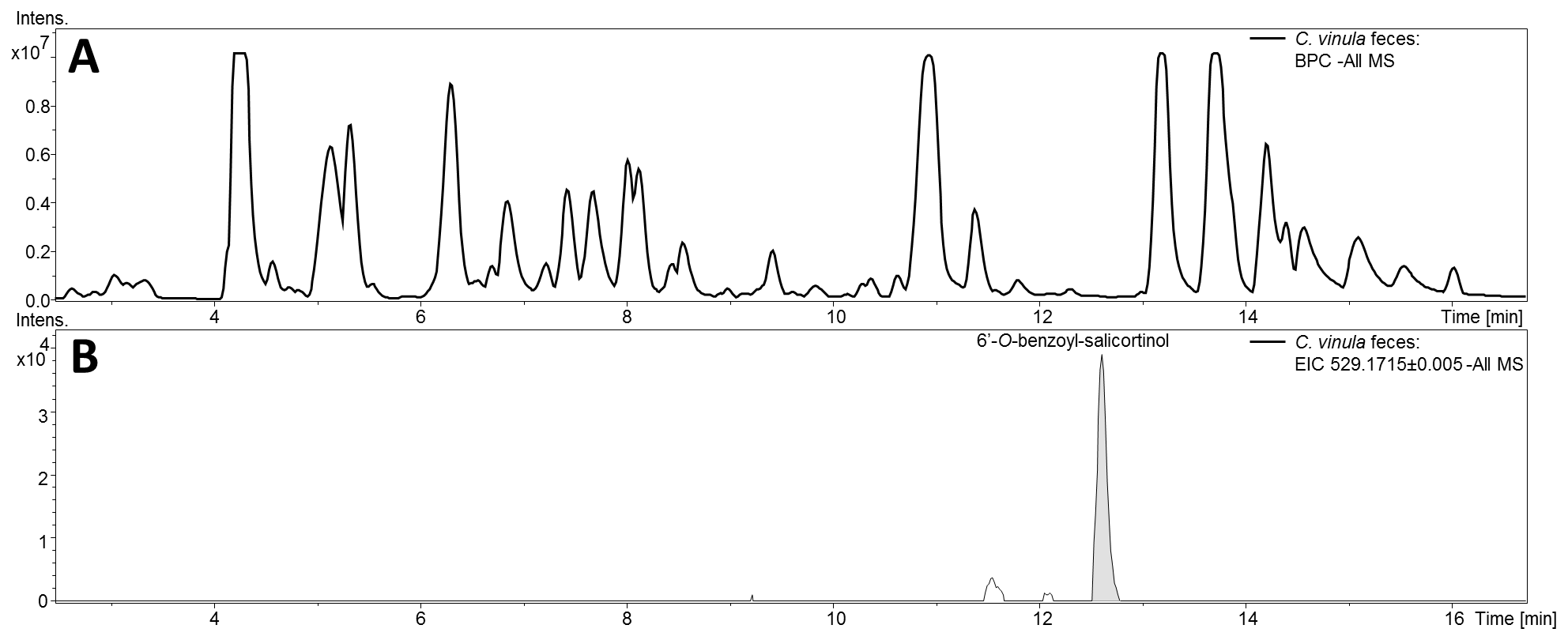


**Fig. 52**: **A**: Base peak chromatogram of C. vinula (5^th^ instar) MeOH feces extract after feeding on P. tremula x tremuloides foliage, c = 1 mg/mL. **B**: Extracted ion chromatogram (m/z = 529.1715, calc. mass for tremulacinol and 6’-O-benzoyl-salicortinol) of C. vinula (5^th^ instar) MeOH feces extract after feeding on P. tremula x tremuloides foliage

# Mechanism of DHCH transformation to salicylic acid

**Fig. 53**: Dehydration and subsequent auto-oxidation of DHCH to salicylic acid under acidic conditions and to salicylate under alkaline conditions. In the first step, the hydroxy group at position one is protonated and subsequently dehydrated. The resulting intermediate, here represented as tautomeric structures, is further converted by auto-oxidation to form salicylic acid. Under alkaline conditions, the first step is the dissociation of the carboxylic acid. Due to the close proximity of the 2-OH group, a hydrogen bond between the carboxylate ion and the alcohol function can be formed, stabilizing the deprotonated carboxyl function. In the next step, the 1-OH group is protonated and the molecule undergoes dehydration. The resulting intermediate is then auto-oxidized to salicylate. Salicylic acid formation from DHCH is fastest in acidic media.

# Lepidoptera used in this study


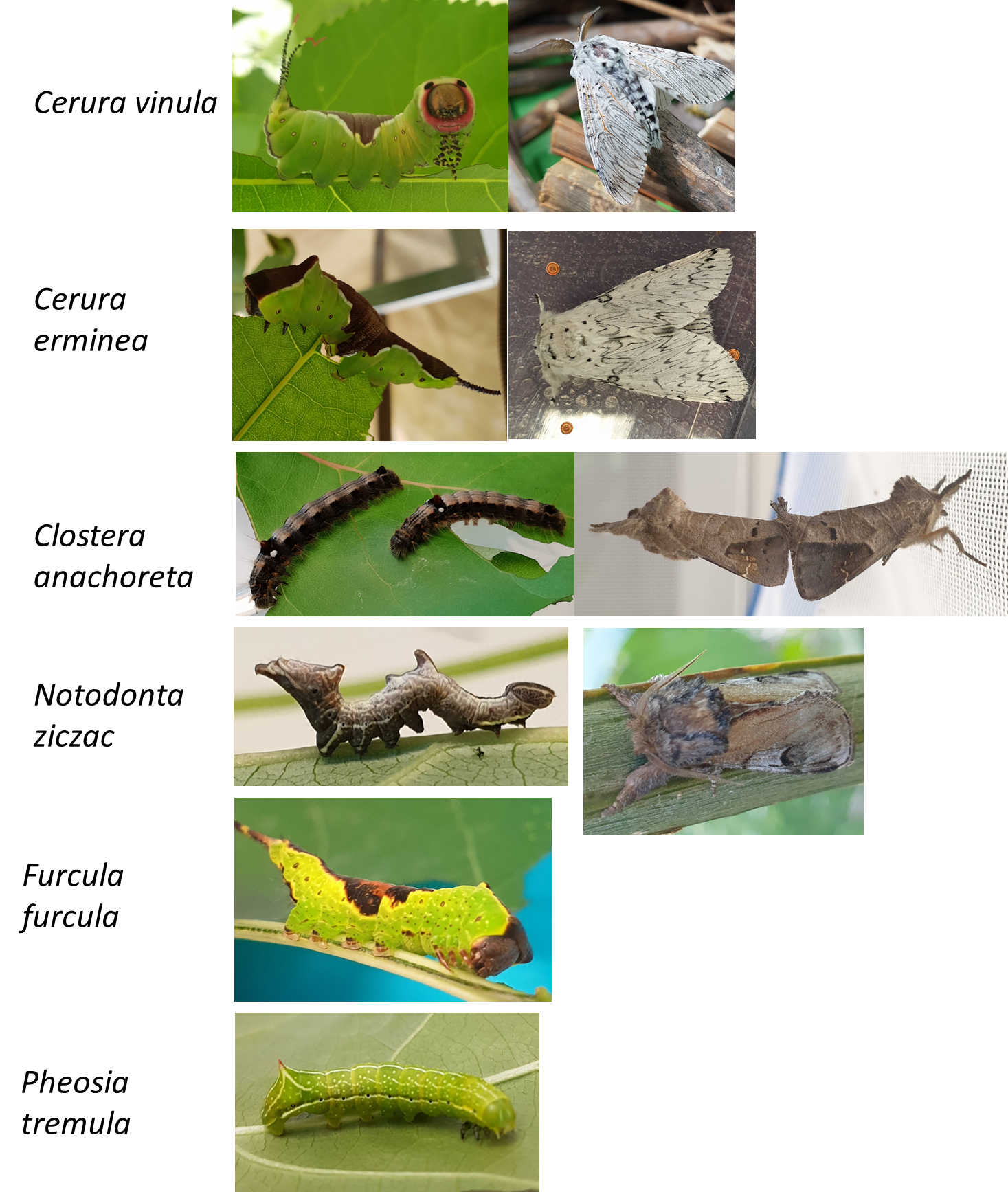


**Fig. 54:** Notodontidae species used in this study, authentic pictures
